# Supplementary material for: Study protocol for THINK: a multinational open-label phase I study to assess the safety and clinical activity of multiple administrations of NKR-2 in patients with different metastatic tumour types
Source: BMJ Open. 2017 Nov 12;7(11):e017075. doi: 10.1136/bmjopen-2017-017075 (PMC5695348; doi:10.1136/bmjopen-2017-017075)
Supplement: Author's manuscript [file bmjopen-2017-017075.draft_revisions.pdf]

BMJ Open is committed to open peer review. As part of this commitment we make the peer review history of every article we publish publicly available.

When an article is published we post the peer reviewers' comments and the authors' responses online. We also post the versions of the paper that were used during peer review. These are the versions that the peer review comments apply to.

The versions of the paper that follow are the versions that were submitted during the peer review process. They are not the versions of record or the final published versions. They should not be cited or distributed as the published version of this manuscript.

BMJ Open is an open access journal and the full, final, typeset and author-corrected version of record of the manuscript is available on our site with no access controls, subscription charges or pay-per-view fees (http://bmjopen.bmj.com).

If you have any questions on BMJ Open's open peer review process please email editorial.bmjopen@bmj.com

BMJ Open

THINK: A multinational open-label Phase I study to assess the safety and clinical activity of multiple administrations of NKR-2 in patients with different metastatic tumour types

Journal:	*BMJ Open*
Manuscript ID	bmjopen-2017-017075
Article Type:	Protocol
Date Submitted by the Author:	30-Mar-2017

Complete List of Authors:	Lonez, Caroline; Celyad SA,<br>Verma, Bikash; Celyad SA<br>Hendlisz, Alain; Institut Jules Bordet, Medical Oncology Department-GastroIntestinal Unit<br>Aftimos, Philippe; Institut Jules Bordet, Medical Oncology<br>Awada, Ahmad; Institut Jules Bordet, Medical Oncology<br>Van Den Neste, Eric; Universite Catholique de Louvain La Faculte de Medecine<br>Catala, Gaetan; Universite Catholique de Louvain La Faculte de Medecine<br>Machiels, Jean-Pascal; Universite Catholique de Louvain La Faculte de Medecine<br>Piette, Fanny; IDDI SA<br>Brayer, Jason; H Lee Moffitt Cancer Center and Research Institute<br>Sallman, David; H Lee Moffitt Cancer Center and Research Institute<br>Kerre, Tessa; Universitair Ziekenhuis Gent<br>Odunsi, Kunle; Roswell Park Cancer Institute<br>Davila, Marco; H Lee Moffitt Cancer Center and Research Institute; USF Health Morsani College of Medicine<br>Gilham, David; Celyad SA<br>Lehmann, Frédéric; Celyad SA

**Primary Subject Heading**:	Oncology
Secondary Subject Heading:	Immunology (including allergy)
Keywords:	NKG2D, chimeric antigen receptor, CAR-T, solid tumours, NKR-2, multiple injections

SCHOLARONE™ Manuscripts

THINK: A multinational open-label Phase I study to assess the safety and clinical activity of multiple administrations of NKR-2 in patients with different metastatic tumour types

Authors and affiliations

Caroline Lonez[1], Bikash Verma[1], Alain Hendlisz[2], Philippe Aftimos[2], Ahmad Awada[2], Eric Van Den Neste[3], Gaetan Catala[3], Jean-Pascal H. Machiels[3], Fanny Piette[4], Jason B. Brayer[5], David A Sallman[5], Tessa Kerre[6], Kunle Odunsi[7], Marco L Davila[5,8], David E Gilham[1], Frédéric F Lehmann[1]

1. Celyad S.A., Mont-Saint-Guibert, Belgium; 2. Institut Jules Bordet, Université Libre de Bruxelles, Brussels, Belgium; 3. Cliniques Universitaires Saint-Luc, Université Catholique de Louvain, Brussels, Belgium; 4. International Drug Development Institute, Louvain-la-Neuve, Belgium; 5. H. Lee Moffitt Moffitt Cancer Center, Tampa, FL, USA; 6. Ghent University Hospital, Ghent, Belgium; 7. Roswell Park Cancer Institute, Buffalo, NY, USA; 8. Morsani College of Medicine, University of South Florida, Tampa, FL, USA. Correspondence to Dr. Caroline Lonez, Celyad SA, Rue Edouard Belin 2, 1435 Mont-Saint-Guibert, Belgium. Email: clonez@celyad.com. Phone: +32(0)10394182.

Keywords

NKG2D, chimeric antigen receptor, CAR-T, solid tumours, NKR-2, multiple injections

Word count: 5127

For peer review only - http://bmjopen.bmj.com/site/about/guidelines.xhtml

Abstract

Introduction: NKR-2 are autologous T-cells genetically modified to express a chimeric antigen receptor (CAR) comprising a fusion of the NKG2D receptor with the CD3ζ signalling domain, which associates with the adaptor molecule DAP10 to provide co-stimulatory signal upon ligand binding. NKG2D binds eight different ligands expressed on the cell surface of many tumour cells and which are normally absent on non-neoplastic cells. In preclinical studies, NKR-2 demonstrated long-term antitumour activity towards a breadth of tumour indications, with maximum efficacy observed after multiple NKR-2 administrations. Importantly, NKR-2 targeted not only tumour cells but also tumour neovasculature and the local tumour immunosuppressive microenvironment and this mechanism of action of NKR-2 was established in the absence of pre-conditioning.

Methods and analysis: This open-label Phase I study will assess the safety and clinical activity of NKR-2 treatment administered three times, with a two-week interval between each administration, in different tumour types. The study will contain two consecutive segments: a dose escalation phase followed by an expansion phase. The dose escalation study involves two arms, one in solid tumours (5 specific indications) and one in haematological tumours (2 specific indications), and will include 3 dose-levels in each arm: $3x10^8$, $1x10^9$ and $3x10^9$ NKR-2 per injection. Upon the identification of the recommended dose in the first segment, based on dose-limiting toxicity occurrences, the study will expand to seven different cohorts examining the seven different tumour types separately. Clinical responses will be determined according to standard RECIST criteria for solid tumours, or international working group response criteria in haematological tumours.

For peer review only

Ethics approval and dissemination: Ethical approval has been obtained at all sites. Written informed consent will be taken from all participants. Results of this study will be disseminated through presentation at international scientific conferences and reported in peer-reviewed scientific journals.

Trial registration: NCT03018405, EudraCT 2016-003312-12.

For peer review only

For peer review only - http://bmjopen.bmj.com/site/about/guidelines.xhtml

Strengths and limitations of this study

- NKG2D-based chimeric antigen receptor (CAR)-T cells retrovirally modified, called NKR-2, exploiting the multiple ligand targeting capability of human NKG2D and display a unique mode of action.
- Standard dose escalation phase and an expansion phase to investigate for the first time the safety and initial clinical activity of NKR-2 against a broad range of tumour indications.
- Short-term persistence and limited expansion of NKR-2 after infusion.

For peer review only - http://bmjopen.bmj.com/site/about/guidelines.xhtml

Introduction

CAR T-cell therapy

The Chimeric Antigen Receptor (CAR) is an artificial construct that endows T-cells with pre-determined specificity [1]. Prior to adoptive transfer they have to be expanded *in vitro* to reach therapeutically sufficient numbers. In this manner, large numbers of tumour antigen specific T-cells can be rapidly generated for use in adoptive cell therapy. The CAR itself has developed through several generations but has largely followed the configuration of extracellular antigen binding domain (most commonly a single-chain variable fragment (scFv) derived from an antibody), a transmembrane domain and intracellular signalling domains. At present, second generation formats tend to predominate featuring a co-stimulatory signalling domain (*e.g.* CD28, 4-1BB) fused to a T-cell activating domain (e.g. CD3ζ) [1, 2].

The CD19 specific CAR provides the paradigm against which other CAR approaches are currently measured due to the impressive clinical responses seen in early phase clinical trials targeting B-cell malignancies. Reports of initial objective clinical responses in 70-90% of patients with advanced chemo-resistant B-cell leukemia have fueled interest in the area with the number of CD19-targeted CAR T-cell trials rapidly rising within the last few years [3, 4]. The CD19-CAR T-cell therapy approach is based on a "*transplant procedure-like*" approach, *i.e.*, a single CAR T-cell infusion in pre-conditioned (lymphodepleted) patients and for which persistence, expansion and survival of the transferred T-cells are crucial [3, 4]. All pre-conditioning regimens follow a similar concept where there is a transient depletion of circulating lymphoid cell populations to provide 'space' for the adoptively transferred T cells to occupy [5, 6]. Additional beneficial effects of pre-conditioning include the production of homeostatic cytokines and dampening of suppressive factors such as regulatory T-cells thereby providing a window of immunologic opportunity to target the tumour.

For peer review only

For peer review only - http://bmjopen.bmj.com/site/about/guidelines.xhtml

However, single-target therapy may select for and spur escape variants by clonal selection or antigen loss. Several CD19-specific therapies indeed evidenced cases of relapse resulting from CD19 antigen loss in leukemia cells post-CD19-specific CAR T-cell therapy [7-10]. Furthermore, these therapies are also associated with significant levels of toxicity [11, 12] including cytokine release syndrome (CRS), neurotoxicity and on-target off-tumour toxicity against normal cells (*e.g.* CD19-specific CAR T-cells also target normal B-cells expressing CD19, causing prolonged B-cell aplasia and hypogammaglobulinemia [13, 14]). Moreover, anaphylaxis reaction to the foreign components of the CAR (*e.g.* most CARs use scFv from murine origin which might potentially induce transgene-specific humoral and cellular immune responses against the murine domain [15-17]) will impact drastically the safety of multiple injection schedule [18]. The risk/benefit is clearly biased towards benefit since these patients have highly advanced cancer with little, if any, alternative effective therapeutic option. However, this toxicity does provide a level of patient selection that impacts recruitment onto current trials and the wider applicability for the general patient population.

By contrast, outside of the B-cell malignancy setting, the CAR T-cell approach has met with much reduced levels of clinical success [2]. Notably, apart from CD19, there are only a few examples of normal cell lineages that can be safely eliminated along with malignant cells that share the targeted antigen, even within haematologic malignancies. Targeting an antigen on tumour cells that is also present on normal cells, *e.g.* on the myeloid lineage, could lead to on-target off-tumour toxicity and serious side effects related to therapy [19]. Early studies targeting solid tumours reported little evidence of clinical effect while there was some evidence of on-target, off-tumour toxicity seen using CAR targeting carbonic anhydrase-IX [16, 20] in renal cell carcinoma or Her2/neu-specific CAR-Ts in colorectal cancer [21]. Investigators are therefore still seeking for the ideal target antigen that should be highly

For peer review only - http://bmjopen.bmj.com/site/about/guidelines.xhtml

overexpressed on tumour cells as compared with normal cells. Whilst some recent studies have shown limited clinical effect but nowhere near to that seen with CD19 CAR T-cell therapy [3, 22], the challenges posed by solid tumours are numerous and have been extensively reviewed recently [23-26]. In brief, these include the on-target off-tumours impact related to expression of the target on normal tissues, the need for trafficking into the tumour microenvironment and tumour stroma, tumour heterogeneity and the requirement to overcome the hostile immune suppressive tumour microenvironment, potentially leading to CAR-T exhaustion, clearly requiring the further development of the basic CAR approach [27]. Several options including third and fourth generation CARs (*e.g.* Armored CAR, TRUCKs), logic gated activation strategies or the combination of CAR-Ts with T-cell checkpoint inhibitors are currently under development to solve these problems.

NKR-2

NKR-2 are autologous T-cells genetically modified to express a CAR comprising a fusion of the full-length Natural Killer Group 2D (NKG2D) receptor with the CD3ζ signalling domain. NKG2D is a C-type, lectin-like, type II transmembrane glycoprotein activating receptor expressed in humans on Natural Killer (NK), Natural Killer T, activated $CD8^+$ T-cells and, under certain conditions, some $CD4^+$ and γδ+ T-cell subsets [28] and functioning to target and kill cancer or infected cells. In humans, NKG2D is expressed as a homodimer that associates with the adaptor molecule DNAX-activating protein of 10 kDa (DAP10) to form a hexameric structure [29]. The interaction with DAP10 is essential to stabilize the surface expression of NKG2D in T-cells and to mediate signal transduction and cellular activation upon ligand recognition [30]. Therefore, whilst the NKR-2 CAR format suggests a 'first generation' configuration, the NKG2D structural component, via its interaction with endogenous DAP10, engages native T-cell costimulatory signalling apparatus to providing a sufficient co-

For peer review only - http://bmjopen.bmj.com/site/about/guidelines.xhtml

stimulatory signal which complement the primary CD3ζ vector-derived signal, indicating that the NKR-2 actually works rather like a 'second generation' CAR (Figure 1). Furthermore, the requirement for endogenous DAP10 expression for stable expression of NKG2D within cells defines an additional embedded safety measure to restrict NKR-2 vector expression and function specifically to cells expressing native DAP10 (*e.g.* T-cells).

In humans, eight NKG2D ligands have been identified, encoded by different loci on two different arms of the chromosome 6 and corresponding to two main families: the Major Histocompatibility Class (MHC) class I chain related proteins (MIC) which include MICA and MICB proteins, and the structurally diverse unique long 16 binding proteins (UL16-binding proteins or ULBP) which include ULBPs 1 to 6 [31]. The expression of these ligands can be induced by stress such as viral infection, oxidative or thermal stress, genotoxic drugs, DNA damaging agents, tissue damage, heat shock, inflamed tissues (*e.g.* autoimmune diseases) and malignant transformation [32]. Importantly, a broad range of primary tumours express NKG2D ligands [33, 34]. Whilst the NKG2D ligand expression profile is often heterogeneous, the large majority of tumour samples express at least one ligand (and often multiple ligands) including haematological and solid tumour types, implying that the profile of ligand expression is suitable to be exploited as a cancer therapy [34]. Finally, the surface expression of NKG2D ligands can be modulated by proteolytic cleavage with the release of the soluble ectodomain [35, 36]. These shed NKG2D ligands may down-modulate NKG2D expression on NK cells and T-cells, contributing to tumour immune escape and elevated serum levels were accordingly often associated with poor prognosis [35, 36].

The breadth of ligands targeted by NKG2D provides T-cells armed with the NKR-2 receptor the potential to challenge an extensive range of tumour indications [33, 34]. Preclinical

For peer review only - http://bmjopen.bmj.com/site/about/guidelines.xhtml

studies have shown the murine equivalent of NKR-2 (chNKG2D T-cells) can mediate potent anti-tumour activity against both haematological and solid tumours [37], including highly heterogeneous tumours with less than 10% ligand-positive tumour cells (see Table 1 for a summary of these data)[38]. Critically, these pre-clinical data in multiple syngeneic tumour models indicate that anti-tumour efficacy was not dependent upon lymphodepleting pre-conditioning while the induction of a tumour specific adaptive immune response by NKR-2 therapy enabled the long-term protective effects of the approach [39]. Importantly, preclinical data suggest four different mechanisms of action of NKR-2, including *(i)* direct cytotoxicity against cancer cells, *(ii)* anti-angiogenic activity potentially through targeting of NKG2D ligands expressed upon tumour neovasculature [40], *(iii)* modulation of the immune tumour microenvironment through direct targeting of immunosuppressive myeloid-derived suppressor cells (MDSCs) and regulatory T-cells (Tregs), and recruitment of myeloid cells and activated macrophages [41, 42], together resulting in *(iv)* the observed generation of a tumour-specific adaptive immune response [41-47] (Figure 2). Preclinical data also demonstrated that NKR-2 rapidly disappeared from tissues by seven days after injection suggesting a short-term persistence of NKR-2 (without uncontrolled expansion), although being able to induce a long-lasting memory host immune response against tumour-specific antigens [39]. Finally, preclinical data demonstrated that NKR-2 remained functionally intact in the presence of soluble MICA at concentrations much higher than the physiological concentrations [48].

Expressing the NKR-2 CAR into T-cells therefore allows the NKR-2 cells to present the broad specificity characteristic of NK cells whilst exploiting the migration, expansion, long half-life and potential to generate immunological memory characteristic of T-cells. In addition to direct recognition of tumour cells, targeting of ligands expressed on the stroma of the

For peer review only - http://bmjopen.bmj.com/site/about/guidelines.xhtml

tumour microenvironment can also lead to disruption of the essential support mechanisms required for tumour cell survival and growth. Moreover, in contrast to other single or limited target CAR-T therapies, the fact that NKR-2 targets several ligands from two main families putatively decreases the probability of target loss and associated relapse after treatment. Further, the lack of important *in vivo* cell expansion and long-term survival of NKR-2, and the induction of tumour specific adaptive immunity in the absence of patient pre-conditioning, also bears a safety-advantage preventing side effects generally associated with rapid CAR-T expansion [49]. Collectively, this suggests that exploiting the targeting of NKG2D ligands by NKR-2 to fight against diverse tumour indications with a single generic construct combining two features of innate and adaptive immunity, provides potentially a new paradigm for CAR T-cell therapy that the current protocol is designed to investigate.

For peer review only - http://bmjopen.bmj.com/site/about/guidelines.xhtml

Methods and Analysis

Study objectives

The THINK (**TH**erapeutic **I**mmunotherapy with **NK**R-2) trial is an open-label Phase I study which primarily aims to assess the safety and clinical activity of the NKR-2 treatment administered three times every 2 weeks between each administration in different tumour types. The study is split into two segments each with specific study objectives. The first dose escalation segment focuses upon determining the recommended dose of NKR-2 cells for second segment of THINK. Segment 2 extends the safety study and also investigates initial clinical activity of NKR-2 across multiple tumour indications. In both segments, correlative measures of clinical activity will be examined.

Study drug

NKR-2 refers to the viable autologous $CD3^+$ cells obtained after transduction of autologous T-cells with the NKG2D-based CAR, which contains a total viable population of at least 80% $CD3^+$ cells, with at least 50% $CD3^+$ being transduced. NKR-2 will be supplied cryopreserved in bags containing a T-cell dose in accordance with the dose-level which is to be administered.

Rationale for the NKR-2 dose and regimen

NKR-2 has been used in the single infusion dose escalation clinical study CM-CS1 (NCT02203825) which evaluated the safety and clinical activity NKR-2 administration without prior lymphodepleting conditioning in patients with two distinct haematologic malignancies: Acute Myeloid Leukemia (AML)/ Higher Risk Myelodysplastic Syndrome (MDS) refractory anemia with excess blasts, that are not in remission and for which standard therapy options are not available, and relapsed or refractory progressive Multiple Myeloma

For peer review only

(MM) after several lines of standard therapy. The trial has been successfully completed with no observed dose limiting toxicities at the highest dose infused ($3x10^7$ total cells) and initial signs of clinical benefit [50].

In preclinical models, the pharmacologically effective dose (PED) of the murine NKR-2 ranged from $1x10^6$ cells to $7.5x10^6$ cells per dose (intravenous (i.v.) single injection). Although a significant anti-tumour activity was obtained with a single injection of murine NKR-2, 100% overall survival and complete recovery was obtained following repeated three sequential doses of $5x10^6$ cells/mouse without any adverse effect [39, 41, 45, 47]. Moreover, a treatment regimen with two weeks' interval between injections was far more successful in terms of activity as compared to a weekly interval demonstrating that successful treatment of established tumours did not require the administration of more NKR-2 [41]. The maximum tolerated dose (MTD) after a single i.v. injection was determined to be $1x10^7$ cells, and repeated administration of this dosage to healthy and tumour-bearing mice did not produce any major toxicities [51].

Following these values of PED and MTD, the no-observed effect level (NOEL) was defined at $5x10^6$ cells/injection per mouse and were identical whatever the tumour type model (haematological or solid tumour). Using the allometry scaling exponent of 0.75, as defined by FDA guidelines (USFDA, 2005), human-equivalent PED and MTD were therefore estimated at 0.43 - $3.2x10^9$ and $4.3x10^9$ cells/injection, respectively. Together these observations of high tolerability in initial single dose phase I clinical trial coupled with pre-clinical efficacy experimentation strongly support the concept of a dose escalation segment of $3x10^8$ to $3x10^9$ NKR-2 per infusion given three times each separated by a 14-day interval.

Patient population

For peer review only - http://bmjopen.bmj.com/site/about/guidelines.xhtml

Refractory or relapsing patients with metastatic or locally advanced colorectal cancer (CRC), urothelial carcinoma, triple-negative breast cancer (TNBC), pancreatic cancer, recurrent epithelial ovarian and fallopian tube carcinoma, acute myeloid leukemia AML/MDS and MM, which failed from standard treatments, will be recruited into this protocol (the key inclusion and exclusion criteria are presented in Table 2). The study will enrol patients in different countries in Europe and in USA with a dose escalation segment open to a restricted network.

The tumour types targeted in this protocol were selected primarily based on their high expression of the NKG2D ligands (with a prevalence of 78-100% tumour samples expressing at least 1 ligand) [33, 34]. Furthermore, NKG2D ligand expression is a dynamic and context-dependent process, quickly varying over time in response to cytokines in the tumour microenvironment and evolving along with tumour progression, local inflammatory responses and treatments [33, 34], hence difficult to assess. Finally, even if NKG2D ligand expression is heterogeneous with respect to the exact ligands found, based on preclinical data, potential therapeutic benefit might be achieved in patients with highly heterogeneous tumours [38] or even tumours considered as "negative" for NKG2D ligands because of a NKG2D ligand positive tumour microenvironment and/or neovasculature and/or activation of host immune system [41-47]. Thus, no eligibility criteria was based upon the individual expression profile of NKG2D ligands and no diagnostic test for ligand expression used for recruitment into this study. Correlative studies, however, will be carried out during the trial and will attempt to investigate the expression profile of patient tumours with clinical response.

THINK study design

For peer review only - http://bmjopen.bmj.com/site/about/guidelines.xhtml

THINK Phase I study will contain two consecutive segments: a dose escalation and an expansion segment (Figure 3).

The **dose escalation** design will include 3 dose-levels of NKR-2. The assumption is made that the safety profile of the treatment could be different in haematological versus solid tumour types. Therefore, it will be assessed separately in two different arms, one in solid tumours (CRC, urothelial carcinoma, TNBC, pancreatic cancer or epithelial ovarian and fallopian tube carcinoma) and one in haematological tumours (AML/MDS or MM). Each arm will use a 3+3 design to determine the recommended dose (RecD) of the NKR-2 treatment for the corresponding cohorts in the expansion segment.

The RecD will be further evaluated in the **expansion segment** of the study to assess separately the safety profile and initial clinical activity of the NKR-2 treatment in 7 cohorts of patients with the distinct tumour types (up to 14 patients per tumour type). The expansion segment in haematological and solid tumours will be initiated as soon as their respective dose escalation arm defines the RecD. During this segment, the RecD can still be adapted according to predefined safety rules in any specific tumour type.

18 patients in the dose escalation segment and up to 86 additional patients in the expansion segment of the study are expected to be enroled in the study.

Treatment regimen

Three NKR-2 dose-levels will be evaluated during this study, *i.e.* $3x10^8$, $1x10^9$ and $3x10^9$ NKR-2 for each injection (adjusted to $4.6x10^6$, $1.5x10^7$ and $4.6x10^7$ NKR-2/kg, respectively for patients with body weight below or equal to 65 kg) with a schedule of administration of

For peer review only - http://bmjopen.bmj.com/site/about/guidelines.xhtml

three NKR-2 doses administered with a 2-week interval per patient. No systemic lymphodepleting pre-conditioning will be performed prior to NKR-2 injections.

Trial Endpoints

The *primary endpoint* of the **dose escalation** segment will be the occurrence of dose limiting toxicities (DLTs) in all patients during the study treatment until 14 days after the last study treatment administration. *Secondary endpoints* will include the *(i)* occurrence of adverse events (AEs) and serious adverse events (SAEs) during the study treatment, *(ii)* occurrence of objective clinical response according international guidelines, *(iii)* progression-free survival (PFS) and overall survival (OS), and duration of response for patients with objective response up to two years after first NKR-2 administration.

The *Primary endpoint* of the **expansion segment** will be the objective response rate (ORR) up to two years after first NKR-2 administration. *Secondary endpoints* will include the *(i)* PFS and OS, *(ii)* duration of response for patients with objective response and *(iii)* occurrence of AEs and SAEs and DLTs during the study treatment.

The overall aim of this segment is to understand the safety profile and develop initial observations of clinical activity in a cohort of up to 14 patients that support a deeper assessment of clinical efficacy of NKR2 in larger patient cohorts within a Phase II clinical study.

Response to treatment will be assessed according to standard RECIST criteria (version 1.1) for solid tumours or international recommendations for response criteria in haematological tumours.

Study duration

For peer review only - http://bmjopen.bmj.com/site/about/guidelines.xhtml

For peer review only

For each patient, the overall study duration will be 2 years (24 months). The study consists of a screening phase, a treatment administration phase and a follow-up phase (Figure 4). The duration of the administration phase will be 8 weeks, from Visit D1 to Visit D57. The duration of the follow-up phase will be 22 months, from the end of the administration phase on D57 (Week 8) to Visit M24.

As per regulatory requirements, patients who received at least one NKR-2 treatment will need to be followed annually for a period of 15 years. Therefore, as treatment follow-up period ends at 24 months, at the end of the study period, patients are proposed to enter a **long-term safety follow-up** that will last for up to 15 years after the day of the first NKR-2 administration. The purpose of this separate study is to collect safety data for 13 additional years. The surveillance will mimic as much as possible the standard follow-up of the respective tumour type including monitoring of important safety parameters.

Enrolment rules

There is no requirement for specific recruitment of each individual tumour indications to corresponding arm within the **dose escalation phase**, but each dose-level should enrol at least two different types of cancer. In each arm, patients will be enroled at the next dose-level when all evaluable patients at the same dose-level have completed the safety period of 14 days after last NKR-2 injection. Within each dose-level, patients will be enroled as soon as the previous patient has received at least one NKR-2 administration and has completed the 14-day safety period after the 1st injection.

Enrolment in the **expansion segment** of the study will be performed in 3 steps, with a statistical analysis at the end of each step (see next section). Within each step, all patients can theoretically be enroled at the same time.

For peer review only - http://bmjopen.bmj.com/site/about/guidelines.xhtml

Conduct of analysis

The analysis of clinical activity will be performed on the *total treated population i.e.* all patients which received at least one NKR-2 treatment (in case of patient's withdrawal prior the end of the administration phase). Sensitivity analyses related to the clinical activity endpoints will be performed on the *according to protocol* population which includes all evaluable patients who have complied with all the procedures defined in the protocol up to concluding visit.

All statistical analyses will be performed using SAS and/or StatXact software. The results will be reported per dose-level/tumour type cohort and overall.

In the dose-escalation segment, safety data will be monitored closely on an ongoing basis. The main analysis (leading to the selection of the RecD) will be conducted separately in each arm once all patients in the last dose-level have received the 3 NKR-2 administrations and have completed the safety period (D43). All data related to the primary endpoints of the study will be analyzed. The final analysis will be performed once all patients have attended the concluding visit (M24) or have been withdrawn from the study.

The expansion segment of the study will proceed in 3 steps, with a statistical analysis at the end of each step (Figure 3). The 6 patients from the dose escalation segment of the study who were treated at the RecD will be included in these expansion analyses. These steps aim to stop the study for futility in specific cohorts or specific tumour types in case of no (sufficient) responders in the corresponding cohorts at the time of analysis.

An interim analysis for futility will be performed on the first 14 patients over all 5 cohorts of patients with solid tumour types and on the first 6 patients over the two cohorts of patients

For peer review only

with haematological tumour types who have either completed the administration phase (D57) or have withdrawn from the study.

The second step consists in cohort futility analyses after the first 7 cancer patients in each tumour type have either completed the administration phase (D57) or have withdrawn from the study. At this step, an evaluation of the safety of the NKR-2 treatment in each cohort will also be performed on the first 6 patients who completed the 6-week treatment period (D43) or had a DLT. This will allow to adapt the RecD in any specific cohort (tumour type) according to both safety rules and futility analysis. Indeed, if, and as soon as, 2 or more DLTs have been reported within a specific cohort, and the criterion for futility was not reached, the next patients enrolled in this cohort will be treated at the next lower dose-level. In contrast, if no DLT has been reported within a specific cohort, and the criterion for futility was not reached at step 2, the next patients enrolled will be treated with the next higher dose-level.

Finally, the final evaluation of the objective clinical response rate will be evaluated once all the first 14 cancer patients in each cohort have either completed their administration period (D57) or have withdrawn from study due to disease progression or DLT.

Correlative studies

Peripheral blood samples, bone marrow aspirates from patients with haematologic malignancies, as well as tumour biopsies from patients with solid malignancies, will be collected at regular time-points during the study (see Figure 4) and used to determine NKR-2 persistence post-infusion (using a viral copy number test) and assess the presence of replication competent retrovirus (RCR). Systemic cytokine levels in peripheral blood and levels of tumour-related biomarkers (in the serum, bone-marrow or urine samples) will be also assessed to determine whether correlates of NKR-2 activity can be identified. Evaluation

For peer review only - http://bmjopen.bmj.com/site/about/guidelines.xhtml

and characterization of the NKR-2 mode of action, involving the characterization of the pre-existing and post/during-therapy humoral and cellular immune responses will be done on blood samples collected at baseline and post-NKR-2 infusion. Finally, the correlation with the tumour expression of NKG2D ligand in tumour tissues or cells (type and level) and NKR-2 activity, the characterization of the tumour microenvironment (TME), the NKR-2 infiltration and the modulation of the local tumour environment of this new type of T-cell therapy will be evaluated.

Toxicity Management

As previously mentioned, the use of CAR T-cells is limited by potentially severe toxicities including cytokine release syndrome (CRS), neurotoxicity and tumour lysis syndrome (TLS) (on-target on-tumour toxicities), on-target off-tumour toxicity, clonality and insertional oncogenesis, vector persistence and immunogenicity. While these events may have been seen in relation with other CAR-T therapies, and are provided for information, there are currently no events considered as expected for NKR-2 therapy (which can be different from other CAR-T studies based on the unique mode of action) and all Serious Adverse Reactions (SARs) will be considered as suspected unexpected SARs (SUSARs).

The management of toxicities related to CAR-T therapies was described elsewhere [11, 12, 52] but is evolving with everyday new information available from ongoing studies. Sites will be kept informed all along the study for any new therapeutic options modifications or standardization between the different sites.

Briefly, CRS toxicity is currently of highest concern in the field. Tocilizumab (anti IL-6) will be used as the first-line agent in the treatment of CRS. If the patient's condition does not improve or stabilize within 24 hours of the tocilizumab dose, administration of a second dose

For peer review only - http://bmjopen.bmj.com/site/about/guidelines.xhtml

of tocilizumab and/or a second immunosuppressive agent (such as corticosteroids) will be considered, based on the CRS revised grading system created by Lee et al. [53] to identifying severity and implementation of optimal care strategies for CRS.

TLS resulting in renal insufficiency, rapidly rising uric acid level, or evidence of organ dysfunction will be managed with fluids and rasburicase as needed and as determined by the treating physicians.

Further, as NKG2D ligands may be upregulated upon stress signals, expression of ligands on non-cancer cells can be expected in tissues that are actively infected or inflamed or experienced cell stress of other etiology. Infections could therefore invoke a significant inflammatory response by NKR-2. Therefore, any inflammation, infection, concurrent or previous treatment with anti-cancer therapies, will be carefully registered and any Grade 2 or higher autoimmune toxicity will be closely followed to detect any evidence of on-target off-tumour toxicity. Evidence of acute infection, as well as fever of 38°C/100°F or above, will be therefore considered as exclusion criteria at screening or postponement criteria when observed during the treatment phase. In the latter, all study procedures and treatment administration will be resumed where it was left as soon as the patient's condition allows (within 4 weeks after the scheduled treatment date) or at the investigator's discretion after Steering Committee and Sponsor approvals.

Since NKR-2 construct consists entirely of human sequences, it is not expected to result in immunogenicity sometimes observed with other CAR-Ts using scFv from murine origin (see introduction), and therefore multiple NKR-2 injections are allowed without the anticipated risk of humoral immune responses.

For peer review only - http://bmjopen.bmj.com/site/about/guidelines.xhtml

Uncontrolled expansion and proliferation of the transformed cells increases the risks for onset of cytokine release syndrome. However, in contrast to more classical CAR-T therapies, NKR-2 demonstrates neither significant *in vivo* expansion nor long-term persistence [39]. However, this is the first trial with NKR-2 administrated as multiple doses. The persistence of NKR-2 will be therefore carefully monitored post-infusion as mandatory exploratory research.

Finally, following FDA recommendation for Retroviral and Lentiviral Vector Based Gene Therapy Products, the patient monitoring schedule will also include analysis of the replication-competent retrovirus (RCR) of patient samples up to 15 years after CAR-T injections.

For peer review only - http://bmjopen.bmj.com/site/about/guidelines.xhtml

For peer review only

Discussion

The THINK trial aims primarily to determine the safety and clinical activity of NKR-2 cell therapy, without pre-conditioning chemotherapy, in patients with advanced cancer. The dose escalation phase of the trial is designed to determine the recommended dose in haematological and solid tumours, which then is used in the expansion phase to assess clinical responses separately in seven different indications. Due to the multi-targeting specificity of NKR-2, the dose escalation and subsequent expansion segments are designed to facilitate a rapid assessment of NKR-2 activity across a range of tumours. This is advantageous since the assessment of safety and any evidence of clinical activity will be suitably evaluated across cancer types using a consistently produced cell product thereby avoiding issues that arise in multiple single tumour indication trials testing a single T-cell product. Interestingly, the number of ligands targeted by NKR-2 (8 ligands from 2 main families), combined with their stress-induced tissue expression, also limit the potential risk of clonal selection or antigen loss after injection, which has been observed for classical CAR-T constructs targeting a single antigen. This is expected to translate into limiting the risk for potential relapse.

For patients, the lack of pre-conditioning chemotherapy removes the need for an in-patient stay during the transient period of lymphodepletion translating to a likely improved patient experience of the trial. Additionally, the absence of pre-conditioning also will reduce the potential toxicity of this treatment and facilitate providing a therapeutic solution to a larger patient population, including, notably, the elderly population.

The challenge facing classical CAR-T therapies in the human patient with advanced cancer (beyond B-cell malignancies) is major and, in the solid tumour situation, requires the cells to traffic to sites of tumour, overcome the highly suppressive tumour microenvironment and

For peer review only

initiate direct tumour cell killing to drive a sustained anti-tumour response [23-25]. Based on the different modes of action of NKR-2 observed in preclinical experiments on various syngeneic tumour models (lymphoma, myeloma, ovarian cancer and melanoma), acting not only directly on tumour cells but also on tumour microenvironment and neovasculature, the THINK trial offers a real opportunity as compared with classical CAR-Ts, especially within the solid tumour type field, where therapeutic options are limited.

The long-term efficacy and generation of a tumour specific adaptive immune response [41-47] despite of the short-term persistence and limited expansion of NKR-2 after infusion [39], as observed in preclinical models, also indicate another divergence from the standard CAR T-cell approach where massive *in vivo* expansion appears essential to achieve sufficient CAR T-cells to drive a direct anti-tumour response. From a different perspective, it was shown, for classical CAR-Ts, that long-term persistence and expansion of adoptively transferred T-cells – highly variable between patients – can increase and prolong both therapeutic and deleterious effects, like CRS. Therefore, the lack of potential long-term survival of NKR-2 therefore bears a safety-advantage as compared to other CAR-T therapies which often require strategies like embedded suicide systems or chemotherapy to eradicate the CAR T-cells [54, 55]. The multiple infusion strategy, allowing complete anti-tumour efficacy in preclinical models with NKR-2 [39, 41, 45, 47] was also found to increase activity and infiltration into tumours of other short-term persistent CAR T-cells (mesothelin-specific RNA CAR-T cells) [56], overcome the potential tumour-induced T-cell dysfunction upon interaction of CAR-T cells with the immunosuppressive tumour microenvironment [23], and is well tolerated in humans [57].

For peer review only - http://bmjopen.bmj.com/site/about/guidelines.xhtml

The multiple infusion strategy, absence of pre-conditioning chemotherapy, diversity of putative mechanisms of action, as well as the diversity of tumour types that can potentially targeted, therefore stand the NKR-2 CAR T-cell approach apart from the current 'gold standard' CAR T-cell therapy paradigm.

For peer review only

For peer review only - http://bmjopen.bmj.com/site/about/guidelines.xhtml

Ethics and dissemination

The THINK trial (A Dose Escalation Phase I Study to Assess the Safety and Clinical Activity of Multiple Cancer Indications) is currently recruiting participants in Europe and USA. Ethical approval has been obtained at all sites open for recruitment. The trial has been approved by ClinicalTrials.gov (Trial registration ID NCT03018405) and by EU Clinical Trials Register (EudraCT number: 2016-003312-12). Written informed consent will be taken from all participants. Results of this study will be disseminated through presentation at international scientific conferences and reported in peer-reviewed scientific journals.

For peer review only

For peer review only - http://bmjopen.bmj.com/site/about/guidelines.xhtml

Abbreviations

AE: adverse event; AML: acute myeloid leukemia; CAR: chimeric antigen receptor; chNKG2D: NKG2D-based CAR; CR: complete response; CRC: colorectal cancer; CRS: cytokine release syndrome; DAP10: DNAX-activating protein of 10 kDa; DLT: dose-limiting toxicity; MDS: myelodysplastic syndrome; MDSC: myeloid-derived suppressor cell; MHC: major histocompatibility complex; MICA/B: MHC class I chain related proteins A/B; MM: multiple myeloma; MTD: maximum tolerated dose; NK: natural killer; NKG2D: natural killer group 2D; NKR-2: human NKG2D receptor-based CAR T-cells; NOEL: no observed effect level; ORR: overall response rate; OS: overall survival; PBMC: peripheral blood mononuclear cell; PED: pharmacologically effective dose; PFS: progression-free survival; PR: partial response; RAEB: refractory anemia with excess blasts; RCR: replication competent retrovirus; RecD: recommended dose for expansion segment; SAR: serious adverse reactions; SAE: serious adverse event; scFv: single-chain variable fragment; sMICA/B or sULBP1-6: serum level of MICA/B or ULBP1-6; SD: stable disease; SUSAR: suspected unexpected SAR; THINK: THerapeutic Immunotherapy with NKR-2; TLS: tumour lysis syndrome; TME: tumour microenvironment; TNBC: triple-negative breast cancer; Treg: regulatory T-cell; ULBP1-6: unique long 16 binding protein 1-6.

For peer review only

For peer review only - http://bmjopen.bmj.com/site/about/guidelines.xhtml

References

1. Fesnak AD, June CH, Levine BL. Engineered T cells: the promise and challenges of cancer immunotherapy. *Nat Rev Cancer* 2016;16(9):566-81.
2. Jackson HJ, Rafiq S, Brentjens RJ. Driving CAR T-cells forward. *Nat Rev Clin Oncol* 2016;13(6):370-83.
3. Dai H, Wang Y, Lu X, et al. Chimeric Antigen Receptors Modified T-Cells for Cancer Therapy. *J Natl Cancer Inst* 2016;108(7).
4. Gross G, Eshhar Z. Therapeutic Potential of T Cell Chimeric Antigen Receptors (CARs) in Cancer Treatment: Counteracting Off-Tumor Toxicities for Safe CAR T Cell Therapy. *Annu Rev Pharmacol Toxicol* 2016;56:59-83.
5. Pule MA, Savoldo B, Myers GD, et al. Virus-specific T cells engineered to coexpress tumor-specific receptors: persistence and antitumor activity in individuals with neuroblastoma. *Nat Med* 2008;14(11):1264-70.
6. Rosenberg SA, Yang JC, Sherry RM, et al. Durable complete responses in heavily pretreated patients with metastatic melanoma using T-cell transfer immunotherapy. *Clin Cancer Res* 2011;17(13):4550-7.
7. Maude SL, Frey N, Shaw PA, et al. Chimeric antigen receptor T cells for sustained remissions in leukemia. *N Engl J Med* 2014;371(16):1507-17.
8. Lee DW, Kochenderfer JN, Stetler-Stevenson M, et al. T cells expressing CD19 chimeric antigen receptors for acute lymphoblastic leukaemia in children and young adults: a phase 1 dose-escalation trial. *The Lancet* 2015;385(9967):517-28.
9. Grupp SA, Kalos M, Barrett D, et al. Chimeric antigen receptor-modified T cells for acute lymphoid leukemia. *N Engl J Med* 2013;368(16):1509-18.
10. Gardner R, Wu D, Cherian S, et al. Acquisition of a CD19-negative myeloid phenotype allows immune escape of MLL-rearranged B-ALL from CD19 CAR-T-cell therapy. *Blood* 2016;127(20):2406-10.
11. Bonifant CL, Jackson HJ, Brentjens RJ, et al. Toxicity and management in CAR T-cell therapy. *Mol Ther Oncolytics* 2016;3:16011.
12. Brudno JN, Kochenderfer JN. Toxicities of chimeric antigen receptor T cells: recognition and management. *Blood* 2016;127(26):3321-30.
13. Brentjens RJ, Riviere I, Park JH, et al. Safety and persistence of adoptively transferred autologous CD19-targeted T cells in patients with relapsed or chemotherapy refractory B-cell leukemias. *Blood* 2011;118(18):4817-28.
14. Kochenderfer JN, Dudley ME, Feldman SA, et al. B-cell depletion and remissions of malignancy along with cytokine-associated toxicity in a clinical trial of anti-CD19 chimeric-antigen-receptor-transduced T cells. *Blood* 2012;119(12):2709-20.
15. Kershaw MH, Westwood JA, Parker LL, et al. A phase I study on adoptive immunotherapy using gene-modified T cells for ovarian cancer. *Clin Cancer Res* 2006;12(20 Pt 1):6106-15.
16. Lamers CH, Willemsen R, van Elzakker P, et al. Immune responses to transgene and retroviral vector in patients treated with ex vivo-engineered T cells. *Blood* 2011;117(1):72-82.
17. Turtle CJ, Hanafi LA, Berger C, et al. CD19 CAR-T cells of defined CD4+:CD8+ composition in adult B cell ALL patients. *J Clin Invest* 2016;126(6):2123-38.
18. Maus MV, Haas AR, Beatty GL, et al. T cells expressing chimeric antigen receptors can cause anaphylaxis in humans. *Cancer Immunol Res* 2013;1(1):26-31.

For peer review only - http://bmjopen.bmj.com/site/about/guidelines.xhtml

19. Khalil DN, Smith EL, Brentjens RJ, et al. The future of cancer treatment: immunomodulation, CARs and combination immunotherapy. *Nat Rev Clin Oncol* 2016;13(5):273-90.

20. Lamers CH, Langeveld SC, Groot-van Ruijven CM, et al. Gene-modified T cells for adoptive immunotherapy of renal cell cancer maintain transgene-specific immune functions in vivo. *Cancer Immunol Immunother* 2007;56(12):1875-83.

21. Morgan RA, Yang JC, Kitano M, et al. Case report of a serious adverse event following the administration of T cells transduced with a chimeric antigen receptor recognizing ERBB2. *Mol Ther* 2010;18(4):843-51.

22. Beatty GL, O'Hara M. Chimeric antigen receptor-modified T cells for the treatment of solid tumors: Defining the challenges and next steps. *Pharmacol Ther* 2016;166:30-9.

23. Moon EK, Wang LC, Dolfi DV, et al. Multifactorial T-cell hypofunction that is reversible can limit the efficacy of chimeric antigen receptor-transduced human T cells in solid tumors. *Clin Cancer Res* 2014;20(16):4262-73.

24. Newick K, Moon E, Albelda SM. Chimeric antigen receptor T-cell therapy for solid tumors. *Mol Ther Oncolytics* 2016;3:16006.

25. Abken H. Adoptive therapy with CAR redirected T cells: the challenges in targeting solid tumors. *Immunotherapy* 2015;7(5):535-44.

26. Han S, Latchoumanin O, Wu G, et al. Recent clinical trials utilizing chimeric antigen receptor T cells therapies against solid tumors. *Cancer Lett* 2017;390:188-200.

27. Beatty GL, Moon EK. Chimeric antigen receptor T cells are vulnerable to immunosuppressive mechanisms present within the tumor microenvironment. *Oncoimmunology* 2014;3(11):e970027.

28. Lanier LL. NKG2D Receptor and Its Ligands in Host Defense. *Cancer Immunol Res* 2015;3(6):575-82.

29. Garrity D, Call ME, Feng J, et al. The activating NKG2D receptor assembles in the membrane with two signaling dimers into a hexameric structure. *Proc Natl Acad Sci U S A* 2005;102(21):7641-6.

30. Gilfillan S, Ho EL, Cella M, et al. NKG2D recruits two distinct adapters to trigger NK cell activation and costimulation. *Nat Immunol* 2002;3(12):1150-5.

31. Carapito R, Bahram S. Genetics, genomics, and evolutionary biology of NKG2D ligands. *Immunol Rev* 2015;267(1):88-116.

32. Raulet DH, Gasser S, Gowen BG, et al. Regulation of ligands for the NKG2D activating receptor. *Annu Rev Immunol* 2013;31:413-41.

33. Le Bert N, Gasser S. Advances in NKG2D ligand recognition and responses by NK cells. *Immunol Cell Biol* 2014;92(3):230-6.

34. Spear P, Wu M-R, Sentman M-L, et al. NKG2D ligands as therapeutic targets. *Cancer Immunity Archive* 2013;13:8.

35. Holdenrieder S, Stieber P, Peterfi A, et al. Soluble MICB in malignant diseases: analysis of diagnostic significance and correlation with soluble MICA. *Cancer Immunol Immunother* 2006;55(12):1584-9.

36. Salih HR, Rammensee HG, Steinle A. Cutting Edge: Down-Regulation of MICA on Human Tumors by Proteolytic Shedding. *J Immunol* 2002;169(8):4098-102.

37. Sentman CL, Meehan KR. NKG2D CARs as cell therapy for cancer. *Cancer J* 2014;20(2):156-9.

38. Spear P, Barber A, Rynda-Apple A, et al. NKG2D CAR T-cell therapy inhibits the growth of NKG2D ligand heterogeneous tumors. *Immunol Cell Biol* 2013;91(6):435-40.

For peer review only - http://bmjopen.bmj.com/site/about/guidelines.xhtml

39. Barber A, Meehan KR, Sentman CL. Treatment of multiple myeloma with adoptively transferred chimeric NKG2D receptor-expressing T cells. *Gene Ther* 2011;18(5):509-16.
40. Zhang T, Sentman CL. Mouse tumor vasculature expresses NKG2D ligands and can be targeted by chimeric NKG2D-modified T cells. *J Immunol* 2013;190(5):2455-63.
41. Barber A, Rynda A, Sentman CL. Chimeric NKG2D expressing T cells eliminate immunosuppression and activate immunity within the ovarian tumor microenvironment. *J Immunol* 2009;183(11):6939-47.
42. Spear P, Barber A, Rynda-Apple A, et al. Chimeric antigen receptor T cells shape myeloid cell function within the tumor microenvironment through IFN-gamma and GM-CSF. *J Immunol* 2012;188(12):6389-98.
43. Barber A, Sentman CL. Chimeric NKG2D T cells require both T cell- and host-derived cytokine secretion and perforin expression to increase tumor antigen presentation and systemic immunity. *J Immunol* 2009;183(4):2365-72.
44. Barber A, Sentman CL. NKG2D receptor regulates human effector T-cell cytokine production. *Blood* 2011;117(24):6571-81.
45. Barber A, Zhang T, Sentman CL. Immunotherapy with Chimeric NKG2D Receptors Leads to Long-Term Tumor-Free Survival and Development of Host Antitumor Immunity in Murine Ovarian Cancer. *J Immunol* 2008;180:72-78.
46. Spear P, Barber A, Sentman CL. Collaboration of chimeric antigen receptor (CAR)-expressing T cells and host T cells for optimal elimination of established ovarian tumors. *Oncoimmunology* 2013;2(4):e23564.
47. Zhang T, Barber A, Sentman CL. Chimeric NKG2D modified T cells inhibit systemic T-cell lymphoma growth in a manner involving multiple cytokines and cytotoxic pathways. *Cancer Res* 2007;67(22):11029-36.
48. Zhang T, Barber A, Sentman CL. Generation of antitumor responses by genetic modification of primary human T cells with a chimeric NKG2D receptor. *Cancer Res* 2006;66(11):5927-33.
49. Tey SK. Adoptive T-cell therapy: adverse events and safety switches. *Clin Transl Immunology* 2014;3(6):e17.
50. A first-in-human phase I trial of NKG2D chimeric antigen receptor-T cells in AML/MDS and multiple myeloma. 2016 ASCO Annual Meeting; 2016. J Clin Oncol.
51. Sentman ML, Murad JM, Cook WJ, et al. Mechanisms of Acute Toxicity in NKG2D Chimeric Antigen Receptor T Cell-Treated Mice. *J Immunol* 2016;197(12):4674-85.
52. Davila ML, Riviere I, Wang X, et al. Efficacy and toxicity management of 19-28z CAR T cell therapy in B cell acute lymphoblastic leukemia. *Sci Transl Med* 2014;6(224):224ra25.
53. Lee DW, Gardner R, Porter DL, et al. Current concepts in the diagnosis and management of cytokine release syndrome. *Blood* 2014;124(2):188-95.
54. Ertl HC, Zaia J, Rosenberg SA, et al. Considerations for the clinical application of chimeric antigen receptor T cells: observations from a recombinant DNA Advisory Committee Symposium held June 15, 2010. *Cancer Res* 2011;71(9):3175-81.
55. Minagawa K, Zhou X, Mineishi S, et al. Seatbelts in CAR therapy: How Safe Are CARS? *Pharmaceuticals (Basel)* 2015;8(2):230-49.
56. Barrett DM, Liu X, Jiang S, et al. Regimen-specific effects of RNA-modified chimeric antigen receptor T cells in mice with advanced leukemia. *Hum Gene Ther* 2013;24(8):717-27.

For peer review only - http://bmjopen.bmj.com/site/about/guidelines.xhtml

57. Beatty GL, Haas AR, Maus MV, et al. Mesothelin-specific chimeric antigen receptor mRNA-engineered T cells induce anti-tumor activity in solid malignancies. *Cancer Immunol Res* 2014;2(2):112-20.
58. Zhang T, Lemoi BA, Sentman CL. Chimeric NK-receptor-bearing T cells mediate antitumor immunotherapy. *Blood* 2005;106(5):1544-51.
59. Barber A, Zhang T, DeMars LR, et al. Chimeric NKG2D receptor-bearing T cells as immunotherapy for ovarian cancer. *Cancer Res* 2007;67(10):5003-8.
60. Barber A, Zhang T, Megli CJ, et al. Chimeric NKG2D receptor-expressing T cells as an immunotherapy for multiple myeloma. *Exp Hematol* 2008;36(10):1318-28.

For peer review only

For peer review only - http://bmjopen.bmj.com/site/about/guidelines.xhtml

Footnotes

Acknowledgements

The delivery of successful clinical trials is primarily due to the excellence of the clinical partners with whom Celyad collaborates. The authors wish to thank all investigators in US and EU and all clinical managers for their valuable support and guidance. Finally, we thank all the patients and their families for participating to the study and for encouraging us to do this research.

Authors' contributions

CL, DG and FL conceived the study design, developed essential study documents and drafted the manuscript. BV, DG and FL participate in the coordination of the study. FP participated in the study design and the statistical review. AH, PA, AA, EVDN, GC, JPM, JB, DS, TK and KO participate in the management, will treat patients and conduct the clinical trial. MD gave support and guidance during the study design conception. All authors read, reviewed and approved the final manuscript.

Competing interests

CL, BV, DG and FL are employed by Celyad SA. JB and PA declare no disclosures. JPM has advisory roles with Merck Sharp and Dohme (uncompensated), Innate Pharma, Debio, Boehringer-Ingelheim, Nanobiotix.

Data sharing

Access to the dataset will only be considered when the primary results of the study have been published.

For peer review only - http://bmjopen.bmj.com/site/about/guidelines.xhtml

Figure legends

Figure 1: The NKR-2 construct. NKR-2 design contains the full-length human NKG2D receptor linked to the signalling domain of CD3ζ that provides primary signalling (signal 1) to activate T-cells upon ligand binding. The naturally expressed adaptor molecule DAP10 provides secondary signalling (signal 2) which allows NKR-2 to work as a second-generation CAR-T.

Figure 2: NKR-2 different mechanisms of action. Apart from direct cytotoxicity against cancer cells, NKR-2 mode of action also involves reduction of blood vessel density indicative of an anti-angiogenic activity, modulation of the immunosuppressive tumour microenvironment and induction of a long-term anti-tumour specific memory immune response.

Figure 3: Overview of the study design.

Figure 4: Overview of study phases. NKR-2 will be infused on Day 1, 15 and 29. Tumour assessment will be done by tumour imaging and blood sampling for solid tumours and tumour imaging, blood, urine and bone marrow sampling for haematological tumours. D = day, M = Month, BM = bone marrow.

For peer review only

For peer review only - http://bmjopen.bmj.com/site/about/guidelines.xhtml

Tables

Table 1: Preclinical studies with NKR-2

	Targeted model	Publications	Main findings
In vitro experiments			
Murine equivalent of NKR-2	Lymphoma<br>Myeloma<br>Ovarian cancer<br>Melanoma	[47, 58]<br>[39]<br>[45, 46, 59]<br>[40]	NKR-2 induces IFN-γ expression, cytokine release upon co-culture with tumour cell lines and trigger specific cytotoxicity against several tumour types
Human NKR-2	Myeloma<br>Tumour cell lines	[60]<br>[48]	NKR-2 induces IFN-γ expression, cytokine release upon co-culture with tumour cell lines and trigger specific cytotoxicity against several tumour types
In vivo experiments			
Murine equivalent of NKR-2	Lymphoma-bearing C57BL/6 mice	[38, 47, 58]	Efficacy of single i.v. and s.c. administration of NKR-2 (1-7.5x10^6 cells/injection) and resistance to a re-challenge with same tumour type
		[38]	Efficacy against heterogeneous tumours
		[47]	Efficacy of multiple i.v. administration of NKR-2 (5x10^6 cells/injection)
		[51]	Single and multiple i.v. injections of NKR-2 (5x10^6 – 2x10^7 cells/injection) are not toxic at doses < 2x10^7 NKR-2 per injection.
	Multiple myeloma-bearing C57BL/ KaLwRij mice	[39]	Efficacy of single and multiple i.v. administration of NKR-2 (5x10^6 cells/injection) and resistance to a re-challenge with same tumour type<br>Effect of lymphodepletion<br>Host memory immune response<br>Low persistence of NKR-2 in the body after administration
	Ovarian tumour-bearing C57BL/6 mice	[41-43, 45, 46, 59]	Efficacy of single i.p. administration of NKR-2 (5x10^6 cells/injection) and resistance to a re-challenge with same tumour type
		[41, 42]	Efficacy against established tumours
		[42, 43, 45, 46]	Effect on tumour microenvironment<br>Host memory immune response
		[38, 41, 45, 46]	Efficacy of multiple i.p. administration of NKR-2 (5x10^6 cells/injection)
		[38]	Efficacy against heterogeneous tumours
	Melanoma-bearing C57BL/6 mice	[40]	Efficacy of multiple i.t. administration of NKR-2 (2x10^6 cells/injection)<br>Effect on tumour vasculature
	Healthy C57BL/6 mice	[51]	Single and multiple i.v. injections of NKR-2 (5x10^6 – 2x10^7 cells/injection) are not toxic at doses < 2x10^7 NKR-2 per injection.

For peer review only

For peer review only - http://bmjopen.bmj.com/site/about/guidelines.xhtml

1 **Table 2: Key eligibility criteria**

Main inclusion criteria:

- Men or women ≥ 18 years old at the time of signing the ICF,
- Patient with specific cancer indications (see below),
- Disease must be measurable according to the corresponding guidelines,
- ECOG performance status of 0 or 1 or 2 based on peripheral neuropathy from prior therapies,
- Patient with adequate bone marrow reserve, hepatic and renal functions,
- Left ventricular ejection fraction of > 40%
- For patients with solid tumour type, the patient must agree to have a tumour biopsy at baseline.

Main exclusion criteria:

- Patient with a tumour metastasis in the central nervous system,
- Patients who have received another cancer therapy within 2 weeks before the planned day for the apheresis,
- Patients who receive or are planned to receive any other investigational product within the 3 weeks before the planned day for the first NKR-2 administration,
- Patients who are planned to receive concurrent growth factor, systemic steroid or other immunosuppressive therapy or cytotoxic agent,
- Patients who underwent major surgery within 4 weeks before the planned day for the first NKR-2 administration.

Disease-specific inclusion criteria

Patients must have either:

- A documented metastatic or locally advanced colorectal adenocarcinoma and having received, being intolerant, or having refused at least 2 prior standard cancer therapy regimens as part of their primary treatment regimen or part of their treatment for management of recurrent/persistent disease,
- A documented recurrent epithelial ovarian cancer or fallopian tube carcinoma and having received or refused at least 2 prior standard cancer chemotherapy regimens as part of their primary treatment regimen or part of their treatment for management of recurrent/persistent disease,
- An inoperable locally advanced or metastatic urothelial carcinoma that have progressed after previous platinum-based chemotherapy,
- A metastatic or locally advanced triple-negative breast cancer (TNBC) and having received at least 1 prior cancer therapy regimen as part of their treatment for management of recurrent/persistent disease,
- A metastatic or locally advanced pancreatic ductal adenocarcinoma and having received, being intolerant or having refused at maximum 2 prior lines of standard cancer chemotherapy regimens,
- Relapsed or refractory AML (*i.e.* ≥ 5% blasts in bone marrow or in peripheral blood) after one prior therapy or patients with myelodysplastic syndrome (*i.e.* ≥ 5% blasts in bone marrow or ≥ 2% blasts in peripheral blood) or MDS with TP53 mutation who failed from prior treatments,
- Relapsing or refractory/relapsing MM with a minimum of 1 prior line of systemic therapy

2

For peer review only - http://bmjopen.bmj.com/site/about/guidelines.xhtml

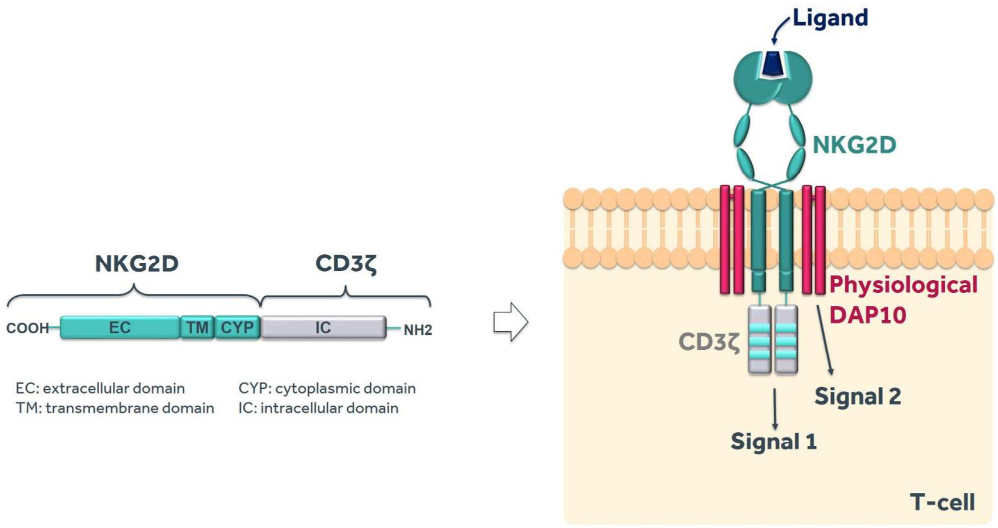

Figure 1: The NKR-2 construct. NKR-2 design contains the full-length human NKG2D receptor linked to the signalling domain of CD3ζ that provides primary signalling (signal 1) to activate T-cells upon ligand binding. The naturally expressed adaptor molecule DAP10 provides secondary signalling (signal 2) which allows NKR-2 to work as a second-generation CAR-T.

609x328mm (96 x 96 DPI)

For peer review only

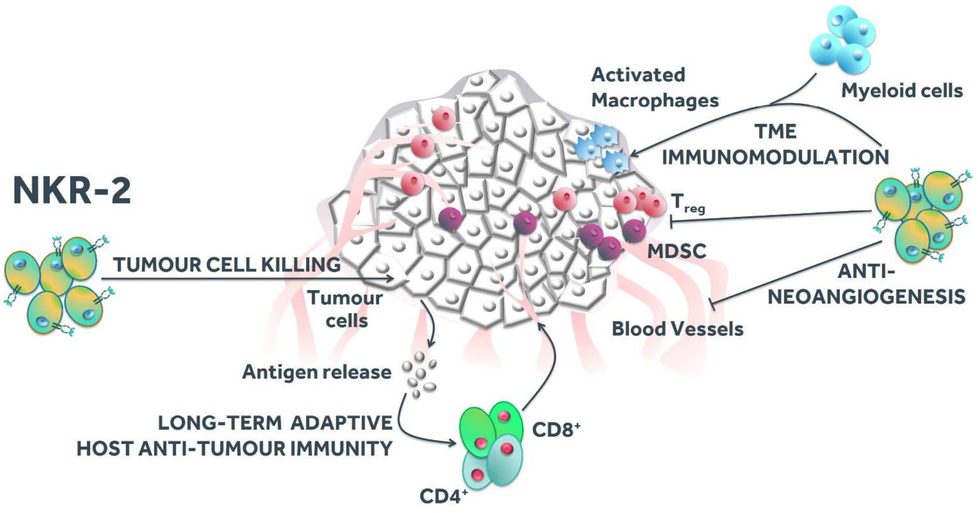

Figure 2: NKR-2 different mechanisms of action. Apart from direct cytotoxicity against cancer cells, NKR-2 mode of action also involves reduction of blood vessel density indicative of an anti-angiogenic activity, modulation of the immunosuppressive tumour microenvironment and induction of a long-term anti-tumour specific memory immune response.

720x373mm (96 x 96 DPI)

eview only

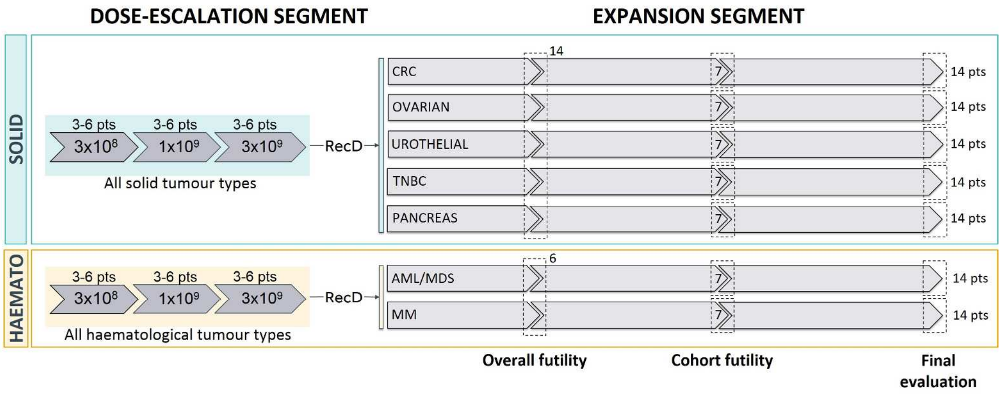

Figure 3: Overview of the study design.

758x308mm (96 x 96 DPI)

For peer review only

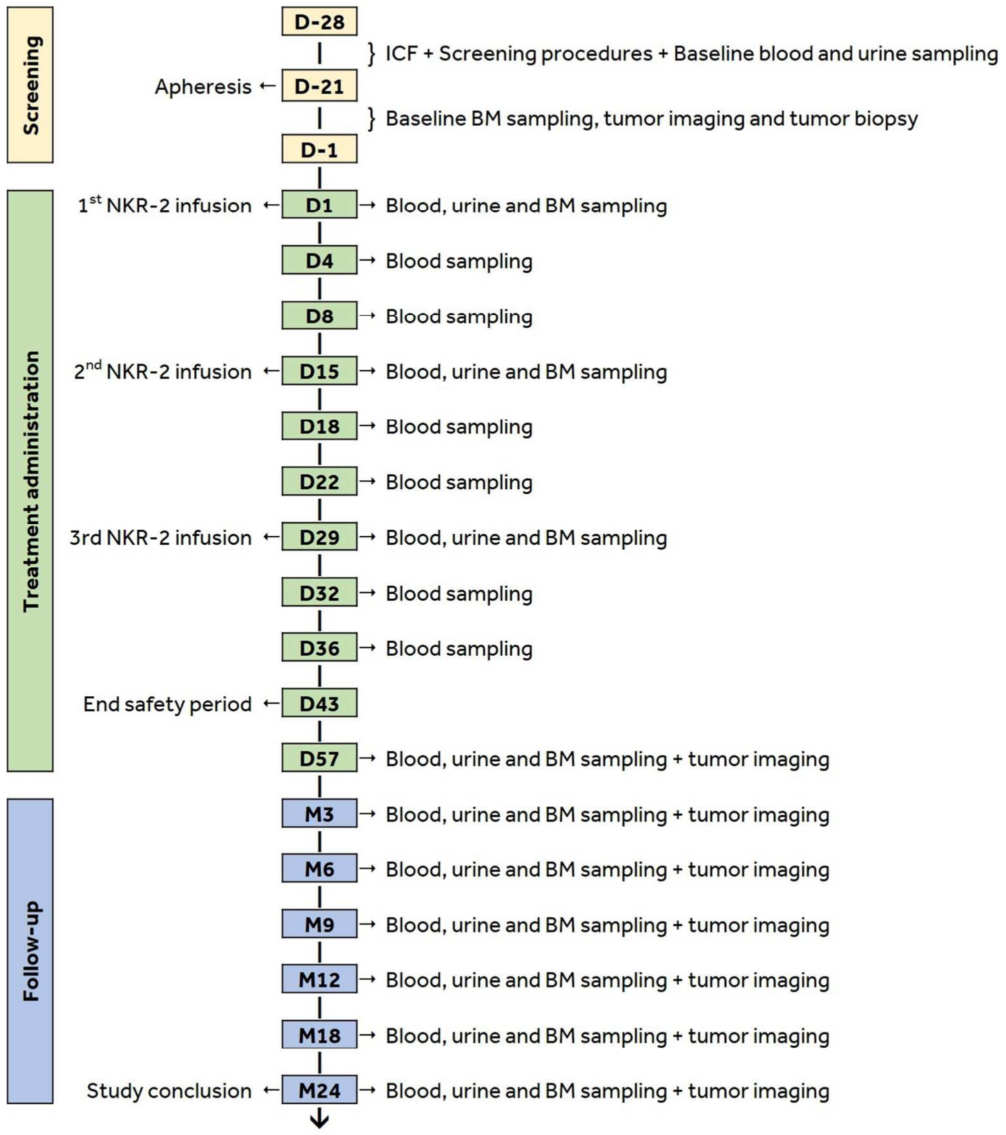

Figure 4: Overview of study phases. NKR-2 will be infused on Day 1, 15 and 29. Tumour assessment will be done by tumour imaging and blood sampling for solid tumours and tumour imaging, blood, urine and bone marrow sampling for haematological tumours. D = day, M = Month, BM = bone marrow.

392x450mm (96 x 96 DPI)

BMJ Open

THINK: A multinational open-label Phase I study to assess the safety and clinical activity of multiple administrations of NKR-2 in patients with different metastatic tumour types

Journal:	*BMJ Open*
Manuscript ID	bmjopen-2017-017075.R1
Article Type:	Protocol
Date Submitted by the Author:	10-Aug-2017

Complete List of Authors:	Lonez, Caroline; Celyad SA,<br>Verma, Bikash; Celyad SA<br>Hendlisz, Alain; Institut Jules Bordet, Medical Oncology Department-GastroIntestinal Unit<br>Aftimos, Philippe; Institut Jules Bordet, Medical Oncology<br>Awada, Ahmad; Institut Jules Bordet, Medical Oncology<br>Van Den Neste, Eric; Universite Catholique de Louvain La Faculte de Medecine<br>Catala, Gaetan; Universite Catholique de Louvain La Faculte de Medecine<br>Machiels, Jean-Pascal; Universite Catholique de Louvain La Faculte de Medecine<br>Piette, Fanny; IDDI SA<br>Brayer, Jason; H Lee Moffitt Cancer Center and Research Institute<br>Sallman, David; H Lee Moffitt Cancer Center and Research Institute<br>Kerre, Tessa; Universitair Ziekenhuis Gent<br>Odunsi, Kunle; Roswell Park Cancer Institute<br>Davila, Marco; H Lee Moffitt Cancer Center and Research Institute; USF Health Morsani College of Medicine<br>Gilham, David; Celyad SA<br>Lehmann, Frédéric; Celyad SA

**Primary Subject Heading**:	Oncology
Secondary Subject Heading:	Immunology (including allergy)
Keywords:	NKG2D, chimeric antigen receptor, CAR-T, solid tumours, NKR-2, multiple injections

SCHOLARONE™ Manuscripts

For peer review only - http://bmjopen.bmj.com/site/about/guidelines.xhtml

However, single-target therapy may select for and spur escape variants by clonal selection or antigen loss. Several CD19-specific therapies indeed evidenced cases of relapse resulting from CD19 antigen loss in leukaemia cells post-CD19-specific CAR T-cell therapy [7-10]. Furthermore, these therapies are also associated with significant levels of toxicity [11, 12] including cytokine release syndrome (CRS), neurotoxicity and on-target off-tumour toxicity against normal cells (CD19-specific CAR T-cells also target normal B-cells expressing CD19, causing prolonged B-cell aplasia and hypogammaglobulinemia [13, 14]). Moreover, anaphylaxis reaction to the foreign components of the CAR (most CARs use scFv from murine origin which might potentially induce transgene-specific humoral and cellular immune responses against the murine domain [15-17]) will impact drastically the safety of multiple injection schedule [18]. The risk/benefit is clearly biased towards benefit since these patients have highly advanced cancer with little, if any, alternative effective therapeutic option. However, this toxicity does provide a level of patient selection that impacts recruitment onto current trials and the wider applicability for the general patient population.

By contrast, outside of the B-cell malignancy setting, the CAR T-cell approach has met with much reduced levels of clinical success [2]. Notably, apart from CD19, there are only a few examples of normal cell lineages that can be safely eliminated along with malignant cells that share the targeted antigen, even within haematological malignancies. Targeting an antigen on tumour cells that is also present on normal cells, on the myeloid lineage, could lead to on-target off-tumour toxicity and serious side effects related to therapy [19]. Early studies targeting solid tumours reported little evidence of clinical effect while there was some evidence of on-target, off-tumour toxicity seen using CAR targeting carbonic anhydrase-IX [16, 20] in renal cell carcinoma or Her2/neu-specific CAR-Ts in colorectal cancer [21]. Investigators are therefore still seeking for the ideal target antigen that should be highly

For peer review only

For peer review only - http://bmjopen.bmj.com/site/about/guidelines.xhtml

overexpressed on tumour cells as compared with normal cells. Whilst some recent studies have shown limited clinical effect but nowhere near to that seen with CD19 CAR T-cell therapy [3, 22], the challenges posed by solid tumours are numerous and have been extensively reviewed recently [23-26]. In brief, these include the on-target off-tumours impact related to expression of the target on normal tissues, the need for trafficking into the tumour microenvironment and tumour stroma, tumour heterogeneity and the requirement to overcome the hostile immune suppressive tumour microenvironment, potentially leading to CAR-T exhaustion, clearly requiring the further development of the basic CAR approach [27]. Several options including third and fourth generation CARs (Armoured CAR, TRUCKs), logic gated activation strategies or the combination of CAR-Ts with T-cell checkpoint inhibitors are currently under development to solve these problems.

NKR-2

NKR-2 are autologous T-cells genetically modified to express a CAR comprising a fusion of the full-length Natural Killer Group 2D (NKG2D) receptor with the CD3 signalling domain. NKG2D is a C-type, lectin-like, type II transmembrane glycoprotein activating receptor expressed in humans on Natural Killer (NK), Natural Killer T, activated $CD8^+$ T-cells and, under certain conditions, some $CD4^+$ and $\gamma\delta$+ T-cell subsets [28] and functioning to target and kill cancer or infected cells. In humans, NKG2D is expressed as a homodimer that associates with the adaptor molecule DNAX-activating protein of 10 kDa (DAP10) to form a hexameric structure [29]. The interaction with DAP10 is essential to stabilize the surface expression of NKG2D in T-cells and to mediate signal transduction and cellular activation upon ligand recognition [30]. Therefore, whilst the NKR-2 CAR format suggests a 'first generation' configuration, the NKG2D structural component, via its interaction with endogenous DAP10, engages native T-cell costimulatory signalling apparatus to providing a sufficient co-

For peer review only

For peer review only - http://bmjopen.bmj.com/site/about/guidelines.xhtml

Table 1: Preclinical studies with NKR-2

	Targeted model	Publications	Main findings
In vitro experiments			
Murine equivalent of NKR-2	Lymphoma<br>Myeloma Ovarian cancer<br>Melanoma	[37, 38]<br>[39]<br>[40-42]<br>[43]	NKR-2 induces IFN- expression, cytokine release upon co-culture with tumour cell lines and trigger specific cytotoxicity against several tumour types
Human NKR-2	Myeloma<br>Tumour cell lines	[44]<br>[45, 46]	NKR-2 induces IFN- expression, cytokine release upon co-culture with tumour cell lines and trigger specific cytotoxicity against several tumour types
In vivo experiments			
Murine equivalent of NKR-2	Lymphoma-bearing C57BL/6 mice	[37, 38, 47]	Efficacy of single i.v. and s.c. administration of NKR-2 (1-7.5x10^6 cells/injection) and resistance to a re-challenge with same tumour type
		[47]	Efficacy against heterogeneous tumours
		[38]	Efficacy of multiple i.v. administration of NKR-2 (5x10^6 cells/injection)
		[48]	Single and multiple i.v. injections of NKR-2 (5x10^6 – 2x10^7 cells/injection) are not toxic at doses < 2x10^7 NKR-2 per injection.
	Multiple myeloma-bearing C57BL/ KaLwRij mice	[39]	Efficacy of single and multiple i.v. administration of NKR-2 (5x10^6 cells/injection) and resistance to a re-challenge with same tumour type<br>Effect of lymphodepletion<br>Host memory immune response<br>Low persistence of NKR-2 in the body after administration
	Ovarian tumour-bearing C57BL/6 mice	[40-42, 49-51]	Efficacy of single i.p. administration of NKR-2 (5x10^6 cells/injection) and resistance to a re-challenge with same tumour type
		[49, 51]	Efficacy against established tumours
		[41, 42, 50, 51]	Effect on tumour microenvironment<br>Host memory immune response
		[41, 42, 47, 49]	Efficacy of multiple i.p. administration of NKR-2 (5x10^6 cells/injection)
		[47]	Efficacy against heterogeneous tumours
	Melanoma-bearing C57BL/6 mice	[43]	Efficacy of multiple i.t. administration of NKR-2 (2x10^6 cells/injection)<br>Effect on tumour vasculature
	Healthy C57BL/6 mice	[48]	Single and multiple i.v. injections of NKR-2 (5x10^6 – 2x10^7 cells/injection) are not toxic at doses < 2x10^7 NKR-2 per injection.
Human NKR-2	Pancreatic tumour xenograft-bearing NOD-scid IL2Rgamma^{null} mice	[46]	Efficacy of multiple i.p. administration of NKR-2 (5x10^6 cells/injection)

Preclinical studies have shown the murine equivalent of NKR-2 (chNKG2D T-cells) can mediate potent anti-tumour activity against both haematological and solid tumours [52],

For peer review only - http://bmjopen.bmj.com/site/about/guidelines.xhtml

For peer review only

including highly heterogeneous tumours with less than 10% ligand-positive tumour cells (see Table 1 for a summary of these data)[47].

Critically, these pre-clinical data in multiple syngeneic tumour models indicate that anti-tumour efficacy was not dependent upon lymphodepleting pre-conditioning while the induction of a tumour specific adaptive immune response by NKR-2 therapy enabled the long-term protective effects of the approach [39]. Recent data also confirmed that human NKR-2 are effective in a mouse xenograft model of human pancreatic cancer [46]. Importantly, preclinical data suggest four different mechanisms of action of NKR-2, including direct cytotoxicity against cancer cells, anti-angiogenic activity potentially through targeting of NKG2D ligands expressed upon tumour neovasculature [43], modulation of the immune tumour microenvironment through direct targeting of immunosuppressive myeloid-derived suppressor cells (MDSCs) and regulatory T-cells (Tregs), and recruitment of myeloid cells and activated macrophages [49, 51], together resulting in the observed generation of a tumour-specific adaptive immune response [38, 41, 42, 49-51, 53] (Figure 2). Preclinical data also demonstrated that NKR-2 rapidly disappeared from tissues by seven days after injection suggesting a short-term persistence of NKR-2 (without uncontrolled expansion), although being able to induce a long-lasting memory host immune response against tumour-specific antigens [39]. Finally, preclinical data demonstrated that NKR-2 remained functionally intact in the presence of soluble MICA at concentrations much higher than the physiological concentrations [45].

Expressing the NKR-2 CAR into T-cells therefore allows the NKR-2 cells to present the broad specificity characteristic of NK cells whilst exploiting the migration, expansion, long half-life and potential to generate immunological memory characteristic of T-cells. In addition

For peer review only - http://bmjopen.bmj.com/site/about/guidelines.xhtml

to direct recognition of tumour cells, targeting of ligands expressed on the stroma of the tumour microenvironment can also lead to disruption of the essential support mechanisms required for tumour cell survival and growth. Moreover, in contrast to other single or limited target CAR-T therapies, the fact that NKR-2 targets several ligands from two main families putatively decreases the probability of target loss and associated relapse after treatment. Further, the lack of important cell expansion and long-term survival of NKR-2, and the induction of tumour specific adaptive immunity in the absence of patient pre-conditioning, also bears a safety-advantage preventing side effects generally associated with rapid CAR-T expansion [54] which often require development of strategies like embedded suicide systems or use of chemotherapy to eradicate the CAR T-cells [55, 56]. Collectively, this suggests that exploiting the targeting of NKG2D ligands by NKR-2 to fight against diverse tumour indications with a single generic construct combining two features of innate and adaptive immunity, provides potentially a new paradigm for CAR T-cell therapy that the current protocol is designed to investigate.

For peer review only - http://bmjopen.bmj.com/site/about/guidelines.xhtml

Methods and Analysis

Study objectives

The THINK (**TH**erapeutic **I**mmunotherapy with **NK**R-2) trial is an open-label Phase I study which primarily aims to assess the safety and clinical activity of the NKR-2 treatment administered three times every 2 weeks between each administration in different tumour types. The study is split into two segments each with specific study objectives. The first dose escalation segment focuses upon determining the recommended dose of NKR-2 cells for second segment of THINK. Segment 2 extends the safety study and also investigates initial clinical activity of NKR-2 across multiple tumour indications. In both segments, correlative measures of clinical activity will be examined.

Study drug

NKR-2 refers to the viable autologous $CD3^+$ cells obtained after transduction of autologous T-cells with the NKG2D-based CAR, which contains a total viable population of at least 80% $CD3^+$ cells, with at least 50% $CD3^+$ being transduced. NKR-2 will be supplied cryopreserved in bags containing a T-cell dose in accordance with the dose-level which is to be administered.

Rationale for the NKR-2 dose and regimen

NKR-2 has been used in the single infusion dose escalation clinical study CM-CS1 (NCT02203825) which evaluated the safety and clinical activity NKR-2 administration without prior lymphodepleting conditioning in patients with two distinct haematological malignancies: Acute Myeloid Leukaemia (AML)/ Higher Risk Myelodysplastic Syndrome (MDS) refractory anaemia with excess blasts, that are not in remission and for which standard therapy options are not available, and relapsed or refractory progressive Multiple Myeloma

For peer review only - http://bmjopen.bmj.com/site/about/guidelines.xhtml

(MM) after several lines of standard therapy. The trial has been successfully completed with no observed dose limiting toxicities at the highest dose infused ($3x10^7$ total cells) and initial signs of clinical benefit [57].

In preclinical models, the pharmacologically effective dose (PED) of the murine NKR-2 ranged from $1x10^6$ cells to $7.5x10^6$ cells per dose (intravenous (i.v.) single injection). Although a significant anti-tumour activity was obtained with a single injection of murine NKR-2, 100% overall survival and complete recovery was obtained following repeated three sequential doses of $5x10^6$ cells/mouse without any adverse effect [38, 39, 41, 49]. Moreover, a treatment regimen with two weeks' interval between injections was far more successful in terms of activity as compared to a weekly interval demonstrating that successful treatment of established tumours did not require the administration of more NKR-2 [49]. The maximum tolerated dose (MTD) after a single i.v. injection was determined to be $1x10^7$ cells, and repeated administration of this dosage to healthy and tumour-bearing mice did not produce any major toxicities [48].

Following these values of PED and MTD, the no-observed effect level (NOEL) was defined at $5x10^6$ cells/injection per mouse and were identical whatever the tumour type model (haematological or solid tumour). Using the allometry scaling exponent of 0.75, as defined by FDA guidelines (USFDA, 2005), human-equivalent PED and MTD were therefore estimated at 0.43 - $3.2x10^9$ and $4.3x10^9$ cells/injection, respectively. Together these observations of high tolerability in initial single dose phase I clinical trial coupled with pre-clinical efficacy experimentation strongly support the concept of a dose escalation segment of $3x10^8$ to $3x10^9$ NKR-2 per infusion given three times each separated by a 14-day interval.

Patient population

For peer review only - http://bmjopen.bmj.com/site/about/guidelines.xhtml

Refractory or relapsing patients with metastatic or locally advanced colorectal cancer (CRC), urothelial carcinoma, triple-negative breast cancer (TNBC), pancreatic cancer, recurrent epithelial ovarian and fallopian tube carcinoma, acute myeloid leukaemia AML/MDS and MM, which failed from standard treatments, will be recruited into this protocol (the key inclusion and exclusion criteria are presented in Table 2). The study will enrol patients in different countries in Europe and in USA with a dose escalation segment open to a restricted network.

Table 2: Key eligibility criteria

**Main inclusion criteria:**
Men or women ≥ 18 years old at the time of signing the ICF,
Patient with specific cancer indications (see below),
Disease must be measurable according to the corresponding guidelines,
ECOG performance status of 0 or 1 or 2 based on peripheral neuropathy from prior therapies,
Patient with adequate bone marrow reserve, hepatic and renal functions,
Left ventricular ejection fraction of > 40%
For patients with solid tumour type, the patient must agree to have a tumour biopsy at baseline.
**Main exclusion criteria:**
Patient with a tumour metastasis in the central nervous system,
Patients who have received another cancer therapy within 2 weeks before the planned day for the apheresis,
Patients who receive or are planned to receive any other investigational product within the 3 weeks before the planned day for the first NKR-2 administration,
Patients who are planned to receive concurrent growth factor, systemic steroid or other immunosuppressive therapy or cytotoxic agent,
Patients who underwent major surgery within 4 weeks before the planned day for the first NKR-2 administration,
Patients who have active infections necessitating use of antibiotics/antivirals treatment,
Patients with history of autoimmune disease.
**Disease-specific inclusion criteria**
Patients must have either:
A documented metastatic or locally advanced colorectal adenocarcinoma and having received, being intolerant, or having refused at least 2 prior standard cancer therapy regimens as part of their primary treatment regimen or part of their treatment for management of recurrent/persistent disease,
A documented recurrent epithelial ovarian cancer or fallopian tube carcinoma and having received or refused at least 2 prior standard cancer chemotherapy regimens as part of their primary treatment regimen or part of their treatment for management of recurrent/persistent disease,
An inoperable locally advanced or metastatic urothelial carcinoma that have progressed after previous platinum-based chemotherapy,
A metastatic or locally advanced triple-negative breast cancer (TNBC) and having received at least 1 prior cancer therapy regimen as part of their treatment for management of recurrent/persistent disease,
A metastatic or locally advanced pancreatic ductal adenocarcinoma and having received, being intolerant or having refused at maximum 2 prior lines of standard cancer chemotherapy regimens,

For peer review only - http://bmjopen.bmj.com/site/about/guidelines.xhtml

Relapsed or refractory AML (. ≥ 5% blasts in bone marrow or in peripheral blood) after one prior therapy or patients with myelodysplastic syndrome (. ≥ 5% blasts in bone marrow or ≥ 2% blasts in peripheral blood) or MDS with TP53 mutation who failed from prior treatments,<br>Relapsing or refractory/relapsing MM with a minimum of 1 prior line of systemic therapy

The tumour types targeted in this protocol were selected primarily based on their high expression of the NKG2D ligands (with a prevalence of 78-100% tumour samples expressing at least 1 ligand) [33, 34]. Furthermore, NKG2D ligand expression is a dynamic and context-dependent process, quickly varying over time in response to cytokines in the tumour microenvironment and evolving along with tumour progression, local inflammatory responses and treatments [33, 34], hence difficult to assess. Finally, even if NKG2D ligand expression is heterogeneous with respect to the exact ligands found, based on preclinical data, potential therapeutic benefit might be achieved in patients with highly heterogeneous tumours [47] or even tumours considered as “negative” for NKG2D ligands because of a NKG2D ligand positive tumour microenvironment and/or neovasculature and/or activation of host immune system [38, 41, 42, 49-51, 53]. Thus, no eligibility criteria were based upon the individual expression profile of NKG2D ligands and no diagnostic test for ligand expression used for recruitment into this study. Correlative studies, however, will be carried out during the trial and will attempt to investigate the expression profile of patient tumours with clinical response.

THINK study design

THINK Phase I study will contain two consecutive segments: a dose escalation and an expansion segment (Figure 3).

The **dose escalation** design will include 3 dose-levels of NKR-2. The assumption is made that the safety profile of the treatment could be different in haematological versus solid tumour

For peer review only

For peer review only - http://bmjopen.bmj.com/site/about/guidelines.xhtml

types. Therefore, it will be assessed separately in two different arms, one in solid tumours (CRC, urothelial carcinoma, TNBC, pancreatic cancer or epithelial ovarian and fallopian tube carcinoma) and one in haematological tumours (AML/MDS or MM). Each arm will use a 3+3 design to determine the recommended dose (RecD) of the NKR-2 treatment for the corresponding cohorts in the expansion segment.

The RecD will be further evaluated in the **expansion segment** of the study to assess separately the safety profile and initial clinical activity of the NKR-2 treatment in 7 cohorts of patients with the distinct tumour types (up to 14 patients per tumour type). The expansion segment in haematological and solid tumours will be initiated as soon as their respective dose escalation arm defines the RecD. During this segment, the RecD can still be adapted according to predefined safety rules in any specific tumour type.

18 patients in the dose escalation segment and up to 86 additional patients in the expansion segment of the study are expected to be enrolled in the study.

For peer review only

Treatment regimen

Three NKR-2 dose-levels will be evaluated during this study, $3x10^8$, $1x10^9$ and $3x10^9$ NKR-2 for each injection (adjusted to $4.6x10^6$, $1.5x10^7$ and $4.6x10^7$ NKR-2/kg, respectively for patients with body weight below or equal to 65 kg) with a schedule of administration of three NKR-2 doses administered with a 2-week interval per patient. No systemic lymphodepleting pre-conditioning will be performed prior to NKR-2 injections.

Trial Endpoints

The of the **dose escalation** segment will be the occurrence of dose limiting toxicities (DLTs) in all patients during the study treatment until 14 days after the last study

For peer review only - http://bmjopen.bmj.com/site/about/guidelines.xhtml

treatment administration. will include the occurrence of adverse events (AEs) and serious adverse events (SAEs) during the study treatment, occurrence of objective clinical response at D57, M3, M6, M9, M18 and M24 after 1st NKR-2 administration and according international guidelines, progression-free survival (PFS) and overall survival (OS), and duration of response for patients with objective response up to two years after first NKR-2 administration.

The of the **expansion segment** will be the objective response rate (ORR) at D57, M3, M6, M9, M18 and M24 after first NKR-2 administration. will include the PFS and OS, duration of response for patients with objective response up to two years after first NKR-2 administration and occurrence of AEs and SAEs and DLTs during the study treatment.

The overall aim of this segment is to understand the safety profile and develop initial observations of clinical activity in a cohort of up to 14 patients that support a deeper assessment of clinical efficacy of NKR2 in larger patient cohorts within a Phase II clinical study.

Response to treatment will be assessed according to standard RECIST criteria (version 1.1) for solid tumours or international recommendations for response criteria in haematological tumours. Apart from the visits scheduled per protocol, tumour assessment might also be performed at any time if considered as needed per investigator's decision (progression suspicion) and in regard to the RECIST 1.1. response confirmation.

Study duration

For peer review only

For peer review only - http://bmjopen.bmj.com/site/about/guidelines.xhtml

For each patient, the overall study duration will be 15 years. The study consists of a screening phase, a treatment administration phase, a treatment follow-up phase and a long-term safety follow-up (Figure 4). The duration of the administration phase will be 8 weeks, from Visit D1 to Visit D57. The duration of the follow-up phase will be 22 months, from the end of the administration phase on D57 (Week 8) to Visit M24.

As per regulatory requirements, patients who received at least one NKR-2 treatment will need to be followed annually for a period of 15 years. Therefore, patients will also follow a **long-term safety follow-up** that will last for up to 15 years after the day of the first NKR-2 administration to collect safety data for 13 additional years. The surveillance will mimic as much as possible the standard follow-up of the respective tumour type including monitoring of important safety parameters.

Patients may be withdrawn from the study treatment or from the study at any time at the patients' discretion or at their investigator's discretion. The investigator will document on the patient's record whether the decision to discontinue treatment and/or study participation was made by the patient or the investigator and which reason applies.

In case of absence of disease progression at M24, PFS and OS as clinical endpoints will be followed-up on a yearly basis or at , in case of progression/relapse suspicion. In case of disease progression at M24 or at any time before, the patient will be still asked to attend simplified follow-up procedures which will include (i) record of survival status, (ii) medication status and (iii) blood sampling for viral copy number (VCN) and replication competent retrovirus (RCR) assessment. Clinical progression at the end of the treatment phase (D57) will not to be considered as a criterion for withdrawal from the study, and will allow

For peer review only

For peer review only - http://bmjopen.bmj.com/site/about/guidelines.xhtml

the continuation of the active follow up of the patient with or without the initiation of a new anti-cancer therapy.

Enrolment rules

There is no requirement for specific recruitment of each individual tumour indications to corresponding arm within the **dose escalation phase**, but each dose-level should enrol at least two different types of cancer. In each arm, patients will be enrolled at the next dose-level when all evaluable patients at the same dose-level have completed the safety period of 14 days after last NKR-2 injection. Within each dose-level, patients will be enrolled as soon as the previous patient has received at least one NKR-2 administration and has completed the 14-day safety period after the 1st injection.

Enrolment in the **expansion segment** of the study will be performed in 3 steps, with a statistical analysis at the end of each step (see next section). Within each step, all patients can theoretically be enrolled at the same time.

Conduct of analysis and statistical considerations

All statistical analyses will be performed using SAS and/or StatXact software. The results will be reported per dose-level/tumour type cohort and overall.

Demographic characteristics (age, gender and race) physical examination, medical history and baseline tumor characteristics will be tabulated and analyzed with appropriate descriptive statistics on the .

The **analysis of safety** will be performed on the . All the AEs will be graded according to the NCI-CTCAE, Version 4.03 and will be coded to the preferred term level using the Medical Dictionary for Regulatory Activities (MedDRA). A summary of AEs

For peer review only

For peer review only - http://bmjopen.bmj.com/site/about/guidelines.xhtml

by maximum grade and cohort. The same analysis will be presented for SAEs and events with a suspected relationship to the study treatment. A summary of AEs by maximum grade and cohort per treatment dose will also be performed. SAEs, DLTs and discontinuation due to adverse events(s) will be described in detail by patient narratives.

A summary of the concomitant medications, classified by ATC term, will be displayed by cohort. Individual listing of concomitant medications per patient and per timepoint will also be provided. Finally, the total number of study treatments administered to each patient and the length of active follow-up will be summarized.

The **analysis of clinical activity** will be performed on the all patients which received at least one NKR-2 treatment (in case of patient's withdrawal prior the end of the administration phase). Sensitivity analyses related to the clinical activity endpoints will be performed on the population which includes all evaluable patients who have complied with all the procedures defined in the protocol up to concluding visit.

The first ORR will be assessed at Visit D57. The occurrence and duration of objective clinical response (CR, PR and SD) will be reported. Disease progression, including death, during the study will be described. Estimates of the rates of best clinical response and their 95% CIs will be reported. Kaplan-Meier curves will be estimated for PFS and OS.

In addition, other exploratory analyses may be performed, ., the clinical activity may be assessed in sub-populations based on the NKG2D ligands tumor expression, types of ligands expressed in tumor, serum levels of soluble NKG2D ligands, etc.

For peer review only

In the dose-escalation segment, safety data will be monitored closely on an ongoing basis. The main analysis (leading to the selection of the RecD) will be conducted separately in each arm once all patients in the last dose-level have received the 3 NKR-2 administrations and have completed the safety period (D43). All data related to the primary endpoints of the study will be analysed. The final analysis will be performed once all patients have attended the concluding visit of the follow-up phase (M24) or have been withdrawn from the study.

The expansion segment of the study will proceed in 3 steps, with a statistical analysis at the end of each step (Figure 3). The 6 patients from the dose escalation segment of the study who were treated at the RecD will be included in these expansion analyses. These steps aim to stop the study for futility in specific cohorts or specific tumour types in case of no (sufficient) responders in the corresponding cohorts at the time of analysis.

An interim analysis for futility will be performed on the first 14 patients over all 5 cohorts of patients with solid tumour types and on the first 6 patients over the two cohorts of patients with haematological tumour types who have either completed the administration phase (D57) or have withdrawn from the study.

The second step consists in cohort futility analyses after the first 7 cancer patients in each tumour type have either completed the administration phase (D57) or have withdrawn from the study. At this step, an evaluation of the safety of the NKR-2 treatment in each cohort will also be performed on the first 6 patients who completed the 6-week treatment period (D43) or had a DLT. This will allow to adapt the RecD in any specific cohort (tumour type) according to both safety rules and futility analysis. Indeed, if, and as soon as, 2 or more DLTs have been reported within a specific cohort, and the criterion for futility was not reached, the next patients enrolled in this cohort will be treated at the next lower dose-level. In contrast, if no

For peer review only - http://bmjopen.bmj.com/site/about/guidelines.xhtml

DLT has been reported within a specific cohort, and the criterion for futility was not reached at step 2, the next patients enrolled will be treated with the next higher dose-level.

Finally, the final evaluation of the objective clinical response rate will be evaluated once all the first 14 cancer patients in each cohort have either completed their administration period (D57) or have withdrawn from study due to disease progression or DLT.

Correlative studies

Peripheral blood samples, bone marrow aspirates from patients with haematological malignancies, as well as tumour biopsies from patients with solid malignancies, will be collected at regular time-points during the study (see Figure 4) and used to determine NKR-2 persistence post-infusion (using a viral copy number test) and assess the presence of replication competent retrovirus (RCR). Systemic cytokine levels in peripheral blood and levels of tumour-related biomarkers (in the serum, bone-marrow or urine samples) will be also assessed to determine whether correlates of NKR-2 activity can be identified. Evaluation and characterization of the NKR-2 mode of action, involving the characterization of the pre-existing and post/during-therapy humoral and cellular immune responses will be done on blood samples collected at baseline and post-NKR-2 infusion. Finally, the correlation with the tumour expression of NKG2D ligand in tumour tissues or cells (type and level) and NKR-2 activity, the characterization of the tumour microenvironment (TME), the NKR-2 infiltration and the modulation of the local tumour environment of this new type of T-cell therapy will be evaluated.

Toxicity Management

As previously mentioned, the use of CAR T-cells is limited by potentially severe toxicities including cytokine release syndrome (CRS), neurotoxicity and tumour lysis syndrome (TLS),

For peer review only - http://bmjopen.bmj.com/site/about/guidelines.xhtml

on-target off-tumour toxicity (related to recognition of the target antigen on normal tissues), clonality and insertional oncogenesis, vector persistence and immunogenicity. While these events may have been seen in relation with other CAR-T therapies, and are provided for information, there are currently no events considered as expected for NKR-2 therapy (which can be different from other CAR-T studies based on the unique mode of action) and all Serious Adverse Reactions (SARs) will be considered as suspected unexpected SARs (SUSARs).

The management of toxicities related to CAR-T therapies was described elsewhere [11, 12, 58] but is evolving with everyday new information available from ongoing studies. Sites will be kept informed all along the study for any new therapeutic options modifications or standardization between the different sites.

Briefly, CRS toxicity is currently of highest concern in the field. It is caused by a release of inflammatory cytokines such as IL-6, IL-2, IFN-γ, and TNF-α, following the recognition of the target antigen, resulting in a systemic inflammatory response similar to sepsis. Therefore, CRS will correlate with both efficacy and toxicity in patients receiving T cell-engaging therapies. Tocilizumab (anti IL-6) will be used as the first-line agent in the treatment of CRS. If the patient's condition does not improve or stabilize within 24 hours of the tocilizumab dose, administration of a second dose of tocilizumab and/or a second immunosuppressive agent (such as corticosteroids) will be considered, based on the CRS revised grading system created by Lee et al. [59] to identifying severity and implementation of optimal care strategies for CRS.

For peer review only - http://bmjopen.bmj.com/site/about/guidelines.xhtml

TLS resulting in renal insufficiency, rapidly rising uric acid level, or evidence of organ dysfunction will be managed with fluids and rasburicase as needed and as determined by the treating physicians.

Further, as NKG2D ligands may be upregulated upon stress signals, expression of ligands on non-cancer cells can be expected in tissues that are actively infected or inflamed or experienced cell stress of other aetiology. Infections could therefore invoke a significant inflammatory response by NKR-2 by potentially inducing a cytokine release syndrome due to the increased recognition of ligands by NKR-2 cells. Therefore, any inflammation, infection, concurrent or previous treatment with anti-cancer therapies, will be carefully registered and any Grade 2 or higher autoimmune toxicity will be closely followed to detect any evidence of on-target off-tumour toxicity. Evidence of acute infection, as well as fever of 38°C/100°F or above, will be therefore considered as exclusion criteria at screening (see Table 2) or postponement criteria when observed during the treatment phase. In the latter, all study procedures and treatment administration will be resumed where it was left as soon as the patient's condition allows (within 4 weeks after the scheduled treatment date) or at the investigator's discretion after Steering Committee and Sponsor approvals.

Since NKR-2 construct consists entirely of human sequences, it is not expected to result in immunogenicity sometimes observed with other CAR-Ts using scFv from murine origin (see introduction), and therefore multiple NKR-2 injections are allowed without the anticipated risk of humoral immune responses.

Uncontrolled expansion and proliferation of the transformed cells increases the risks for onset of cytokine release syndrome and other CAR-associated toxicities. However, in contrast to more classical CAR-T therapies, NKR-2 demonstrated neither significant expansion

For peer review only - http://bmjopen.bmj.com/site/about/guidelines.xhtml

nor long-term persistence until now [39], but since this is the first trial with NKR-2 administrated as multiple doses, the persistence of NKR-2 will be nevertheless carefully monitored post-infusion as mandatory exploratory research. If uncontrolled T-cell proliferation occurs (grade 3 or 4 toxicity related to NRK-2), and as it was reported that CAR associated toxicity respond to systemic corticosteroids, patients may be treated with corticosteroids and/or chemotherapy to eradicate the CAR T-cells [60]. Patients will be treated with pulse, followed by a rapid taper.

Finally, following FDA recommendation for Retroviral and Lentiviral Vector Based Gene Therapy Products, the patient monitoring schedule will also include analysis of the replication-competent retrovirus (RCR) of patient samples up to 15 years after CAR-T injections.

For peer review only - http://bmjopen.bmj.com/site/about/guidelines.xhtml

Discussion

The THINK trial aims primarily to determine the safety and clinical activity of NKR-2 cell therapy, without pre-conditioning chemotherapy, in patients with advanced cancer. The dose escalation phase of the trial is designed to determine the recommended dose in haematological and solid tumours, which then is used in the expansion phase to assess clinical responses separately in seven different indications. Due to the multi-targeting specificity of NKR-2, the dose escalation and subsequent expansion segments are designed to facilitate a rapid assessment of NKR-2 activity across a range of tumours. This is advantageous since the assessment of safety and any evidence of clinical activity will be suitably evaluated across cancer types using a consistently produced cell product thereby avoiding issues that arise in multiple single tumour indication trials testing a single T-cell product. Interestingly, the number of ligands targeted by NKR-2 (8 ligands from 2 main families), combined with their stress-induced tissue expression, also limit the potential risk of clonal selection or antigen loss after injection, which has been observed for classical CAR-T constructs targeting a single antigen. This is expected to translate into limiting the risk for potential relapse.

For patients, the lack of pre-conditioning chemotherapy removes the need for an in-patient stay during the transient period of lymphodepletion translating to a likely improved patient experience of the trial. Additionally, the absence of pre-conditioning also will reduce the potential toxicity of this treatment and facilitate providing a therapeutic solution to a larger patient population, including, notably, the elderly population.

The challenge facing classical CAR-T therapies in the human patient with advanced cancer (beyond B-cell malignancies) is major and, in the solid tumour situation, requires the cells to traffic to sites of tumour, overcome the highly suppressive tumour microenvironment and

For peer review only - http://bmjopen.bmj.com/site/about/guidelines.xhtml

initiate direct tumour cell killing to drive a sustained anti-tumour response [23-25]. Based on the different intrinsic mechanisms of action of NKR-2 observed in preclinical experiments on various tumour models, NKR-2 should overcome, at least partially, the local immunosuppression and target expression heterogenicity in tumour tissues, while, because of its effect on tumour vasculature, trafficking into tumours might also be enhanced. Furthermore, the multiple infusion strategy was also found to increase activity and infiltration into tumours of other short-term persistent CAR T-cells (mesothelin-specific RNA CAR-T cells) [61], overcome the potential tumour-induced T-cell dysfunction upon interaction of CAR-T cells with the immunosuppressive tumour microenvironment [23], while being well tolerated in humans [62]. Consequently, as compared to classical CARs, clinical activity in solid tumours might be observed after treatment with NKR-2, without any construct modification nor combination with other immunotherapies. Since THINK is the first trial to evaluate the potential clinical activity of a multiple NKR-2 administration schedule in solid tumours, it offers a real opportunity as compared with classical CAR-Ts within the solid tumour type field, where therapeutic options are limited. Further impacting the tumor environment through combinations of chemotherapy or immune modulation will constitute subsequent steps to consider once we have progressed sufficiently in the THINK trial and demonstrated safety of NKR-2 administrations.

The multiple infusion strategy, absence of pre-conditioning chemotherapy, diversity of putative mechanisms of action, as well as the diversity of tumour types that can potentially targeted, therefore stand the NKR-2 CAR T-cell approach described in this protocol apart from the current ‘gold standard’ CAR T-cell therapy paradigm. In this context, the THINK trial provides a first approach of a global comprehensive clinical program embedding other

For peer review only - http://bmjopen.bmj.com/site/about/guidelines.xhtml

phase I clinical protocols that will evaluate the combination of the NKR-2 with specific anti-cancer treatments and the loco-regional administration, among others.

For peer review only

For peer review only - http://bmjopen.bmj.com/site/about/guidelines.xhtml

Ethics and dissemination

The THINK trial (A Dose Escalation Phase I Study to Assess the Safety and Clinical Activity of Multiple Cancer Indications) is currently recruiting participants in Europe and USA. Ethical approval has been obtained at all sites open for recruitment. The trial has been approved by ClinicalTrials.gov (Trial registration ID NCT03018405) and by EU Clinical Trials Register (EudraCT number: 2016-003312-12). The present publication refers to protocol version 2.0 (released the 10th of February 2017). Written informed consent will be taken from all participants. Results of this study will be disseminated through presentation at international scientific conferences and reported in peer-reviewed scientific journals.

For peer review only

For peer review only - http://bmjopen.bmj.com/site/about/guidelines.xhtml

Abbreviations

AE: adverse event; AML: acute myeloid leukaemia; CAR: chimeric antigen receptor; chNKG2D: NKG2D-based CAR; CR: complete response; CRC: colorectal cancer; CRS: cytokine release syndrome; DAP10: DNAX-activating protein of 10 kDa; DLT: dose-limiting toxicity; MDS: myelodysplastic syndrome; MDSC: myeloid-derived suppressor cell; MHC: major histocompatibility complex; MICA/B: MHC class I chain related proteins A/B; MM: multiple myeloma; MTD: maximum tolerated dose; NK: natural killer; NKG2D: natural killer group 2D; NKR-2: human NKG2D receptor-based CAR T-cells; NOEL: no observed effect level; ORR: objective response rate; OS: overall survival; PBMC: peripheral blood mononuclear cell; PED: pharmacologically effective dose; PFS: progression-free survival; PR: partial response; RAEB: refractory anaemia with excess blasts; RCR: replication competent retrovirus; RecD: recommended dose for expansion segment; SAR: serious adverse reactions; SAE: serious adverse event; scFv: single-chain variable fragment; sMICA/B or sULBP1-6: serum level of MICA/B or ULBP1-6; SD: stable disease; SUSAR: suspected unexpected SAR; THINK: THerapeutic Immunotherapy with NKR-2; TLS: tumour lysis syndrome; TME: tumour microenvironment; TNBC: triple-negative breast cancer; Treg: regulatory T-cell; ULBP1-6: unique long 16 binding protein 1-6.

For peer review only

For peer review only - http://bmjopen.bmj.com/site/about/guidelines.xhtml

References

1. Fesnak AD, June CH, Levine BL. Engineered T cells: the promise and challenges of cancer immunotherapy. 2016;16(9):566-81.

2. Jackson HJ, Rafiq S, Brentjens RJ. Driving CAR T-cells forward. 2016;13(6):370-83.

3. Dai H, Wang Y, Lu X, et al. Chimeric Antigen Receptors Modified T-Cells for Cancer Therapy. ! 2016;108(7).

4. Gross G, Eshhar Z. Therapeutic Potential of T Cell Chimeric Antigen Receptors (CARs) in Cancer Treatment: Counteracting Off-Tumor Toxicities for Safe CAR T Cell Therapy. " # $ 2016;56:59-83.

5. Pule MA, Savoldo B, Myers GD, et al. Virus-specific T cells engineered to coexpress tumor-specific receptors: persistence and antitumor activity in individuals with neuroblastoma. % 2008;14(11):1264-70.

6. Rosenberg SA, Yang JC, Sherry RM, et al. Durable complete responses in heavily pretreated patients with metastatic melanoma using T-cell transfer immunotherapy. 2011;17(13):4550-7.

7. Maude SL, Frey N, Shaw PA, et al. Chimeric antigen receptor T cells for sustained remissions in leukemia. & % 2014;371(16):1507-17.

8. Lee DW, Kochenderfer JN, Stetler-Stevenson M, et al. T cells expressing CD19 chimeric antigen receptors for acute lymphoblastic leukaemia in children and young adults: a phase 1 dose-escalation trial. # ' 2015;385(9967):517-28.

9. Grupp SA, Kalos M, Barrett D, et al. Chimeric antigen receptor-modified T cells for acute lymphoid leukemia. & % 2013;368(16):1509-18.

For peer review only - http://bmjopen.bmj.com/site/about/guidelines.xhtml

10. Gardner R, Wu D, Cherian S, et al. Acquisition of a CD19-negative myeloid phenotype allows immune escape of MLL-rearranged B-ALL from CD19 CAR-T-cell therapy. (2016;127(20):2406-10.

11. Bonifant CL, Jackson HJ, Brentjens RJ, et al. Toxicity and management in CAR T-cell therapy. % # 2016;3:16011.

12. Brudno JN, Kochenderfer JN. Toxicities of chimeric antigen receptor T cells: recognition and management. (2016;127(26):3321-30.

13. Brentjens RJ, Riviere I, Park JH, et al. Safety and persistence of adoptively transferred autologous CD19-targeted T cells in patients with relapsed or chemotherapy refractory B-cell leukemias. (2011;118(18):4817-28.

14. Kochenderfer JN, Dudley ME, Feldman SA, et al. B-cell depletion and remissions of malignancy along with cytokine-associated toxicity in a clinical trial of anti-CD19 chimeric-antigen-receptor-transduced T cells. (2012;119(12):2709-20.

15. Kershaw MH, Westwood JA, Parker LL, et al. A phase I study on adoptive immunotherapy using gene-modified T cells for ovarian cancer. 2006;12(20 Pt 1):6106-15.

16. Lamers CH, Willemsen R, van Elzakker P, et al. Immune responses to transgene and retroviral vector in patients treated with ex vivo-engineered T cells. (2011;117(1):72-82.

17. Turtle CJ, Hanafi LA, Berger C, et al. CD19 CAR-T cells of defined CD4+:CD8+ composition in adult B cell ALL patients. ! 2016;126(6):2123-38.

18. Maus MV, Haas AR, Beatty GL, et al. T cells expressing chimeric antigen receptors can cause anaphylaxis in humans. ! 2013;1(1):26-31.

For peer review only

For peer review only - http://bmjopen.bmj.com/site/about/guidelines.xhtml

19. Khalil DN, Smith EL, Brentjens RJ, et al. The future of cancer treatment: immunomodulation, CARs and combination immunotherapy. 2016;13(5):273-90.

20. Lamers CH, Langeveld SC, Groot-van Ruijven CM, et al. Gene-modified T cells for adoptive immunotherapy of renal cell cancer maintain transgene-specific immune functions in vivo. ! ! 2007;56(12):1875-83.

21. Morgan RA, Yang JC, Kitano M, et al. Case report of a serious adverse event following the administration of T cells transduced with a chimeric antigen receptor recognizing ERBB2. % # 2010;18(4):843-51.

22. Beatty GL, O'Hara M. Chimeric antigen receptor-modified T cells for the treatment of solid tumors: Defining the challenges and next steps. # 2016;166:30-9.

23. Moon EK, Wang LC, Dolfi DV, et al. Multifactorial T-cell hypofunction that is reversible can limit the efficacy of chimeric antigen receptor-transduced human T cells in solid tumors. 2014;20(16):4262-73.

24. Newick K, Moon E, Albelda SM. Chimeric antigen receptor T-cell therapy for solid tumors. % # 2016;3:16006.

25. Abken H. Adoptive therapy with CAR redirected T cells: the challenges in targeting solid tumors. ! 2015;7(5):535-44.

26. Han S, Latchoumanin O, Wu G, et al. Recent clinical trials utilizing chimeric antigen receptor T cells therapies against solid tumors. ' 2017;390:188-200.

27. Beatty GL, Moon EK. Chimeric antigen receptor T cells are vulnerable to immunosuppressive mechanisms present within the tumor microenvironment. 2014;3(11):e970027.

For peer review only

For peer review only - http://bmjopen.bmj.com/site/about/guidelines.xhtml

28. Lanier LL. NKG2D Receptor and Its Ligands in Host Defense. ! 2015;3(6):575-82.

29. Garrity D, Call ME, Feng J, et al. The activating NKG2D receptor assembles in the membrane with two signaling dimers into a hexameric structure. ") " 2005;102(21):7641-6.

30. Gilfillan S, Ho EL, Cella M, et al. NKG2D recruits two distinct adapters to trigger NK cell activation and costimulation. ! 2002;3(12):1150-5.

31. Carapito R, Bahram S. Genetics, genomics, and evolutionary biology of NKG2D ligands. ! 2015;267(1):88-116.

32. Raulet DH, Gasser S, Gowen BG, et al. Regulation of ligands for the NKG2D activating receptor. " ! 2013;31:413-41.

33. Le Bert N, Gasser S. Advances in NKG2D ligand recognition and responses by NK cells. ! (2014;92(3):230-6.

34. Spear P, Wu M-R, Sentman M-L, et al. NKG2D ligands as therapeutic targets. ! " 2013;13:8.

35. Holdenrieder S, Stieber P, Peterfi A, et al. Soluble MICB in malignant diseases: analysis of diagnostic significance and correlation with soluble MICA. ! ! 2006;55(12):1584-9.

36. Salih HR, Rammensee HG, Steinle A. Cutting Edge: Down-Regulation of MICA on Human Tumors by Proteolytic Shedding. ! 2002;169(8):4098-102.

37. Zhang T, Lemoi BA, Sentman CL. Chimeric NK-receptor-bearing T cells mediate antitumor immunotherapy. (2005;106(5):1544-51.

For peer review only - http://bmjopen.bmj.com/site/about/guidelines.xhtml

38. Zhang T, Barber A, Sentman CL. Chimeric NKG2D modified T cells inhibit systemic T-cell lymphoma growth in a manner involving multiple cytokines and cytotoxic pathways. 2007;67(22):11029-36.

39. Barber A, Meehan KR, Sentman CL. Treatment of multiple myeloma with adoptively transferred chimeric NKG2D receptor-expressing T cells. * # 2011;18(5):509-16.

40. Barber A, Zhang T, DeMars LR, et al. Chimeric NKG2D receptor-bearing T cells as immunotherapy for ovarian cancer. 2007;67(10):5003-8.

41. Barber A, Zhang T, Sentman CL. Immunotherapy with Chimeric NKG2D Receptors Leads to Long-Term Tumor-Free Survival and Development of Host Antitumor Immunity in Murine Ovarian Cancer. ! 2008;180:72-78.

42. Spear P, Barber A, Sentman CL. Collaboration of chimeric antigen receptor (CAR)-expressing T cells and host T cells for optimal elimination of established ovarian tumors. 2013;2(4):e23564.

43. Zhang T, Sentman CL. Mouse tumor vasculature expresses NKG2D ligands and can be targeted by chimeric NKG2D-modified T cells. ! 2013;190(5):2455-63.

44. Barber A, Zhang T, Megli CJ, et al. Chimeric NKG2D receptor-expressing T cells as an immunotherapy for multiple myeloma. &$ + 2008;36(10):1318-28.

45. Zhang T, Barber A, Sentman CL. Generation of antitumor responses by genetic modification of primary human T cells with a chimeric NKG2D receptor. 2006;66(11):5927-33.

46. Demoulin B, Cook WJ, Murad J, et al. Exploiting natural killer group 2D receptors for CAR T-cell therapy. , 2017.

For peer review only - http://bmjopen.bmj.com/site/about/guidelines.xhtml

47. Spear P, Barber A, Rynda-Apple A, et al. NKG2D CAR T-cell therapy inhibits the growth of NKG2D ligand heterogeneous tumors. ! (2013;91(6):435-40.

48. Sentman ML, Murad JM, Cook WJ, et al. Mechanisms of Acute Toxicity in NKG2D Chimeric Antigen Receptor T Cell-Treated Mice. ! 2016;197(12):4674-85.

49. Barber A, Rynda A, Sentman CL. Chimeric NKG2D expressing T cells eliminate immunosuppression and activate immunity within the ovarian tumor microenvironment. ! 2009;183(11):6939-47.

50. Barber A, Sentman CL. Chimeric NKG2D T cells require both T cell- and host-derived cytokine secretion and perforin expression to increase tumor antigen presentation and systemic immunity. ! 2009;183(4):2365-72.

51. Spear P, Barber A, Rynda-Apple A, et al. Chimeric antigen receptor T cells shape myeloid cell function within the tumor microenvironment through IFN-gamma and GM-CSF. ! 2012;188(12):6389-98.

52. Sentman CL, Meehan KR. NKG2D CARs as cell therapy for cancer. 2014;20(2):156-9.

53. Barber A, Sentman CL. NKG2D receptor regulates human effector T-cell cytokine production. (2011;117(24):6571-81.

54. Tey SK. Adoptive T-cell therapy: adverse events and safety switches. # ! 2014;3(6):e17.

55. Ertl HC, Zaia J, Rosenberg SA, et al. Considerations for the clinical application of chimeric antigen receptor T cells: observations from a recombinant DNA Advisory Committee Symposium held June 15, 2010. 2011;71(9):3175-81.

56. Minagawa K, Zhou X, Mineishi S, et al. Seatbelts in CAR therapy: How Safe Are CARS? (2015;8(2):230-49.

For peer review only - http://bmjopen.bmj.com/site/about/guidelines.xhtml

57. A first-in-human phase I trial of NKG2D chimeric antigen receptor-T cells in AML/MDS and multiple myeloma. 2016 ASCO Annual Meeting; 2016. J Clin Oncol.

58. Davila ML, Riviere I, Wang X, et al. Efficacy and toxicity management of 19-28z CAR T cell therapy in B cell acute lymphoblastic leukemia. # % 2014;6(224):224ra25.

59. Lee DW, Gardner R, Porter DL, et al. Current concepts in the diagnosis and management of cytokine release syndrome. (2014;124(2):188-95.

60. Walker RE, Bechtel CM, Natarajan V, et al. Long-term in vivo survival of receptor-modified syngeneic T cells in patients with human immunodeficiency virus infection. (2000;96(2):467-74.

61. Barrett DM, Liu X, Jiang S, et al. Regimen-specific effects of RNA-modified chimeric antigen receptor T cells in mice with advanced leukemia. + * # 2013;24(8):717-27.

62. Beatty GL, Haas AR, Maus MV, et al. Mesothelin-specific chimeric antigen receptor mRNA-engineered T cells induce anti-tumor activity in solid malignancies. ! 2014;2(2):112-20.

For peer review only - http://bmjopen.bmj.com/site/about/guidelines.xhtml

Footnotes

Acknowledgements

The delivery of successful clinical trials is primarily due to the excellence of the clinical partners with whom Celyad collaborates. The authors wish to thank all investigators in US and EU and all clinical managers for their valuable support and guidance. Finally, we thank all the patients and their families for participating to the study and for encouraging us to do this research.

Authors' contributions

CL, DG and FL conceived the study design, developed essential study documents and drafted the manuscript. BV, DG and FL participate in the coordination of the study. FP participated in the study design and the statistical review. AH, PA, AA, EVDN, GC, JPM, JB, DS, TK and KO participate in the management, will treat patients and conduct the clinical trial. MD gave support and guidance during the study design conception. All authors read, reviewed and approved the final manuscript.

Competing interests

CL, BV, DG and FL are employed by Celyad SA. JB and PA declare no disclosures. JPM has advisory roles with Merck Sharp and Dohme (uncompensated), Innate Pharma, Debio, Boehringer-Ingelheim, Nanobiotix. The THINK clinical trial is sponsored by Celyad SA.

Data sharing

Access to the dataset will only be considered when the primary results of the study have been published.

For peer review only
For peer review only - http://bmjopen.bmj.com/site/about/guidelines.xhtml

Figure legends

Figure 1: The NKR-2 construct. NKR-2 design contains the full-length human NKG2D receptor linked to the signalling domain of CD3 that provides primary signalling (signal 1) to activate T-cells upon ligand binding. The naturally expressed adaptor molecule DAP10 provides secondary signalling (signal 2) which allows NKR-2 to work as a second-generation CAR-T.

Figure 2: NKR-2 different mechanisms of action. Apart from direct cytotoxicity against cancer cells, NKR-2 mode of action also involves reduction of blood vessel density indicative of an anti-angiogenic activity, modulation of the immunosuppressive tumour microenvironment and induction of a long-term anti-tumour specific memory immune response.

Figure 3: Overview of the study design. ORR= objective response rate, RecD = recommended dose.

Figure 4: Overview of study phases. NKR-2 will be infused on Day 1, 15 and 29. Tumour assessment will be done by tumour imaging and blood sampling for solid tumours and tumour imaging, blood, urine and bone marrow sampling for haematological tumours. D = day, M = Month, BM = bone marrow.

For peer review only

For peer review only - http://bmjopen.bmj.com/site/about/guidelines.xhtml

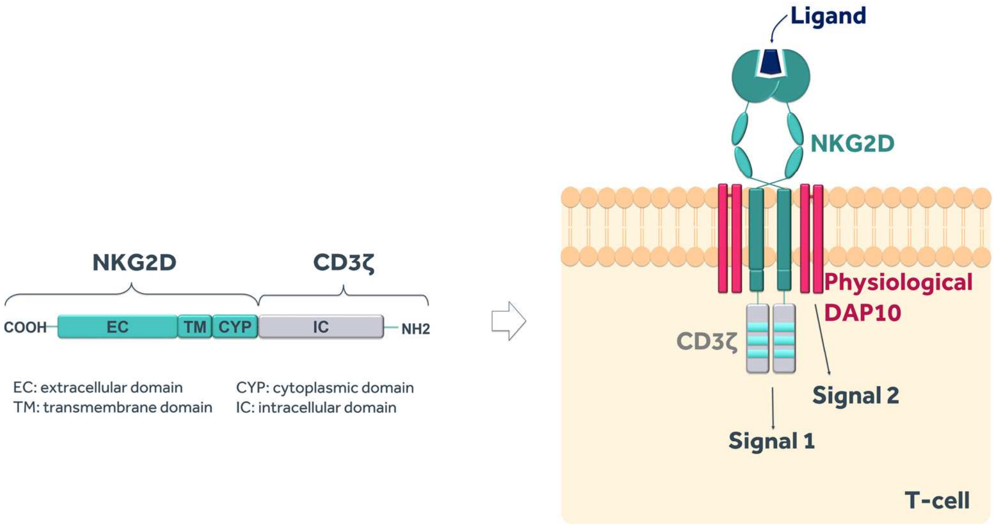

Figure 1: The NKR-2 construct. NKR-2 design contains the full-length human NKG2D receptor linked to the signalling domain of CD3ζ that provides primary signalling (signal 1) to activate T-cells upon ligand binding. The naturally expressed adaptor molecule DAP10 provides secondary signalling (signal 2) which allows NKR-2 to work as a second-generation CAR-T.

161x86mm (300 x 300 DPI)

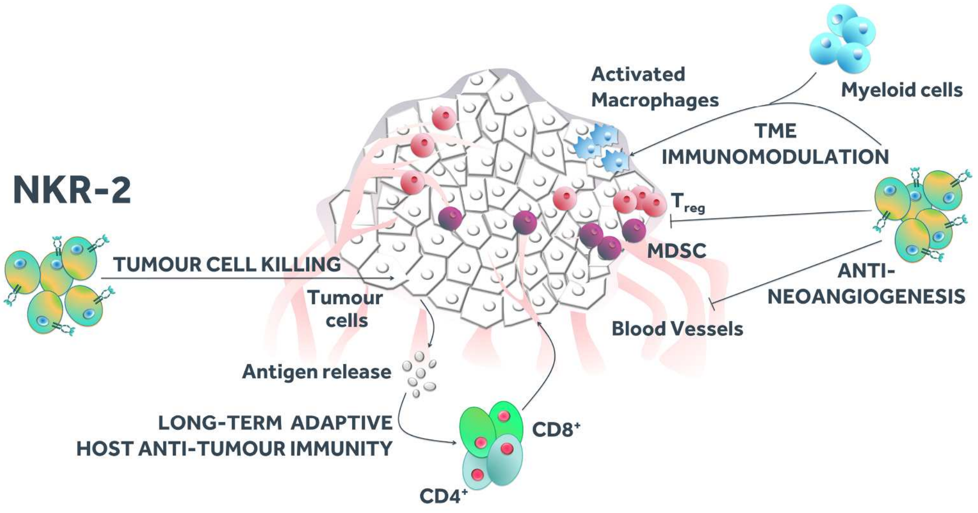

Figure 2: NKR-2 different mechanisms of action. Apart from direct cytotoxicity against cancer cells, NKR-2 mode of action also involves reduction of blood vessel density indicative of an anti-angiogenic activity, modulation of the immunosuppressive tumour microenvironment and induction of a long-term anti-tumour specific memory immune response.

233x120mm (300 x 300 DPI)

For peer review only

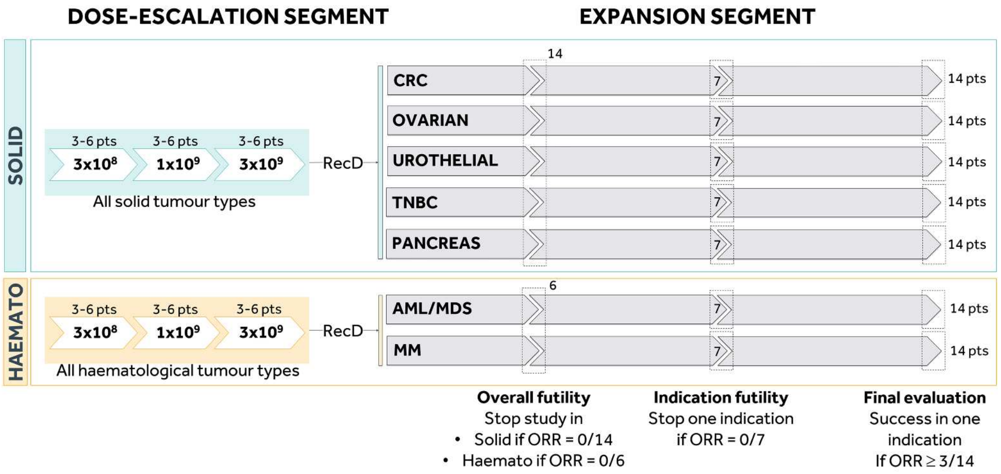

Figure 3: Overview of the study design. ORR= objective response rate, RecD = recommended dose.

242x118mm (300 x 300 DPI)

For peer review only

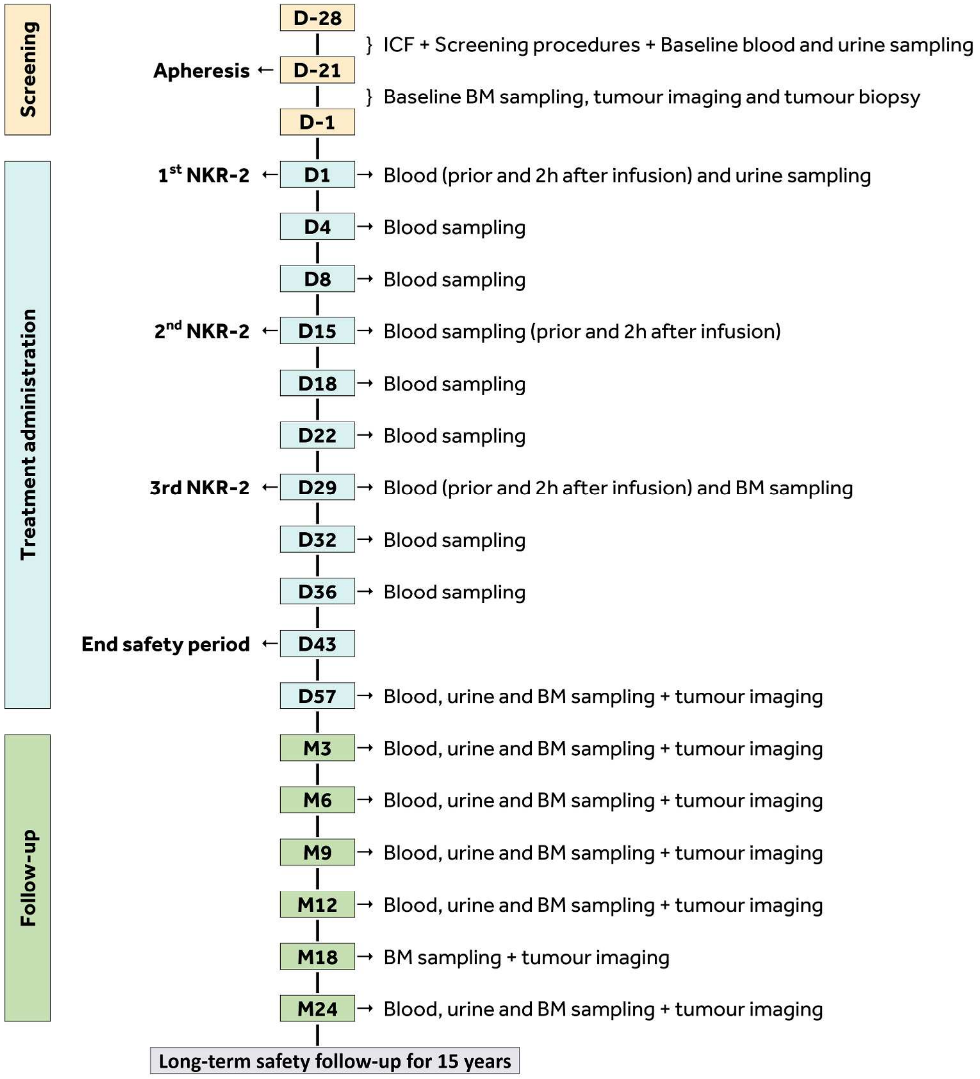

Figure 4: Overview of study phases. NKR-2 will be infused on Day 1, 15 and 29. Tumour assessment will be done by tumour imaging and blood sampling for solid tumours and tumour imaging, blood, urine and bone marrow sampling for haematological tumours. D = day, M = Month, BM = bone marrow.

326x355mm (300 x 300 DPI)

SPIRIT
STANDARD PROTOCOL ITEMS: RECOMMENDATIONS FOR INTERVENTIONAL TRIALS

SPIRIT 2013 Checklist: Recommended items to address in a clinical trial protocol and related documents*

For peer review only

Section/item	Item No	Description	Addressed on page number
**Administrative information**			
Title	1	Descriptive title identifying the study design, population, interventions, and, if applicable, trial acronym	Page 1
Trial registration	2a	Trial identifier and registry name. If not yet registered, name of intended registry	Page 3
	2b	All items from the World Health Organization Trial Registration Data Set	N/A
Protocol version	3	Date and version identifier	Page 25
Funding	4	Sources and types of financial, material, and other support	Page 31
Roles and responsibilities	5a	Names, affiliations, and roles of protocol contributors	Page 31
	5b	Name and contact information for the trial sponsor	Page 1
	5c	Role of study sponsor and funders, if any, in study design; collection, management, analysis, and interpretation of data; writing of the report; and the decision to submit the report for publication, including whether they will have ultimate authority over any of these activities	Page 25
	5d	Composition, roles, and responsibilities of the coordinating centre, steering committee, endpoint adjudication committee, data management team, and other individuals or groups overseeing the trial, if applicable (see Item 21a for data monitoring committee)	Sections 12.5.2 and 12.5.3 of the full-length protocol

For peer review only - http://bmjopen.bmj.com/site/about/guidelines.xhtml

**Introduction**			
Background and rationale	6a	Description of research question and justification for undertaking the trial, including summary of relevant studies (published and unpublished) examining benefits and harms for each intervention	Pages 7-10
	6b	Explanation for choice of comparators	N/A
Objectives	7	Specific objectives or hypotheses	Page 11
Trial design	8	Description of trial design including type of trial (eg, parallel group, crossover, factorial, single group), allocation ratio, and framework (eg, superiority, equivalence, noninferiority, exploratory)	Page 11, Figure 3
**Methods: Participants, interventions, and outcomes**			
Study setting	9	Description of study settings (eg, community clinic, academic hospital) and list of countries where data will be collected. Reference to where list of study sites can be obtained	Page 25
Eligibility criteria	10	Inclusion and exclusion criteria for participants. If applicable, eligibility criteria for study centres and individuals who will perform the interventions (eg, surgeons, psychotherapists)	Page 34
Interventions	11a	Interventions for each group with sufficient detail to allow replication, including how and when they will be administered	Figure 4
	11b	Criteria for discontinuing or modifying allocated interventions for a given trial participant (eg, drug dose change in response to harms, participant request, or improving/worsening disease)	Page 16 + Section 6.4.1 of the full-length protocol
	11c	Strategies to improve adherence to intervention protocols, and any procedures for monitoring adherence (eg, drug tablet return, laboratory tests)	Section 12.1 of the full-length protocol
	11d	Relevant concomitant care and interventions that are permitted or prohibited during the trial	Section 6.5.1 of the full-length protocol

For peer review only - http://bmjopen.bmj.com/site/about/guidelines.xhtml

Outcomes	12	Primary, secondary, and other outcomes, including the specific measurement variable (eg, systolic blood pressure), analysis metric (eg, change from baseline, final value, time to event), method of aggregation (eg, median, proportion), and time point for each outcome. Explanation of the clinical relevance of chosen efficacy and harm outcomes is strongly recommended	Page 15
Participant timeline	13	Time schedule of enrolment, interventions (including any run-ins and washouts), assessments, and visits for participants. A schematic diagram is highly recommended (see Figure)	Figure 4
Sample size	14	Estimated number of participants needed to achieve study objectives and how it was determined, including clinical and statistical assumptions supporting any sample size calculations	Page 14 + 18
Recruitment	15	Strategies for achieving adequate participant enrolment to reach target sample size	Section 5.1 of the full-length protocol
**Methods: Assignment of interventions (for controlled trials)**			
Allocation:			
Sequence generation	16a	Method of generating the allocation sequence (eg, computer-generated random numbers), and list of any factors for stratification. To reduce predictability of a random sequence, details of any planned restriction (eg, blocking) should be provided in a separate document that is unavailable to those who enrol participants or assign interventions	N/A
Allocation concealment mechanism	16b	Mechanism of implementing the allocation sequence (eg, central telephone; sequentially numbered, opaque, sealed envelopes), describing any steps to conceal the sequence until interventions are assigned	N/A
Implementation	16c	Who will generate the allocation sequence, who will enrol participants, and who will assign participants to interventions	N/A
Blinding (masking)	17a	Who will be blinded after assignment to interventions (eg, trial participants, care providers, outcome assessors, data analysts), and how	N/A
	17b	If blinded, circumstances under which unblinding is permissible, and procedure for revealing a participant’s allocated intervention during the trial	N/A
**Methods: Data collection, management, and analysis**			

For peer review only - http://bmjopen.bmj.com/site/about/guidelines.xhtml

Data collection methods	18a	Plans for assessment and collection of outcome, baseline, and other trial data, including any related processes to promote data quality (eg, duplicate measurements, training of assessors) and a description of study instruments (eg, questionnaires, laboratory tests) along with their reliability and validity, if known. Reference to where data collection forms can be found, if not in the protocol	Section 12 of the full-length protocol
	18b	Plans to promote participant retention and complete follow-up, including list of any outcome data to be collected for participants who discontinue or deviate from intervention protocols	Page 16
Data management	19	Plans for data entry, coding, security, and storage, including any related processes to promote data quality (eg, double data entry; range checks for data values). Reference to where details of data management procedures can be found, if not in the protocol	Sections 13.1 and 13.4 of the full-length protocol
Statistical methods	20a	Statistical methods for analysing primary and secondary outcomes. Reference to where other details of the statistical analysis plan can be found, if not in the protocol	Page 17-19 + Statistical plan
	20b	Methods for any additional analyses (eg, subgroup and adjusted analyses)	Pages 17-19 + Statistical plan (appendix to the original protocol)
	20c	Definition of analysis population relating to protocol non-adherence (eg, as randomised analysis), and any statistical methods to handle missing data (eg, multiple imputation)	Pages 17-19 + Statistical plan
**Methods: Monitoring**			
Data monitoring	21a	Composition of data monitoring committee (DMC); summary of its role and reporting structure; statement of whether it is independent from the sponsor and competing interests; and reference to where further details about its charter can be found, if not in the protocol. Alternatively, an explanation of why a DMC is not needed	Section 12.5.2 of the full-length protocol
	21b	Description of any interim analyses and stopping guidelines, including who will have access to these interim results and make the final decision to terminate the trial	Pages 17-19
Harms	22	Plans for collecting, assessing, reporting, and managing solicited and spontaneously reported adverse events and other unintended effects of trial interventions or trial conduct	Section 9.2 of the full-length protocol

For peer review only - http://bmjopen.bmj.com/site/about/guidelines.xhtml

Auditing	23	Frequency and procedures for auditing trial conduct, if any, and whether the process will be independent from investigators and the sponsor	Section 12.5.4 of the full-length protocol
**Ethics and dissemination**			
Research ethics approval	24	Plans for seeking research ethics committee/institutional review board (REC/IRB) approval	N/A (approved)
Protocol amendments	25	Plans for communicating important protocol modifications (eg, changes to eligibility criteria, outcomes, analyses) to relevant parties (eg, investigators, REC/IRBs, trial participants, trial registries, journals, regulators)	Section 12.1 of the full-length protocol
Consent or assent	26a	Who will obtain informed consent or assent from potential trial participants or authorised surrogates, and how (see Item 32)	Section 12.5.5 of the full-length protocol
	26b	Additional consent provisions for collection and use of participant data and biological specimens in ancillary studies, if applicable	Section 12.5.5 of the full-length protocol
Confidentiality	27	How personal information about potential and enrolled participants will be collected, shared, and maintained in order to protect confidentiality before, during, and after the trial	Section 12.5.6 of the full-length protocol
Declaration of interests	28	Financial and other competing interests for principal investigators for the overall trial and each study site	Section 13.2 of the full-length protocol
Access to data	29	Statement of who will have access to the final trial dataset, and disclosure of contractual agreements that limit such access for investigators	Section 12.3 of the full-length protocol
Ancillary and post-trial care	30	Provisions, if any, for ancillary and post-trial care, and for compensation to those who suffer harm from trial participation	Page 16
Dissemination policy	31a	Plans for investigators and sponsor to communicate trial results to participants, healthcare professionals, the public, and other relevant groups (eg, via publication, reporting in results databases, or other data sharing arrangements), including any publication restrictions	Page 25

For peer review only - http://bmjopen.bmj.com/site/about/guidelines.xhtml

	31b	Authorship eligibility guidelines and any intended use of professional writers	Page 25
	31c	Plans, if any, for granting public access to the full protocol, participant-level dataset, and statistical code	Page 25
**Appendices**			
Informed consent materials	32	Model consent form and other related documentation given to participants and authorised surrogates	Appendix to the original protocol
Biological specimens	33	Plans for collection, laboratory evaluation, and storage of biological specimens for genetic or molecular analysis in the current trial and for future use in ancillary studies, if applicable	Appendices to the original protocol

*It is strongly recommended that this checklist be read in conjunction with the SPIRIT 2013 Explanation & Elaboration for important clarification on the items. Amendments to the protocol should be tracked and dated. The SPIRIT checklist is copyrighted by the SPIRIT Group under the Creative Commons "Attribution-NonCommercial-NoDerivs 3.0 Unported" license.

For peer review only

BMJ Open

Study protocol for THINK: A multinational open-label Phase I study to assess the safety and clinical activity of multiple administrations of NKR-2 in patients with different metastatic tumour types

Journal:	*BMJ Open*
Manuscript ID	bmjopen-2017-017075.R2
Article Type:	Protocol
Date Submitted by the Author:	26-Sep-2017
Complete List of Authors:	Lonez, Caroline; Celyad SA,<br>Verma, Bikash; Celyad SA<br>Hendlisz, Alain; Institut Jules Bordet, Medical Oncology Department-GastroIntestinal Unit<br>Aftimos, Philippe; Institut Jules Bordet, Medical Oncology<br>Awada, Ahmad; Institut Jules Bordet, Medical Oncology<br>Van Den Neste, Eric; Universite Catholique de Louvain La Faculte de Medecine<br>Catala, Gaetan; Universite Catholique de Louvain La Faculte de Medecine<br>Machiels, Jean-Pascal; Universite Catholique de Louvain La Faculte de Medecine<br>Piette, Fanny; IDDI SA<br>Brayer, Jason; H Lee Moffitt Cancer Center and Research Institute<br>Sallman, David; H Lee Moffitt Cancer Center and Research Institute<br>Kerre, Tessa; Ghent University Hospital<br>Odunsi, Kunle; Roswell Park Cancer Institute<br>Davila, Marco; H Lee Moffitt Cancer Center and Research Institute; USF Health Morsani College of Medicine<br>Gilham, David; Celyad SA<br>Lehmann, Frédéric; Celyad SA
**Primary Subject Heading**:	Oncology
Secondary Subject Heading:	Immunology (including allergy)
Keywords:	NKG2D, chimeric antigen receptor, CAR-T, solid tumours, NKR-2, multiple injections

SCHOLARONE™
Manuscripts

Study protocol for THINK: A multinational open-label Phase I study to assess the safety and clinical activity of multiple administrations of NKR-2 in patients with different metastatic tumour types

Authors and affiliations

Caroline Lonez[1], Bikash Verma[1], Alain Hendlisz[2], Philippe Aftimos[2], Ahmad Awada[2], Eric Van Den Neste[3], Gaetan Catala[3], Jean-Pascal H. Machiels[3], Fanny Piette[4], Jason B. Brayer[5], David A Sallman[5], Tessa Kerre[6], Kunle Odunsi[7], Marco L Davila[5,8], David E Gilham[1], Frédéric F Lehmann[1]

1. Celyad S.A., Mont-Saint-Guibert, Belgium; 2. Institut Jules Bordet, Université Libre de Bruxelles, Brussels, Belgium; 3. Cliniques Universitaires Saint-Luc, Université Catholique de Louvain, Brussels, Belgium; 4. International Drug Development Institute, Louvain-la-Neuve, Belgium; 5. H. Lee Moffitt Cancer Center, Tampa, FL, USA; 6. Ghent University Hospital, Ghent, Belgium; 7. Roswell Park Cancer Institute, Buffalo, NY, USA; 8. Morsani College of Medicine, University of South Florida, Tampa, FL, USA. Correspondence to Dr. Caroline Lonez, Celyad SA, Rue Edouard Belin 2, 1435 Mont-Saint-Guibert, Belgium. Email: clonez@celyad.com. Phone: +32(0)10394182.

Keywords

NKG2D, chimeric antigen receptor, CAR-T, solid tumours, NKR-2, multiple injections

Word count: 7150

For peer review only - http://bmjopen.bmj.com/site/about/guidelines.xhtml

Abstract

Introduction: NKR-2 are autologous T-cells genetically modified to express a chimeric antigen receptor (CAR) comprising a fusion of the NKG2D receptor with the CD3ζ signalling domain, which associates with the adaptor molecule DAP10 to provide co-stimulatory signal upon ligand binding. NKG2D binds eight different ligands expressed on the cell surface of many tumour cells and which are normally absent on non-neoplastic cells. In preclinical studies, NKR-2 demonstrated long-term anti-tumour activity towards a breadth of tumour indications, with maximum efficacy observed after multiple NKR-2 administrations. Importantly, NKR-2 targeted not only tumour cells but also tumour neovasculature and the local tumour immunosuppressive microenvironment and this mechanism of action of NKR-2 was established in the absence of pre-conditioning.

Methods and analysis: This open-label Phase I study will assess the safety and clinical activity of NKR-2 treatment administered three times, with a two-week interval between each administration, in different tumour types. The study will contain two consecutive segments: a dose escalation phase followed by an expansion phase. The dose escalation study involves two arms, one in solid tumours (5 specific indications) and one in haematological tumours (2 specific indications), and will include 3 dose-levels in each arm: $3x10^{8}$, $1x10^{9}$ and $3x10^{9}$ NKR-2 per injection. Upon the identification of the recommended dose in the first segment, based on dose-limiting toxicity occurrences, the study will expand to seven different cohorts examining the seven different tumour types separately. Clinical responses will be determined according to standard RECIST criteria for solid tumours, or international working group response criteria in haematological tumours.

For peer review only - http://bmjopen.bmj.com/site/about/guidelines.xhtml

Ethics approval and dissemination: Ethical approval has been obtained at all sites. Written informed consent will be taken from all participants. Results of this study will be disseminated through presentation at international scientific conferences and reported in peer-reviewed scientific journals.

Trial registration: NCT03018405, EudraCT 2016-003312-12.

Strengths and limitations of this study

- Standard dose escalation phase and an expansion phase to investigate for the first time the safety and initial clinical activity of NKR-2 (NKG2D-based chimeric antigen receptor (CAR)-T cells retrovirally modified) in a multiple injection schedule.
- Phase I study exploiting the multiple ligand targeting capability of human NKG2D by assessing the safety and initial clinical activity of NKR-2 against a broad range of tumour indications including haematological and solid tumour types.
- Based on the unique NKR-2 mode of action, NKR-2 will be administered without any prior pre-conditioning.

For peer review only - http://bmjopen.bmj.com/site/about/guidelines.xhtml

Introduction

CAR T-cell therapy

The Chimeric Antigen Receptor (CAR) is an artificial construct that endows T-cells with pre-determined specificity [1]. Prior to adoptive transfer they have to be expanded *in vitro* to reach therapeutically sufficient numbers. In this manner, large numbers of tumour antigen specific T-cells can be rapidly generated for use in adoptive cell therapy. The CAR itself has developed through several generations but has largely followed the configuration of extracellular antigen binding domain (most commonly a single-chain variable fragment (scFv) derived from an antibody), a transmembrane domain and intracellular signalling domains. At present, second generation formats tend to predominate featuring a co-stimulatory signalling domain (*e.g.* CD28, 4-1BB) fused to a T-cell activating domain (*e.g.* CD3ζ) [1, 2].

The CD19 specific CAR provides the paradigm against which other CAR approaches are currently measured due to the impressive clinical responses seen in early phase clinical trials targeting B-cell malignancies. Reports of initial objective clinical responses in 70-90% of patients with advanced chemo-resistant B-cell leukaemia have fuelled interest in the area with the number of CD19-targeted CAR T-cell trials rapidly rising within the last few years [3, 4]. The CD19-CAR T-cell therapy approach is based on a "*transplant procedure-like*" approach, *i.e.*, a single CAR T-cell infusion in pre-conditioned (lymphodepleted) patients and for which persistence, expansion and survival of the transferred T-cells are crucial [3, 4]. All pre-conditioning regimens follow a similar concept where there is a transient depletion of circulating lymphoid cell populations to provide 'space' for the adoptively transferred T cells to occupy [5, 6]. Additional beneficial effects of pre-conditioning include the production of homeostatic cytokines and dampening of suppressive factors such as regulatory T-cells thereby providing a window of immunologic opportunity to target the tumour.

For peer review only - http://bmjopen.bmj.com/site/about/guidelines.xhtml

However, single-target therapy may select for and spur escape variants by clonal selection or antigen loss. Several CD19-specific therapies indeed evidenced cases of relapse resulting from CD19 antigen loss in leukaemia cells post-CD19-specific CAR T-cell therapy [7-10]. Furthermore, these therapies are also associated with significant levels of toxicity [11, 12] including cytokine release syndrome (CRS), neurotoxicity and on-target off-tumour toxicity against normal cells (*e.g.* CD19-specific CAR T-cells also target normal B-cells expressing CD19, causing prolonged B-cell aplasia and hypogammaglobulinemia [13, 14]). Moreover, anaphylaxis reaction to the foreign components of the CAR (*e.g.* most CARs use scFv from murine origin which might potentially induce transgene-specific humoral and cellular immune responses against the murine domain [15-17]) will impact drastically the safety of multiple injection schedule [18]. The risk/benefit is clearly biased towards benefit since these patients have highly advanced cancer with little, if any, alternative effective therapeutic option. However, this toxicity does provide a level of patient selection that impacts recruitment onto current trials and the wider applicability for the general patient population.

By contrast, outside of the B-cell malignancy setting, the CAR T-cell approach has met with much reduced levels of clinical success [2]. Notably, apart from CD19, there are only a few examples of normal cell lineages that can be safely eliminated along with malignant cells that share the targeted antigen, even within haematological malignancies. Targeting an antigen on tumour cells that is also present on normal cells, *e.g.* on the myeloid lineage, could lead to on-target off-tumour toxicity and serious side effects related to therapy [19]. Early studies targeting solid tumours reported little evidence of clinical effect while there was some evidence of on-target, off-tumour toxicity seen using CAR targeting carbonic anhydrase-IX [16, 20] in renal cell carcinoma or Her2/neu-specific CAR-Ts in colorectal cancer [21]. Investigators are therefore still seeking for the ideal target antigen that should be highly

For peer review only

For peer review only - http://bmjopen.bmj.com/site/about/guidelines.xhtml

overexpressed on tumour cells as compared with normal cells. Whilst some recent studies have shown limited clinical effect but nowhere near to that seen with CD19 CAR T-cell therapy [3, 22], the challenges posed by solid tumours are numerous and have been extensively reviewed recently [23-26]. In brief, these include the on-target off-tumours impact related to expression of the target on normal tissues, the need for trafficking into the tumour microenvironment and tumour stroma, tumour heterogeneity and the requirement to overcome the hostile immune suppressive tumour microenvironment, potentially leading to CAR-T exhaustion, clearly requiring the further development of the basic CAR approach [27]. Several options including third and fourth generation CARs (*e.g.* Armoured CAR, TRUCKs), logic gated activation strategies or the combination of CAR-Ts with T-cell checkpoint inhibitors are currently under development to solve these problems.

NKR-2

NKR-2 are autologous T-cells genetically modified to express a CAR comprising a fusion of the full-length Natural Killer Group 2D (NKG2D) receptor with the CD3ζ signalling domain. NKG2D is a C-type, lectin-like, type II transmembrane glycoprotein activating receptor expressed in humans on Natural Killer (NK), Natural Killer T, activated $CD8^+$ T-cells and, under certain conditions, some $CD4^+$ and γδ+ T-cell subsets [28] and functioning to target and kill cancer or infected cells. In humans, NKG2D is expressed as a homodimer that associates with the adaptor molecule DNAX-activating protein of 10 kDa (DAP10) to form a hexameric structure [29]. The interaction with DAP10 is essential to stabilize the surface expression of NKG2D in T-cells and to mediate signal transduction and cellular activation upon ligand recognition [30]. Therefore, whilst the NKR-2 CAR format suggests a 'first generation' configuration, the NKG2D structural component, via its interaction with endogenous DAP10, engages native T-cell costimulatory signalling apparatus to providing a sufficient co-

For peer review only - http://bmjopen.bmj.com/site/about/guidelines.xhtml

stimulatory signal which complement the primary CD3ζ vector-derived signal, indicating that the NKR-2 actually works rather like a 'second generation' CAR (Figure 1). Furthermore, the requirement for endogenous DAP10 expression for stable expression of NKG2D within cells defines an additional embedded safety measure to restrict NKR-2 vector expression and function specifically to cells expressing native DAP10 (*e.g.* T-cells).

In humans, eight NKG2D ligands have been identified, encoded by different loci on two different arms of the chromosome 6 and corresponding to two main families: the Major Histocompatibility Class (MHC) class I chain related proteins (MIC) which include MICA and MICB proteins, and the structurally diverse Unique Long 16 binding proteins (UL16-binding proteins or ULBP) which include ULBPs 1 to 6 [31]. The expression of these ligands can be induced by stress such as viral infection, oxidative or thermal stress, genotoxic drugs, DNA damaging agents, tissue damage, heat shock, inflamed tissues (*e.g.* autoimmune diseases) and malignant transformation [32]. Importantly, a broad range of primary tumours express NKG2D ligands [33, 34]. Whilst the NKG2D ligand expression profile is often heterogeneous, the large majority of tumour samples express at least one ligand (and often multiple ligands) including haematological and solid tumour types, implying that the profile of ligand expression is suitable to be exploited as a cancer therapy [34]. Finally, the surface expression of NKG2D ligands can be modulated by proteolytic cleavage with the release of the soluble ectodomain [35, 36]. These shed NKG2D ligands may down-modulate NKG2D expression on NK cells and T-cells, contributing to tumour immune escape and elevated serum levels were accordingly often associated with poor prognosis [35, 36].

The breadth of ligands targeted by NKG2D provides T-cells armed with the NKR-2 receptor the potential to challenge an extensive range of tumour indications [33, 34].

For peer review only - http://bmjopen.bmj.com/site/about/guidelines.xhtml

Table 1: Preclinical studies with NKR-2

	Targeted model	Publications	Main findings
In vitro experiments			
Murine equivalent of NKR-2	Lymphoma<br>Myeloma Ovarian cancer<br>Melanoma	[37, 38]<br>[39]<br>[40-42]<br>[43]	NKR-2 induces IFN-γ expression, cytokine release upon co-culture with tumour cell lines and trigger specific cytotoxicity against several tumour types
Human NKR-2	Myeloma<br>Tumour cell lines	[44]<br>[45, 46]	NKR-2 induces IFN-γ expression, cytokine release upon co-culture with tumour cell lines and trigger specific cytotoxicity against several tumour types
In vivo experiments			
Murine equivalent of NKR-2	Lymphoma-bearing C57BL/6 mice	[37, 38, 47]	Efficacy of single i.v. and s.c. administration of NKR-2 (1-7.5x10^6 cells/injection) and resistance to a re-challenge with same tumour type
		[47]	Efficacy against heterogeneous tumours
		[38]	Efficacy of multiple i.v. administration of NKR-2 (5x10^6 cells/injection)
		[48]	Single and multiple i.v. injections of NKR-2 (5x10^6 – 2x10^7 cells/injection) are not toxic at doses < 2x10^7 NKR-2 per injection.
	Multiple myeloma-bearing C57BL/ KaLwRij mice	[39]	Efficacy of single and multiple i.v. administration of NKR-2 (5x10^6 cells/injection) and resistance to a re-challenge with same tumour type<br>Effect of lymphodepletion<br>Host memory immune response<br>Low persistence of NKR-2 in the body after administration
	Ovarian tumour-bearing C57BL/6 mice	[40-42, 49-51]	Efficacy of single i.p. administration of NKR-2 (5x10^6 cells/injection) and resistance to a re-challenge with same tumour type
		[49, 51]	Efficacy against established tumours
		[41, 42, 50, 51]	Effect on tumour microenvironment<br>Host memory immune response
		[41, 42, 47, 49]	Efficacy of multiple i.p. administration of NKR-2 (5x10^6 cells/injection)
		[47]	Efficacy against heterogeneous tumours
	Melanoma-bearing C57BL/6 mice	[43]	Efficacy of multiple i.t. administration of NKR-2 (2x10^6 cells/injection)<br>Effect on tumour vasculature
	Healthy C57BL/6 mice	[48]	Single and multiple i.v. injections of NKR-2 (5x10^6 – 2x10^7 cells/injection) are not toxic at doses < 2x10^7 NKR-2 per injection.
Human NKR-2	Pancreatic tumour xenograft-bearing NOD-scid IL2Rgamma^{null} mice	[46]	Efficacy of multiple i.p. administration of NKR-2 (5x10^6 cells/injection)

Preclinical studies have shown the murine equivalent of NKR-2 (chNKG2D T-cells) can mediate potent anti-tumour activity against both haematological and solid tumours [52],

For peer review only - http://bmjopen.bmj.com/site/about/guidelines.xhtml

including highly heterogeneous tumours with less than 10% ligand-positive tumour cells (see Table 1 for a summary of these data)[47].

Critically, these pre-clinical data in multiple syngeneic tumour models indicate that anti-tumour efficacy was not dependent upon lymphodepleting pre-conditioning while the induction of a tumour specific adaptive immune response by NKR-2 therapy enabled the long-term protective effects of the approach [39]. Recent data also confirmed that human NKR-2 are effective *in vivo* in a mouse xenograft model of human pancreatic cancer [46]. Importantly, preclinical data suggest four different mechanisms of action of NKR-2, including *(i)* direct cytotoxicity against cancer cells, *(ii)* anti-angiogenic activity potentially through targeting of NKG2D ligands expressed upon tumour neovasculature [43], *(iii)* modulation of the immune tumour microenvironment through direct targeting of immunosuppressive myeloid-derived suppressor cells (MDSCs) and regulatory T-cells (Tregs), and recruitment of myeloid cells and activated macrophages [49, 51], together resulting in *(iv)* the observed generation of a tumour-specific adaptive immune response [38, 41, 42, 49-51, 53] (Figure 2). Preclinical data also demonstrated that NKR-2 rapidly disappeared from tissues by seven days after injection suggesting a short-term persistence of NKR-2 (without uncontrolled expansion), although being able to induce a long-lasting memory host immune response against tumour-specific antigens [39]. Finally, preclinical data demonstrated that NKR-2 remained functionally intact in the presence of soluble MICA at concentrations much higher than the physiological concentrations [45].

Expressing the NKR-2 CAR into T-cells therefore allows the NKR-2 cells to present the broad specificity characteristic of NK cells whilst exploiting the migration, expansion, long half-life and potential to generate immunological memory characteristic of T-cells. In addition

For peer review only - http://bmjopen.bmj.com/site/about/guidelines.xhtml

to direct recognition of tumour cells, targeting of ligands expressed on the stroma of the tumour microenvironment can also lead to disruption of the essential support mechanisms required for tumour cell survival and growth. Moreover, in contrast to other single or limited target CAR-T therapies, the fact that NKR-2 targets several ligands from two main families putatively decreases the probability of target loss and associated relapse after treatment. Further, the lack of important *in vivo* cell expansion and long-term survival of NKR-2, and the induction of tumour specific adaptive immunity in the absence of patient pre-conditioning, also bears a safety-advantage preventing side effects generally associated with rapid CAR-T expansion [54] which often require development of strategies like embedded suicide systems or use of chemotherapy to eradicate the CAR T-cells [55, 56]. Collectively, this suggests that exploiting the targeting of NKG2D ligands by NKR-2 to fight against diverse tumour indications with a single generic construct combining two features of innate and adaptive immunity, provides potentially a new paradigm for CAR T-cell therapy that the current protocol is designed to investigate.

Methods and Analysis

Study objectives

The THINK (**TH**erapeutic **I**mmunotherapy with **NK**R-2) trial is an open-label Phase I study which primarily aims to assess the safety and clinical activity of the NKR-2 treatment administered three times every 2 weeks between each administration in different tumour types. The study is split into two segments each with specific study objectives. The first dose escalation segment focuses upon determining the recommended dose of NKR-2 cells for second segment of THINK. Segment 2 extends the safety study and also investigates initial clinical activity of NKR-2 across multiple tumour indications. In both segments, correlative measures of clinical activity will be examined.

For peer review only - http://bmjopen.bmj.com/site/about/guidelines.xhtml

Study drug

NKR-2 refers to the viable autologous $CD3^+$ cells obtained after transduction of autologous T-cells with the NKG2D-based CAR, which contains a total viable population of at least 80% $CD3^+$ cells, with at least 50% $CD3^+$ being transduced. NKR-2 will be supplied cryopreserved in bags containing a T-cell dose in accordance with the dose-level which is to be administered.

Rationale for the NKR-2 dose and regimen

NKR-2 has been used in the single infusion dose escalation clinical study CM-CS1 (NCT02203825) which evaluated the safety and clinical activity NKR-2 administration without prior lymphodepleting conditioning in patients with two distinct haematological malignancies: Acute Myeloid Leukaemia (AML)/ Higher Risk Myelodysplastic Syndrome (MDS) refractory anaemia with excess blasts, that are not in remission and for which standard therapy options are not available, and relapsed or refractory progressive Multiple Myeloma (MM) after several lines of standard therapy. The trial has been successfully completed with no observed dose limiting toxicities at the highest dose infused ($3x10^7$ total cells) and initial signs of clinical benefit [57].

In preclinical models, the pharmacologically effective dose (PED) of the murine NKR-2 ranged from $1x10^6$ cells to $7.5x10^6$ cells per dose (intravenous (i.v.) single injection). Although a significant anti-tumour activity was obtained with a single injection of murine NKR-2, 100% overall survival and complete recovery was obtained following repeated three sequential doses of $5x10^6$ cells/mouse without any adverse effect [38, 39, 41, 49]. Moreover, a treatment regimen with two weeks' interval between injections was far more successful in terms of activity as compared to a weekly interval demonstrating that successful treatment of

For peer review only - http://bmjopen.bmj.com/site/about/guidelines.xhtml

established tumours did not require the administration of more NKR-2 [49]. The maximum tolerated dose (MTD) after a single i.v. injection was determined to be $1x10^7$ cells, and repeated administration of this dosage to healthy and tumour-bearing mice did not produce any major toxicities [48].

Following these values of PED and MTD, the no-observed effect level (NOEL) was defined at $5x10^6$ cells/injection per mouse and were identical whatever the tumour type model (haematological or solid tumour). Using the allometry scaling exponent of 0.75, as defined by FDA guidelines (USFDA, 2005), human-equivalent PED and MTD were therefore estimated at 0.43 - $3.2x10^9$ and $4.3x10^9$ cells/injection, respectively. Together these observations of high tolerability in initial single dose phase I clinical trial coupled with pre-clinical efficacy experimentation strongly support the concept of a dose escalation segment of $3x10^8$ to $3x10^9$ NKR-2 per infusion given three times each separated by a 14-day interval.

Patient population

Refractory or relapsing patients with metastatic or locally advanced colorectal cancer (CRC), urothelial carcinoma, triple-negative breast cancer (TNBC), pancreatic cancer, recurrent epithelial ovarian and fallopian tube carcinoma, acute myeloid leukaemia AML/MDS and MM, which failed from standard treatments, will be recruited into this protocol (the key inclusion and exclusion criteria are presented in Table 2).

The study will enrol patients in different countries in Europe and in USA with a dose escalation segment open to a restricted academic network in Belgium and USA. List of study sites can be obtained on clinicaltrials.gov (NCT03018405).

For peer review only - http://bmjopen.bmj.com/site/about/guidelines.xhtml

Table 2: Key eligibility criteria

Main inclusion criteria:

- Men or women ≥ 18 years old at the time of signing the ICF,
- Patient with specific cancer indications (see below),
- Disease must be measurable according to the corresponding guidelines,
- ECOG performance status of 0 or 1 or 2 based on peripheral neuropathy from prior therapies,
- Patient with adequate bone marrow reserve, hepatic and renal functions,
- Left ventricular ejection fraction of > 40%
- For patients with solid tumour type, the patient must agree to have a tumour biopsy at baseline.

Main exclusion criteria:

- Patient with a tumour metastasis in the central nervous system,
- Patients who have received another cancer therapy within 2 weeks before the planned day for the apheresis,
- Patients who receive or are planned to receive any other investigational product within the 3 weeks before the planned day for the first NKR-2 administration,
- Patients who are planned to receive concurrent growth factor, systemic steroid or other immunosuppressive therapy or cytotoxic agent,
- Patients who underwent major surgery within 4 weeks before the planned day for the first NKR-2 administration,
- Patients who have active infections necessitating use of antibiotics/antivirals treatment,
- Patients with history of autoimmune disease.

Disease-specific inclusion criteria

Patients must have either:

- A documented metastatic or locally advanced colorectal adenocarcinoma and having received, being intolerant, or having refused at least 2 prior standard cancer therapy regimens as part of their primary treatment regimen or part of their treatment for management of recurrent/persistent disease,
- A documented recurrent epithelial ovarian cancer or fallopian tube carcinoma and having received or refused at least 2 prior standard cancer chemotherapy regimens as part of their primary treatment regimen or part of their treatment for management of recurrent/persistent disease,
- An inoperable locally advanced or metastatic urothelial carcinoma that have progressed after previous platinum-based chemotherapy,
- A metastatic or locally advanced triple-negative breast cancer (TNBC) and having received at least 1 prior cancer therapy regimen as part of their treatment for management of recurrent/persistent disease,
- A metastatic or locally advanced pancreatic ductal adenocarcinoma and having received, being intolerant or having refused at maximum 2 prior lines of standard cancer chemotherapy regimens,
- Relapsed or refractory AML (*i.e.* ≥ 5% blasts in bone marrow or in peripheral blood) after one prior therapy or patients with myelodysplastic syndrome (*i.e.* ≥ 5% blasts in bone marrow or ≥ 2% blasts in peripheral blood) or MDS with TP53 mutation who failed from prior treatments,
- Relapsing or refractory/relapsing MM with a minimum of 1 prior line of systemic therapy

For peer review only

The tumour types targeted in this protocol were selected primarily based on their high expression of the NKG2D ligands (with a prevalence of 78-100% tumour samples expressing at least 1 ligand) [33, 34]. Furthermore, NKG2D ligand expression is a dynamic and context-dependent process, quickly varying over time in response to cytokines in the tumour microenvironment and evolving along with tumour progression, local inflammatory responses

For peer review only - http://bmjopen.bmj.com/site/about/guidelines.xhtml

and treatments [33, 34], hence difficult to assess. Finally, even if NKG2D ligand expression is heterogeneous with respect to the exact ligands found, based on preclinical data, potential therapeutic benefit might be achieved in patients with highly heterogeneous tumours [47] or even tumours considered as "negative" for NKG2D ligands because of a NKG2D ligand positive tumour microenvironment and/or neovasculature and/or activation of host immune system [38, 41, 42, 49-51, 53]. Thus, no eligibility criteria were based upon the individual expression profile of NKG2D ligands and no diagnostic test for ligand expression used for recruitment into this study. Correlative studies, however, will be carried out during the trial and will attempt to investigate the expression profile of patient tumours with clinical response.

THINK study design

THINK Phase I study will contain two consecutive segments: a dose escalation and an expansion segment (Figure 3).

The **dose escalation** design will include 3 dose-levels of NKR-2. The assumption is made that the safety profile of the treatment could be different in haematological versus solid tumour types. Therefore, it will be assessed separately in two different arms, one in solid tumours (CRC, urothelial carcinoma, TNBC, pancreatic cancer or epithelial ovarian and fallopian tube carcinoma) and one in haematological tumours (AML/MDS or MM). Each arm will use a 3+3 design to determine the recommended dose (RecD) of the NKR-2 treatment for the corresponding cohorts in the expansion segment, as based on the occurrence of dose-limiting toxicities (DLTs) (*i.e.* 3 patients will be evaluated per dose-level for each arm of the segment and 3 additional patients will be added if 1 out of 3 patients in this cohort experiences a DLT, to further assess the safety of the treatment). The sample size of the dose escalation part will

For peer review only - http://bmjopen.bmj.com/site/about/guidelines.xhtml

be between 2 and 18 patients per arm. Six patients will be treated at the MTD (or highest dose-level if no DLT was observed).

The RecD will be the maximum tolerated dose (MTD) unless in case no MTD is determined in the dose escalation segment of the study. In the latter, the RecD will be highest dose evaluated in the dose-escalation segment.

The RecD will be further evaluated in the **expansion segment** of the study to assess separately the safety profile and initial clinical activity of the NKR-2 treatment in 7 cohorts of patients with the distinct tumour types (up to 14 patients per tumour type). The expansion segment in haematological and solid tumours will be initiated as soon as their respective dose escalation arm defines the RecD. During this segment, the RecD can still be adapted according to predefined safety rules in any specific tumour type. The statistical analysis is planned in 3 steps. A futility analysis will be conducted separately in the first 14 patients with a solid tumour and the first 6 patients with a haematological tumour type. These number of patients have been defined to have a 0.05 probability of finding 0 objective response at this step if the true probability of response were 0.2 (solid) or 0.4 (haematological). Subsequently, futility (no response in the first 7 patients) and efficacy (3 or more responses in 14 patients) will be tested by cohort, based on the Simon's two-stage optimal design. The assumptions are: type I error rate = 0.15, power = 0.80, response probability of poor drug = 0.10 / beneficial drug = 0.30. In total, up to 86 patients are expected to be enrolled in the expansion segment (in addition, the 6 patients from each arm of the dose escalation segment who were treated at the RecD will be included in these expansion analyses).

Treatment regimen

For peer review only - http://bmjopen.bmj.com/site/about/guidelines.xhtml

Three NKR-2 dose-levels will be evaluated during this study, *i.e.* $3x10^8$, $1x10^9$ and $3x10^9$ NKR-2 for each injection (adjusted to $4.6x10^6$, $1.5x10^7$ and $4.6x10^7$ NKR-2/kg, respectively for patients with body weight below or equal to 65 kg) with a schedule of administration of three NKR-2 doses administered with a 2-week interval per patient. No systemic lymphodepleting pre-conditioning will be performed prior to NKR-2 injections.

Trial Endpoints

The *primary endpoint* of the **dose escalation** segment will be the occurrence of dose limiting toxicities (DLTs) in all patients during the study treatment until 14 days after the last study treatment administration. *Secondary endpoints* will include the *(i)* occurrence of adverse events (AEs) and serious adverse events (SAEs) during the study treatment, *(ii)* occurrence of objective clinical response at D57, M3, M6, M9, M18 and M24 after 1st NKR-2 administration and according international guidelines, *(iii)* progression-free survival (PFS) and overall survival (OS), and duration of response for patients with objective response up to two years after first NKR-2 administration.

The *Primary endpoint* of the **expansion segment** will be the objective response rate (ORR) at D57, M3, M6, M9, M18 and M24 after first NKR-2 administration. *Secondary endpoints* will include the *(i)* PFS and OS, *(ii)* duration of response for patients with objective response up to two years after first NKR-2 administration and *(iii)* occurrence of AEs and SAEs and DLTs during the study treatment.

The overall aim of this segment is to understand the safety profile and develop initial observations of clinical activity in a cohort of up to 14 patients that support a deeper assessment of clinical efficacy of NKR2 in larger patient cohorts within a Phase II clinical study.

For peer review only - http://bmjopen.bmj.com/site/about/guidelines.xhtml

For peer review only

Response to treatment will be assessed according to standard RECIST criteria (version 1.1) for solid tumours or international recommendations for response criteria in haematological tumours. Apart from the visits scheduled per protocol, tumour assessment might also be performed at any time if considered as needed per investigator's decision (*e.g.* progression suspicion) and in regard to the RECIST 1.1. response confirmation.

Study duration

For each patient, the overall study duration will be 15 years. The study consists of a screening phase, a treatment administration phase, a treatment follow-up phase and a long-term safety follow-up (Figure 4). The duration of the administration phase will be 8 weeks, from Visit D1 to Visit D57. The duration of the treatment follow-up phase will be 22 months, from the end of the administration phase on D57 (Week 8) to Visit M24.

As per regulatory requirements, patients who received at least one NKR-2 treatment will need to be followed annually for a period of 15 years. Therefore, patients will also follow a **long-term safety follow-up** that will last for up to 15 years after the day of the first NKR-2 administration to collect safety data for 13 additional years. The surveillance will mimic as much as possible the standard follow-up of the respective tumour type including monitoring of important safety parameters.

Patients may be withdrawn from the study treatment or from the study at any time at the patients' discretion or at their investigator's discretion. The investigator will document on the patient's record whether the decision to discontinue treatment and/or study participation was made by the patient or the investigator and which reason applies.

For peer review only - http://bmjopen.bmj.com/site/about/guidelines.xhtml

In case of absence of disease progression at M24, PFS and OS as clinical endpoints will be followed-up on a yearly basis or at *ad hoc basis*, *i.e.* in case of progression/relapse suspicion. In case of disease progression at M24 or at any time before, the patient will be still asked to attend simplified follow-up procedures which will include (i) record of survival status, (ii) medication status and (iii) blood sampling for viral copy number (VCN) and replication competent retrovirus (RCR) assessment. Clinical progression at the end of the treatment phase (D57) will not to be considered as a criterion for withdrawal from the study, and will allow the continuation of the active follow up of the patient with or without the initiation of a new anti-cancer therapy.

Enrolment rules

There is no requirement for specific recruitment of each individual tumour indications to corresponding arm within the **dose escalation phase**, but each dose-level should enrol at least two different types of cancer. In each arm, patients will be enrolled at the next dose-level when all evaluable patients at the same dose-level have completed the safety period of 14 days after last NKR-2 injection. Within each dose-level, patients will be enrolled as soon as the previous patient has received at least one NKR-2 administration and has completed the 14-day safety period after the 1st injection.

Enrolment in the **expansion segment** of the study will be performed in 3 steps, with a statistical analysis at the end of each step (see next section). Within each step, all patients can theoretically be enrolled at the same time.

Approximately 15 centers are expected to be initiated for patient enrollment in the expansion segment. Recruitment is anticipated to be completed within approximately 24 months. If this target is not met, then the recruitment period may be extended accordingly.

For peer review only - http://bmjopen.bmj.com/site/about/guidelines.xhtml

Adverse events, serious adverse events and dose-limiting toxicities

All AEs starting from registration to 28 days after the last study treatment (Visit D57) must be recorded into the Adverse Event form in the patient's electronic case report form (eCRF), irrespective of intensity or causality with study treatment.

To ensure patient safety, every SAE, regardless of suspected causality, occurring after the patient enrollment and until the administration phase concluding visit (at D57) must be reported to the Sponsor within 24 hours of learning of its occurrence. Any SAEs experienced outside this period will only be reported to the Sponsor if the investigator suspects a causal relationship to the study participation or if a fatal SAEs occur after signature of the ICF.

Dose-limiting toxicity (DLT) refers to a toxicity that is experienced during treatment and within 14 days following any NKR-2 dose, is new and at least possibly related to study treatment. This includes any Grade 3 or higher toxicity or Grade 2 or higher autoimmune toxicity that cannot be controlled to Grade 1 or less within 72 hours with appropriate treatment, with the exception of Fever grade 3 and immediate hypersensitivity reactions occurring within 2 hours of cell infusion that are reversible to a grade 2 or less within 24 hours of cell administration with standard therapy.

Whenever a patient experiences toxicity that fulfills the criteria for a DLT, the study treatment will be interrupted. The standard time period for collecting and recording DLTs will be 14 days after each study treatment administration. AEs/SAEs who meet the definition of DLTs but that are reported outside of this time window will be reported as AEs/SAEs but not as DLTs.

For peer review only - http://bmjopen.bmj.com/site/about/guidelines.xhtml

The standard time period for collecting and recording pregnancy will begin at the first receipt of the product and last until the end of the study.

In addition to the above-mentioned reporting requirements and in order to fulfill international reporting obligations, SAEs that are related to study participation (e.g., protocol-mandated procedures, invasive tests, a change from existing therapy) will be collected and recorded from the time the patient consents to participate in the study until she/he is discharged.

Conduct of analysis and statistical considerations

All statistical analyses will be performed using SAS and/or StatXact software. The results will be reported per dose-level/tumour type cohort and overall.

Demographic characteristics (age, gender and race) physical examination, medical history and baseline tumor characteristics will be tabulated and analyzed with appropriate descriptive statistics on the *total treated population.*

The **analysis of safety** will be performed on the *total treated population*. All the AEs will be graded according to the NCI-CTCAE, Version 4.03 and will be coded to the preferred term level using the Medical Dictionary for Regulatory Activities (MedDRA). A summary of AEs by maximum grade and cohort. The same analysis will be presented for SAEs and events with a suspected relationship to the study treatment. A summary of AEs by maximum grade and cohort per treatment dose will also be performed. SAEs, DLTs and discontinuation due to adverse events(s) will be described in detail by patient narratives.

A summary of the concomitant medications, classified by ATC term, will be displayed by cohort. Individual listing of concomitant medications per patient and per timepoint will also

For peer review only - http://bmjopen.bmj.com/site/about/guidelines.xhtml

be provided. Finally, the total number of study treatments administered to each patient and the length of active follow-up will be summarized.

The **analysis of clinical activity** will be performed on the *total treated population i.e.* all patients which received at least one NKR-2 treatment (in case of patient's withdrawal prior the end of the administration phase). Sensitivity analyses related to the clinical activity endpoints will be performed on the *according to protocol* population which includes all evaluable patients who have complied with all the procedures defined in the protocol up to concluding visit.

The first ORR will be assessed at Visit D57. The occurrence and duration of objective clinical response (CR, PR and SD) will be reported. Disease progression, including death, during the study will be described. Estimates of the rates of best clinical response and their 95% CIs will be reported. Kaplan-Meier curves will be estimated for PFS and OS.

In addition, other exploratory analyses may be performed, *i.e.*, the clinical activity may be assessed in sub-populations based on the NKG2D ligands tumor expression, types of ligands expressed in tumor, serum levels of soluble NKG2D ligands, etc.

In the dose-escalation segment, safety data will be monitored closely on an ongoing basis. The main analysis (leading to the selection of the RecD) will be conducted separately in each arm once all patients in the last dose-level have received the 3 NKR-2 administrations and have completed the safety period (D43). All data related to the primary endpoints of the study will be analysed. The final analysis will be performed once all patients have attended the concluding visit of the follow-up phase (M24) or have been withdrawn from the study.

For peer review only - http://bmjopen.bmj.com/site/about/guidelines.xhtml

The expansion segment of the study will proceed in 3 steps, with a statistical analysis at the end of each step (Figure 3). These steps aim to stop the study for futility in specific cohorts or specific tumour types in case of no (sufficient) responders in the corresponding cohorts at the time of analysis.

An interim analysis for futility will be performed on the first 14 patients over all 5 cohorts of patients with solid tumour types and on the first 6 patients over the two cohorts of patients with haematological tumour types who have either completed the administration phase (D57) or have withdrawn from the study.

The second step consists in cohort futility analyses after the first 7 cancer patients in each tumour type have either completed the administration phase (D57) or have withdrawn from the study. At this step, an evaluation of the safety of the NKR-2 treatment in each cohort will also be performed on the first 6 patients who completed the 6-week treatment period (D43) or had a DLT. This will allow to adapt the RecD in any specific cohort (tumour type) according to both safety rules and futility analysis. Indeed, if, and as soon as, 2 or more DLTs have been reported within a specific cohort, and the criterion for futility was not reached, the next patients enrolled in this cohort will be treated at the next lower dose-level. In contrast, if no DLT has been reported within a specific cohort, and the criterion for futility was not reached at step 2, the next patients enrolled will be treated with the next higher dose-level.

Finally, the final evaluation of the objective clinical response rate will be evaluated once all the first 14 cancer patients in each cohort have either completed their administration period (D57) or have withdrawn from study due to disease progression or DLT. If, in any cohort, there are >2 out of 14 patients presenting an ORR, the study will be considered as successful in this tumour type.

For peer review only - http://bmjopen.bmj.com/site/about/guidelines.xhtml

Correlative studies

Peripheral blood samples, bone marrow aspirates from patients with haematological malignancies, as well as tumour biopsies from patients with solid malignancies, will be collected at regular time-points during the study (see Figure 4) and used to determine NKR-2 persistence post-infusion (using a viral copy number test) and assess the presence of replication competent retrovirus (RCR). Systemic cytokine levels in peripheral blood and levels of tumour-related biomarkers (in the serum, bone-marrow or urine samples) will be also assessed to determine whether correlates of NKR-2 activity can be identified. Evaluation and characterization of the NKR-2 mode of action, involving the characterization of the pre-existing and post/during-therapy humoral and cellular immune responses will be done on blood samples collected at baseline and post-NKR-2 infusion. Finally, the correlation with the tumour expression of NKG2D ligand in tumour tissues or cells (type and level) and NKR-2 activity, the characterization of the tumour microenvironment (TME), the NKR-2 infiltration and the modulation of the local tumour environment of this new type of T-cell therapy will be evaluated.

Toxicity Management

As previously mentioned, the use of CAR T-cells is limited by potentially severe toxicities including cytokine release syndrome (CRS), neurotoxicity and tumour lysis syndrome (TLS), on-target off-tumour toxicity (related to recognition of the target antigen on normal tissues), clonality and insertional oncogenesis, vector persistence and immunogenicity. While these events may have been seen in relation with other CAR-T therapies, and are provided for information, there are currently no events considered as expected for NKR-2 therapy (which can be different from other CAR-T studies based on the unique mode of action) and all

For peer review only - http://bmjopen.bmj.com/site/about/guidelines.xhtml

Serious Adverse Reactions (SARs) will be considered as suspected unexpected SARs (SUSARs).

The management of toxicities related to CAR-T therapies was described elsewhere [11, 12, 58] but is evolving with everyday new information available from ongoing studies. Sites will be kept informed all along the study for any new therapeutic options modifications or standardization between the different sites.

Briefly, CRS toxicity is currently of highest concern in the field. It is caused by a release of inflammatory cytokines such as IL-6, IL-2, IFN-γ, and TNF-α, following the recognition of the target antigen, resulting in a systemic inflammatory response similar to sepsis. Therefore, CRS will correlate with both efficacy and toxicity in patients receiving T cell-engaging therapies. Tocilizumab (anti IL-6) will be used as the first-line agent in the treatment of CRS. If the patient's condition does not improve or stabilize within 24 hours of the tocilizumab dose, administration of a second dose of tocilizumab and/or a second immunosuppressive agent (such as corticosteroids) will be considered, based on the CRS revised grading system created by Lee et al. [59] to identifying severity and implementation of optimal care strategies for CRS.

TLS resulting in renal insufficiency, rapidly rising uric acid level, or evidence of organ dysfunction will be managed with fluids and rasburicase as needed and as determined by the treating physicians.

Further, as NKG2D ligands may be upregulated upon stress signals, expression of ligands on non-cancer cells can be expected in tissues that are actively infected or inflamed or experienced cell stress of other aetiology. Infections could therefore invoke a significant

For peer review only - http://bmjopen.bmj.com/site/about/guidelines.xhtml

inflammatory response by NKR-2 by potentially inducing a cytokine release syndrome due to the increased recognition of ligands by NKR-2 cells. Therefore, any inflammation, infection, concurrent or previous treatment with anti-cancer therapies, will be carefully registered and any Grade 2 or higher autoimmune toxicity will be closely followed to detect any evidence of on-target off-tumour toxicity. Evidence of acute infection, as well as fever of 38°C/100°F or above, will be therefore considered as exclusion criteria at screening (see Table 2) or postponement criteria when observed during the treatment phase. In the latter, all study procedures and treatment administration will be resumed where it was left as soon as the patient's condition allows (within 4 weeks after the scheduled treatment date) or at the investigator's discretion after Steering Committee and Sponsor approvals.

Since NKR-2 construct consists entirely of human sequences, it is not expected to result in immunogenicity sometimes observed with other CAR-Ts using scFv from murine origin (see introduction), and therefore multiple NKR-2 injections are allowed without the anticipated risk of humoral immune responses.

Uncontrolled expansion and proliferation of the transformed cells increases the risks for onset of cytokine release syndrome and other CAR-associated toxicities. However, in contrast to more classical CAR-T therapies, NKR-2 demonstrated neither significant *in vivo* expansion nor long-term persistence until now [39], but since this is the first trial with NKR-2 administrated as multiple doses, the persistence of NKR-2 will be nevertheless carefully monitored post-infusion as mandatory exploratory research. If uncontrolled T-cell proliferation occurs (grade 3 or 4 toxicity related to NRK-2), and as it was reported that CAR associated toxicity respond to systemic corticosteroids, patients may be treated with

For peer review only - http://bmjopen.bmj.com/site/about/guidelines.xhtml

corticosteroids and/or chemotherapy to eradicate the CAR T-cells [60]. Patients will be treated with pulse, followed by a rapid taper.

Finally, following FDA recommendation for Retroviral and Lentiviral Vector Based Gene Therapy Products, the patient monitoring schedule will also include analysis of the replication-competent retrovirus (RCR) of patient samples up to 15 years after CAR-T injections.

Discussion

The THINK trial aims primarily to determine the safety and clinical activity of NKR-2 cell therapy, without pre-conditioning chemotherapy, in patients with advanced cancer. The dose escalation phase of the trial is designed to determine the recommended dose in haematological and solid tumours, which then is used in the expansion phase to assess clinical responses separately in seven different indications. Due to the multi-targeting specificity of NKR-2, the dose escalation and subsequent expansion segments are designed to facilitate a rapid assessment of NKR-2 activity across a range of tumours. This is advantageous since the assessment of safety and any evidence of clinical activity will be suitably evaluated across cancer types using a consistently produced cell product thereby avoiding issues that arise in multiple single tumour indication trials testing a single T-cell product. Interestingly, the number of ligands targeted by NKR-2 (8 ligands from 2 main families), combined with their stress-induced tissue expression, also limit the potential risk of clonal selection or antigen loss after injection, which has been observed for classical CAR-T constructs targeting a single antigen. This is expected to translate into limiting the risk for potential relapse.

For patients, the lack of pre-conditioning chemotherapy removes the need for an in-patient stay during the transient period of lymphodepletion translating to a likely improved patient

For peer review only - http://bmjopen.bmj.com/site/about/guidelines.xhtml

experience of the trial. Additionally, the absence of pre-conditioning also will reduce the potential toxicity of this treatment and facilitate providing a therapeutic solution to a larger patient population, including, notably, the elderly population.

The challenge facing classical CAR-T therapies in the human patient with advanced cancer (beyond B-cell malignancies) is major and, in the solid tumour situation, requires the cells to traffic to sites of tumour, overcome the highly suppressive tumour microenvironment and initiate direct tumour cell killing to drive a sustained anti-tumour response [23-25]. Based on the different intrinsic mechanisms of action of NKR-2 observed in preclinical experiments on various tumour models, NKR-2 should overcome, at least partially, the local immunosuppression and target expression heterogenicity in tumour tissues, while, because of its effect on tumour vasculature, trafficking into tumours might also be enhanced. Furthermore, the multiple infusion strategy was also found to increase activity and infiltration into tumours of other short-term persistent CAR T-cells (mesothelin-specific RNA CAR-T cells) [61], overcome the potential tumour-induced T-cell dysfunction upon interaction of CAR-T cells with the immunosuppressive tumour microenvironment [23], while being well tolerated in humans [62]. Consequently, as compared to classical CARs, clinical activity in solid tumours might be observed after treatment with NKR-2, without any construct modification nor combination with other immunotherapies. Since THINK is the first trial to evaluate the potential clinical activity of a multiple NKR-2 administration schedule in solid tumours, it offers a real opportunity as compared with classical CAR-Ts within the solid tumour type field, where therapeutic options are limited. Further impacting the tumor environment through combinations of chemotherapy or immune modulation will constitute subsequent steps to consider once we have progressed sufficiently in the THINK trial and demonstrated safety of NKR-2 administrations.

For peer review only - http://bmjopen.bmj.com/site/about/guidelines.xhtml

The multiple infusion strategy, absence of pre-conditioning chemotherapy, diversity of putative mechanisms of action, as well as the diversity of tumour types that can potentially targeted, therefore stand the NKR-2 CAR T-cell approach described in this protocol apart from the current 'gold standard' CAR T-cell therapy paradigm. In this context, the THINK trial provides a first approach of a global comprehensive clinical program embedding other phase I clinical protocols that will evaluate the combination of the NKR-2 with specific anti-cancer treatments and the loco-regional administration, among others.

Ethics and dissemination

The Investigator is responsible for ensuring the study is conducted in accordance with procedures and evaluations described in the clinical protocol. Deviations from the protocol shall not be made without prior approval by the Sponsor, except to treat a medical emergency and reduce immediate risk to the patient. In such a case, the Investigator shall immediately notify the Independent Ethics Committee (IEC) or Institutional Review Board (IRB) and the Sponsor, according to local requirements.

If changes to the protocol are deemed necessary, a written amendment should be prepared and signed by the Investigator and the Sponsor and approved by the IEC and applicable Competent Authorities prior to being implemented.

The **Steering Committee** will be composed of three members independent from investigators and the sponsor. The role of the Steering Committee will be to advise on events which may impact the general process of the clinical study. All safety data will be made available to the Steering Committee in a way that the Steering Committee can timely advise.

For peer review only - http://bmjopen.bmj.com/site/about/guidelines.xhtml

Celyad or designee will host specific **safety data review meetings** (or teleconferences) on a regular basis during the study to discuss and evaluate all of the gathered safety data. This safety follow-up will include teleconference at the end of each dose-level of the Phase I dose escalation arms and at any new relevant safety event onset (*i.e.*, DLT, treatment-related SAE, SUSAR). In addition, members of the Steering Committee will be requested to review the clinical study report(s) generated from data collected in this study.

Clinical data review meetings will be performed at the different study interim analyses for futility by the Celyad clinical trial team and the steering committee in specific. At the time of the interim analyses, the best overall response for each patient will be derived from the overall lesion response assessments recorded in the eCRF. This will be used to calculate the objective response rate in the respective group(s).

For the purpose of compliance with GCP and Competent Authorities guidelines it may be necessary for the Sponsor or a Competent Authority to conduct a site audit. This may occur at any time from start to after conclusion of the study. Therefore, when an investigator signs the protocol, he agrees to permit drug Competent Authorities and the Sponsor audits, providing direct access to source data/ documents.

Written **informed consent** will be taken from all participants. The patient will be asked to read and review the document. Upon reviewing the document, the investigator (or delegate) will explain the research study to the patient and answer any questions that may arise. The patients consent should be appropriately recorded by means of the patients personally dated signature prior to any procedures being done specifically for the study. The patients should have the opportunity to discuss the study with their surrogates or think about it prior to

For peer review only - http://bmjopen.bmj.com/site/about/guidelines.xhtml

agreeing to participate. The patient's participation is voluntary and the patient may withdraw consent at any time throughout the course of the study.

Patient confidentiality is strictly held in trust by the participating investigators, their staff, and the Sponsor(s) and their agents. This confidentiality is extended to cover testing of biological samples and genetic tests in addition to the clinical information relating to participating patients.

The study protocol, documentation, data and all other information generated will be held in strict confidence. No information concerning the study or the data will be released to any unauthorized third party, without prior written approval of the Sponsor.

The investigator remains accountable for the **study data**. Final dataset will be stored in a central database maintained by the Sponsor. At the conclusion of the study, the Sponsor will archive the study data in accordance with internal procedures. All information provided by the Sponsor and all data and information generated by the site as part of the study (other than a patient's medical records) remain the sole property of the Sponsor.

Results of this study will be disseminated through presentation at international scientific conferences and reported in peer-reviewed scientific journals.

The sponsor holds and will maintain an adequate **insurance policy** covering damages arising out of Celyad's sponsored clinical studies. Financial compensation to investigators and/or institutions will be in accordance with the agreement established between the investigator and/or institutions and the Sponsor.

The THINK trial (A Dose Escalation Phase I Study to Assess the Safety and Clinical Activity of Multiple Cancer Indications) is currently recruiting participants in Europe and USA.

For peer review only - http://bmjopen.bmj.com/site/about/guidelines.xhtml

Ethical approval has been obtained at all sites open for recruitment. The trial has been approved by ClinicalTrials.gov (Trial registration ID NCT03018405) and by EU Clinical Trials Register (EudraCT number: 2016-003312-12). The present publication refers to protocol version 2.0 (released the 10th of February 2017).

For peer review only

For peer review only - http://bmjopen.bmj.com/site/about/guidelines.xhtml

Abbreviations

AE: adverse event; AML: acute myeloid leukaemia; CAR: chimeric antigen receptor; chNKG2D: NKG2D-based CAR; CR: complete response; CRC: colorectal cancer; CRS: cytokine release syndrome; DAP10: DNAX-activating protein of 10 kDa; DLT: dose-limiting toxicity; MDS: myelodysplastic syndrome; MDSC: myeloid-derived suppressor cell; MHC: major histocompatibility complex; MICA/B: MHC class I chain related proteins A/B; MM: multiple myeloma; MTD: maximum tolerated dose; NK: natural killer; NKG2D: natural killer group 2D; NKR-2: human NKG2D receptor-based CAR T-cells; NOEL: no observed effect level; ORR: objective response rate; OS: overall survival; PBMC: peripheral blood mononuclear cell; PED: pharmacologically effective dose; PFS: progression-free survival; PR: partial response; RAEB: refractory anaemia with excess blasts; RCR: replication competent retrovirus; RecD: recommended dose for expansion segment; SAR: serious adverse reactions; SAE: serious adverse event; scFv: single-chain variable fragment; sMICA/B or sULBP1-6: serum level of MICA/B or ULBP1-6; SD: stable disease; SUSAR: suspected unexpected SAR; THINK: THerapeutic Immunotherapy with NKR-2; TLS: tumour lysis syndrome; TME: tumour microenvironment; TNBC: triple-negative breast cancer; Treg: regulatory T-cell; ULBP1-6: unique long 16 binding protein 1-6.

For peer review only

For peer review only - http://bmjopen.bmj.com/site/about/guidelines.xhtml

References

1. Fesnak AD, June CH, Levine BL. Engineered T cells: the promise and challenges of cancer immunotherapy. *Nat Rev Cancer* 2016;16(9):566-81.
2. Jackson HJ, Rafiq S, Brentjens RJ. Driving CAR T-cells forward. *Nat Rev Clin Oncol* 2016;13(6):370-83.
3. Dai H, Wang Y, Lu X, et al. Chimeric Antigen Receptors Modified T-Cells for Cancer Therapy. *J Natl Cancer Inst* 2016;108(7).
4. Gross G, Eshhar Z. Therapeutic Potential of T Cell Chimeric Antigen Receptors (CARs) in Cancer Treatment: Counteracting Off-Tumor Toxicities for Safe CAR T Cell Therapy. *Annu Rev Pharmacol Toxicol* 2016;56:59-83.
5. Pule MA, Savoldo B, Myers GD, et al. Virus-specific T cells engineered to coexpress tumor-specific receptors: persistence and antitumor activity in individuals with neuroblastoma. *Nat Med* 2008;14(11):1264-70.
6. Rosenberg SA, Yang JC, Sherry RM, et al. Durable complete responses in heavily pretreated patients with metastatic melanoma using T-cell transfer immunotherapy. *Clin Cancer Res* 2011;17(13):4550-7.
7. Maude SL, Frey N, Shaw PA, et al. Chimeric antigen receptor T cells for sustained remissions in leukemia. *N Engl J Med* 2014;371(16):1507-17.
8. Lee DW, Kochenderfer JN, Stetler-Stevenson M, et al. T cells expressing CD19 chimeric antigen receptors for acute lymphoblastic leukaemia in children and young adults: a phase 1 dose-escalation trial. *The Lancet* 2015;385(9967):517-28.
9. Grupp SA, Kalos M, Barrett D, et al. Chimeric antigen receptor-modified T cells for acute lymphoid leukemia. *N Engl J Med* 2013;368(16):1509-18.

For peer review only - http://bmjopen.bmj.com/site/about/guidelines.xhtml

10. Gardner R, Wu D, Cherian S, et al. Acquisition of a CD19-negative myeloid phenotype allows immune escape of MLL-rearranged B-ALL from CD19 CAR-T-cell therapy. *Blood* 2016;127(20):2406-10.
11. Bonifant CL, Jackson HJ, Brentjens RJ, et al. Toxicity and management in CAR T-cell therapy. *Mol Ther Oncolytics* 2016;3:16011.
12. Brudno JN, Kochenderfer JN. Toxicities of chimeric antigen receptor T cells: recognition and management. *Blood* 2016;127(26):3321-30.
13. Brentjens RJ, Riviere I, Park JH, et al. Safety and persistence of adoptively transferred autologous CD19-targeted T cells in patients with relapsed or chemotherapy refractory B-cell leukemias. *Blood* 2011;118(18):4817-28.
14. Kochenderfer JN, Dudley ME, Feldman SA, et al. B-cell depletion and remissions of malignancy along with cytokine-associated toxicity in a clinical trial of anti-CD19 chimeric-antigen-receptor-transduced T cells. *Blood* 2012;119(12):2709-20.
15. Kershaw MH, Westwood JA, Parker LL, et al. A phase I study on adoptive immunotherapy using gene-modified T cells for ovarian cancer. *Clin Cancer Res* 2006;12(20 Pt 1):6106-15.
16. Lamers CH, Willemsen R, van Elzakker P, et al. Immune responses to transgene and retroviral vector in patients treated with ex vivo-engineered T cells. *Blood* 2011;117(1):72-82.
17. Turtle CJ, Hanafi LA, Berger C, et al. CD19 CAR-T cells of defined CD4+:CD8+ composition in adult B cell ALL patients. *J Clin Invest* 2016;126(6):2123-38.
18. Maus MV, Haas AR, Beatty GL, et al. T cells expressing chimeric antigen receptors can cause anaphylaxis in humans. *Cancer Immunol Res* 2013;1(1):26-31.

For peer review only - http://bmjopen.bmj.com/site/about/guidelines.xhtml

19. Khalil DN, Smith EL, Brentjens RJ, et al. The future of cancer treatment: immunomodulation, CARs and combination immunotherapy. *Nat Rev Clin Oncol* 2016;13(5):273-90.

20. Lamers CH, Langeveld SC, Groot-van Ruijven CM, et al. Gene-modified T cells for adoptive immunotherapy of renal cell cancer maintain transgene-specific immune functions in vivo. *Cancer Immunol Immunother* 2007;56(12):1875-83.

21. Morgan RA, Yang JC, Kitano M, et al. Case report of a serious adverse event following the administration of T cells transduced with a chimeric antigen receptor recognizing ERBB2. *Mol Ther* 2010;18(4):843-51.

22. Beatty GL, O'Hara M. Chimeric antigen receptor-modified T cells for the treatment of solid tumors: Defining the challenges and next steps. *Pharmacol Ther* 2016;166:30-9.

23. Moon EK, Wang LC, Dolfi DV, et al. Multifactorial T-cell hypofunction that is reversible can limit the efficacy of chimeric antigen receptor-transduced human T cells in solid tumors. *Clin Cancer Res* 2014;20(16):4262-73.

24. Newick K, Moon E, Albelda SM. Chimeric antigen receptor T-cell therapy for solid tumors. *Mol Ther Oncolytics* 2016;3:16006.

25. Abken H. Adoptive therapy with CAR redirected T cells: the challenges in targeting solid tumors. *Immunotherapy* 2015;7(5):535-44.

26. Han S, Latchoumanin O, Wu G, et al. Recent clinical trials utilizing chimeric antigen receptor T cells therapies against solid tumors. *Cancer Lett* 2017;390:188-200.

27. Beatty GL, Moon EK. Chimeric antigen receptor T cells are vulnerable to immunosuppressive mechanisms present within the tumor microenvironment. *Oncoimmunology* 2014;3(11):e970027.

For peer review only - http://bmjopen.bmj.com/site/about/guidelines.xhtml

28. Lanier LL. NKG2D Receptor and Its Ligands in Host Defense. *Cancer Immunol Res* 2015;3(6):575-82.

29. Garrity D, Call ME, Feng J, et al. The activating NKG2D receptor assembles in the membrane with two signaling dimers into a hexameric structure. *Proc Natl Acad Sci U S A* 2005;102(21):7641-6.

30. Gilfillan S, Ho EL, Cella M, et al. NKG2D recruits two distinct adapters to trigger NK cell activation and costimulation. *Nat Immunol* 2002;3(12):1150-5.

31. Carapito R, Bahram S. Genetics, genomics, and evolutionary biology of NKG2D ligands. *Immunol Rev* 2015;267(1):88-116.

32. Raulet DH, Gasser S, Gowen BG, et al. Regulation of ligands for the NKG2D activating receptor. *Annu Rev Immunol* 2013;31:413-41.

33. Le Bert N, Gasser S. Advances in NKG2D ligand recognition and responses by NK cells. *Immunol Cell Biol* 2014;92(3):230-6.

34. Spear P, Wu M-R, Sentman M-L, et al. NKG2D ligands as therapeutic targets. *Cancer Immunity Archive* 2013;13:8.

35. Holdenrieder S, Stieber P, Peterfi A, et al. Soluble MICB in malignant diseases: analysis of diagnostic significance and correlation with soluble MICA. *Cancer Immunol Immunother* 2006;55(12):1584-9.

36. Salih HR, Rammensee HG, Steinle A. Cutting Edge: Down-Regulation of MICA on Human Tumors by Proteolytic Shedding. *J Immunol* 2002;169(8):4098-102.

37. Zhang T, Lemoi BA, Sentman CL. Chimeric NK-receptor-bearing T cells mediate antitumor immunotherapy. *Blood* 2005;106(5):1544-51.

For peer review only

38. Zhang T, Barber A, Sentman CL. Chimeric NKG2D modified T cells inhibit systemic T-cell lymphoma growth in a manner involving multiple cytokines and cytotoxic pathways. *Cancer Res* 2007;67(22):11029-36.

39. Barber A, Meehan KR, Sentman CL. Treatment of multiple myeloma with adoptively transferred chimeric NKG2D receptor-expressing T cells. *Gene Ther* 2011;18(5):509-16.

40. Barber A, Zhang T, DeMars LR, et al. Chimeric NKG2D receptor-bearing T cells as immunotherapy for ovarian cancer. *Cancer Res* 2007;67(10):5003-8.

41. Barber A, Zhang T, Sentman CL. Immunotherapy with Chimeric NKG2D Receptors Leads to Long-Term Tumor-Free Survival and Development of Host Antitumor Immunity in Murine Ovarian Cancer. *J Immunol* 2008;180:72-78.

42. Spear P, Barber A, Sentman CL. Collaboration of chimeric antigen receptor (CAR)-expressing T cells and host T cells for optimal elimination of established ovarian tumors. *Oncoimmunology* 2013;2(4):e23564.

43. Zhang T, Sentman CL. Mouse tumor vasculature expresses NKG2D ligands and can be targeted by chimeric NKG2D-modified T cells. *J Immunol* 2013;190(5):2455-63.

44. Barber A, Zhang T, Megli CJ, et al. Chimeric NKG2D receptor-expressing T cells as an immunotherapy for multiple myeloma. *Exp Hematol* 2008;36(10):1318-28.

45. Zhang T, Barber A, Sentman CL. Generation of antitumor responses by genetic modification of primary human T cells with a chimeric NKG2D receptor. *Cancer Res* 2006;66(11):5927-33.

46. Demoulin B, Cook WJ, Murad J, et al. Exploiting natural killer group 2D receptors for CAR T-cell therapy. *Future Oncol* 2017.

47. Spear P, Barber A, Rynda-Apple A, et al. NKG2D CAR T-cell therapy inhibits the growth of NKG2D ligand heterogeneous tumors. *Immunol Cell Biol* 2013;91(6):435-40.

For peer review only - http://bmjopen.bmj.com/site/about/guidelines.xhtml

48. Sentman ML, Murad JM, Cook WJ, et al. Mechanisms of Acute Toxicity in NKG2D Chimeric Antigen Receptor T Cell-Treated Mice. *J Immunol* 2016;197(12):4674-85.

49. Barber A, Rynda A, Sentman CL. Chimeric NKG2D expressing T cells eliminate immunosuppression and activate immunity within the ovarian tumor microenvironment. *J Immunol* 2009;183(11):6939-47.

50. Barber A, Sentman CL. Chimeric NKG2D T cells require both T cell- and host-derived cytokine secretion and perforin expression to increase tumor antigen presentation and systemic immunity. *J Immunol* 2009;183(4):2365-72.

51. Spear P, Barber A, Rynda-Apple A, et al. Chimeric antigen receptor T cells shape myeloid cell function within the tumor microenvironment through IFN-gamma and GM-CSF. *J Immunol* 2012;188(12):6389-98.

52. Sentman CL, Meehan KR. NKG2D CARs as cell therapy for cancer. *Cancer J* 2014;20(2):156-9.

53. Barber A, Sentman CL. NKG2D receptor regulates human effector T-cell cytokine production. *Blood* 2011;117(24):6571-81.

54. Tey SK. Adoptive T-cell therapy: adverse events and safety switches. *Clin Transl Immunology* 2014;3(6):e17.

55. Ertl HC, Zaia J, Rosenberg SA, et al. Considerations for the clinical application of chimeric antigen receptor T cells: observations from a recombinant DNA Advisory Committee Symposium held June 15, 2010. *Cancer Res* 2011;71(9):3175-81.

56. Minagawa K, Zhou X, Mineishi S, et al. Seatbelts in CAR therapy: How Safe Are CARS? *Pharmaceuticals (Basel)* 2015;8(2):230-49.

57. A first-in-human phase I trial of NKG2D chimeric antigen receptor-T cells in AML/MDS and multiple myeloma. 2016 ASCO Annual Meeting; 2016. J Clin Oncol.

For peer review only

58. Davila ML, Riviere I, Wang X, et al. Efficacy and toxicity management of 19-28z CAR T cell therapy in B cell acute lymphoblastic leukemia. *Sci Transl Med* 2014;6(224):224ra25.

59. Lee DW, Gardner R, Porter DL, et al. Current concepts in the diagnosis and management of cytokine release syndrome. *Blood* 2014;124(2):188-95.

60. Walker RE, Bechtel CM, Natarajan V, et al. Long-term in vivo survival of receptor-modified syngeneic T cells in patients with human immunodeficiency virus infection. *Blood* 2000;96(2):467-74.

61. Barrett DM, Liu X, Jiang S, et al. Regimen-specific effects of RNA-modified chimeric antigen receptor T cells in mice with advanced leukemia. *Hum Gene Ther* 2013;24(8):717-27.

62. Beatty GL, Haas AR, Maus MV, et al. Mesothelin-specific chimeric antigen receptor mRNA-engineered T cells induce anti-tumor activity in solid malignancies. *Cancer Immunol Res* 2014;2(2):112-20.

For peer review only - http://bmjopen.bmj.com/site/about/guidelines.xhtml

Footnotes

Acknowledgements

The delivery of successful clinical trials is primarily due to the excellence of the clinical partners with whom Celyad collaborates. The authors wish to thank all investigators in US and EU and all clinical managers for their valuable support and guidance. Finally, we thank all the patients and their families for participating to the study and for encouraging us to do this research.

Authors' contributions

CL, DG and FL conceived the study design, developed essential study documents and drafted the manuscript. BV, DG and FL participate in the coordination of the study. FP participated in the study design and the statistical review. AH, PA, AA, EVDN, GC, JPM, JB, DS, TK and KO participate in the management, will treat patients and conduct the clinical trial. MD gave support and guidance during the study design conception. All authors read, reviewed and approved the final manuscript.

Competing interests

CL, BV, DG and FL are employed by Celyad SA. JB and PA declare no disclosures. JPM has advisory roles with Merck Sharp and Dohme (uncompensated), Innate Pharma, Debio, Boehringer-Ingelheim, Nanobiotix. The THINK clinical trial is sponsored by Celyad SA.

Data sharing

Access to the dataset will only be considered when the primary results of the study have been published.

Funding

For peer review only - http://bmjopen.bmj.com/site/about/guidelines.xhtml

There are no funders to report for this submission.

Figure legends

Figure 1: The NKR-2 construct. NKR-2 design contains the full-length human NKG2D receptor linked to the signalling domain of CD3ζ that provides primary signalling (signal 1) to activate T-cells upon ligand binding. The naturally expressed adaptor molecule DAP10 provides secondary signalling (signal 2) which allows NKR-2 to work as a second-generation CAR-T.

Figure 2: NKR-2 different mechanisms of action. Apart from direct cytotoxicity against cancer cells, NKR-2 mode of action also involves reduction of blood vessel density indicative of an anti-angiogenic activity, modulation of the immunosuppressive tumour microenvironment and induction of a long-term anti-tumour specific memory immune response.

Figure 3: Overview of the study design. ORR= objective response rate, RecD = recommended dose.

Figure 4: Overview of study phases. NKR-2 will be infused on Day 1, 15 and 29. Tumour assessment will be done by tumour imaging and blood sampling for solid tumours and tumour imaging, blood, urine and bone marrow sampling for haematological tumours. D = day, M = Month, BM = bone marrow.

For peer review only - http://bmjopen.bmj.com/site/about/guidelines.xhtml

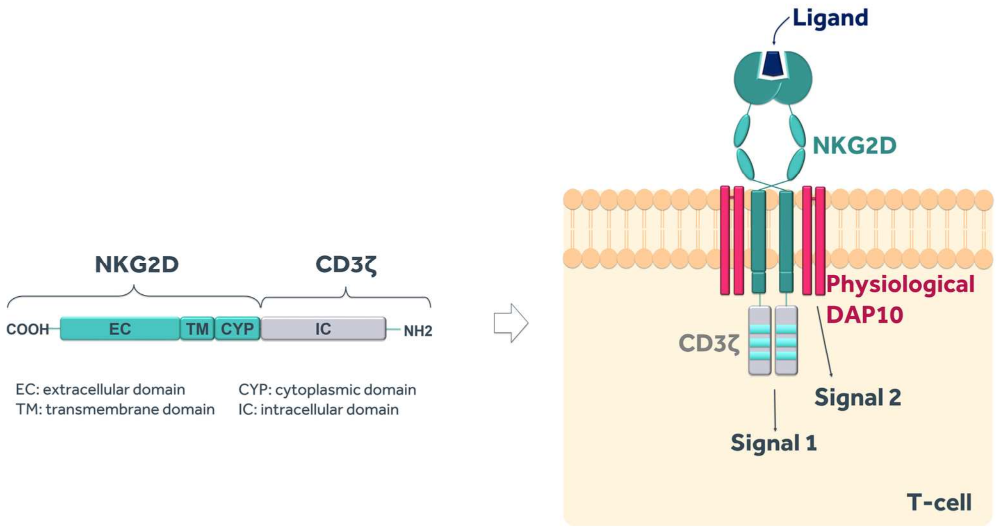

Figure 1: The NKR-2 construct. NKR-2 design contains the full-length human NKG2D receptor linked to the signalling domain of CD3ζ that provides primary signalling (signal 1) to activate T-cells upon ligand binding. The naturally expressed adaptor molecule DAP10 provides secondary signalling (signal 2) which allows NKR-2 to work as a second-generation CAR-T.

161x86mm (300 x 300 DPI)

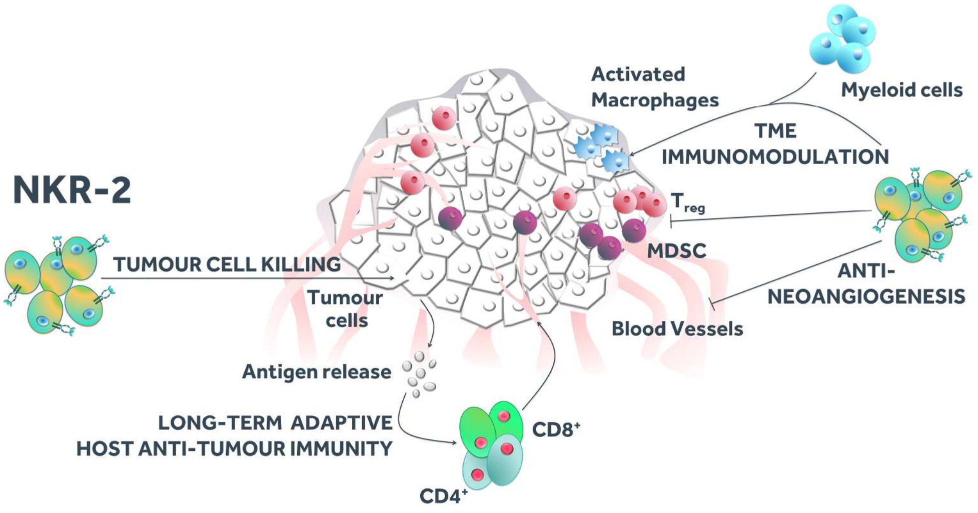

Figure 2: NKR-2 different mechanisms of action. Apart from direct cytotoxicity against cancer cells, NKR-2 mode of action also involves reduction of blood vessel density indicative of an anti-angiogenic activity, modulation of the immunosuppressive tumour microenvironment and induction of a long-term anti-tumour specific memory immune response.

233x120mm (300 x 300 DPI)

For peer review only

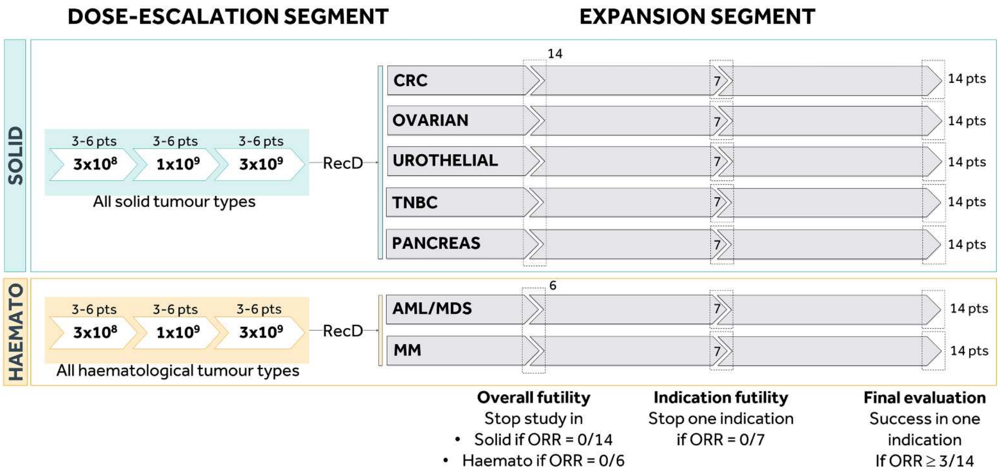

Figure 3: Overview of the study design. ORR= objective response rate, RecD = recommended dose.

242x118mm (300 x 300 DPI)

For peer review only

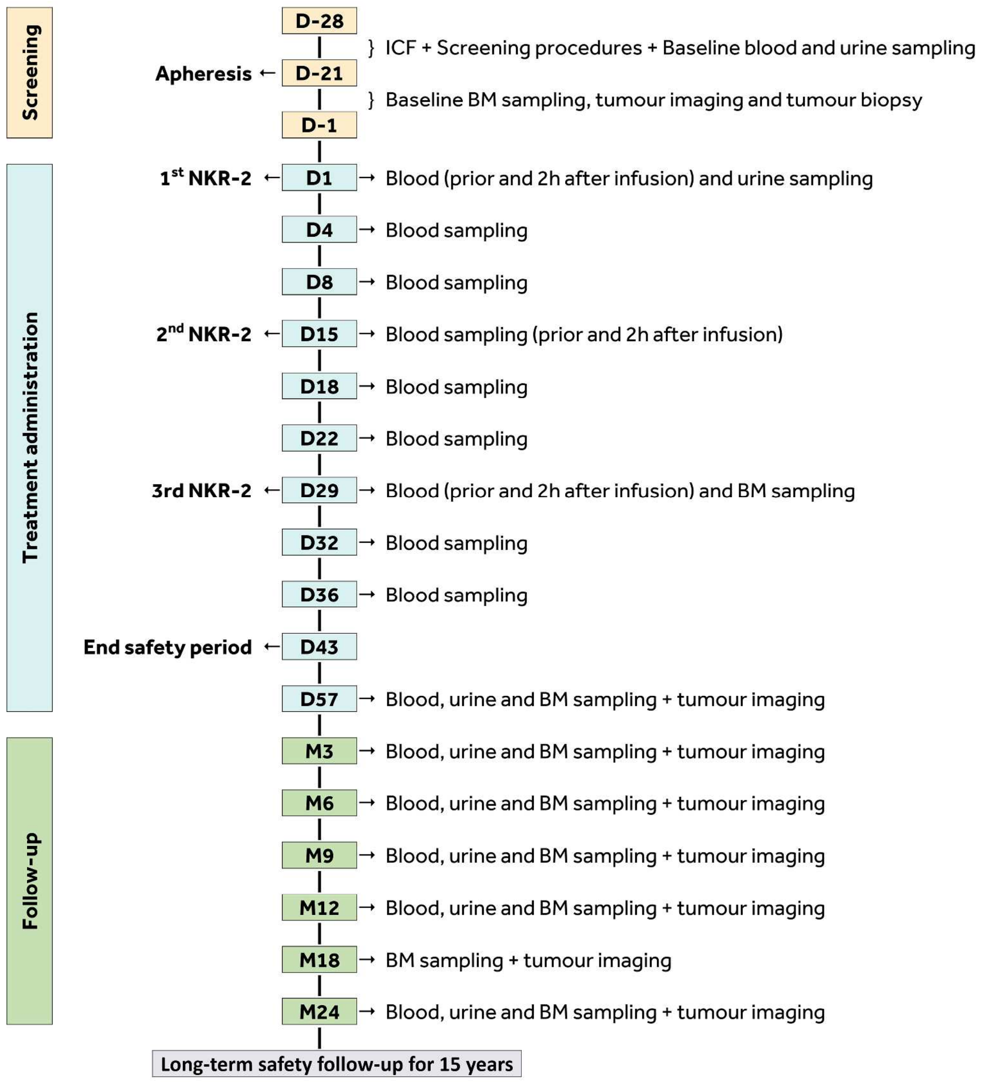

Figure 4: Overview of study phases. NKR-2 will be infused on Day 1, 15 and 29. Tumour assessment will be done by tumour imaging and blood sampling for solid tumours and tumour imaging, blood, urine and bone marrow sampling for haematological tumours. D = day, M = Month, BM = bone marrow.

326x355mm (300 x 300 DPI)

SPIRIT 2013 Checklist: Recommended items to address in a clinical trial protocol and related documents*

Section/item	Item No	Description	Addressed on page number
**Administrative information**			
Title	1	Descriptive title identifying the study design, population, interventions, and, if applicable, trial acronym	Page 1
Trial registration	2a	Trial identifier and registry name. If not yet registered, name of intended registry	Page 3
	2b	All items from the World Health Organization Trial Registration Data Set	N/A
Protocol version	3	Date and version identifier	Page 30
Funding	4	Sources and types of financial, material, and other support	Page 39
Roles and responsibilities	5a	Names, affiliations, and roles of protocol contributors	Page 39
	5b	Name and contact information for the trial sponsor	Page 1
	5c	Role of study sponsor and funders, if any, in study design; collection, management, analysis, and interpretation of data; writing of the report; and the decision to submit the report for publication, including whether they will have ultimate authority over any of these activities	Page 29
	5d	Composition, roles, and responsibilities of the coordinating centre, steering committee, endpoint adjudication committee, data management team, and other individuals or groups overseeing the trial, if applicable (see Item 21a for data monitoring committee)	Sections 12.5.2 and 12.5.3 of the full-length protocol

For peer review only - http://bmjopen.bmj.com/site/about/guidelines.xhtml

**Introduction**			
Background and rationale	6a	Description of research question and justification for undertaking the trial, including summary of relevant studies (published and unpublished) examining benefits and harms for each intervention	Pages 6-10
	6b	Explanation for choice of comparators	N/A
Objectives	7	Specific objectives or hypotheses	Page 10
Trial design	8	Description of trial design including type of trial (eg, parallel group, crossover, factorial, single group), allocation ratio, and framework (eg, superiority, equivalence, noninferiority, exploratory)	Page 14, Figure 3
**Methods: Participants, interventions, and outcomes**			
Study setting	9	Description of study settings (eg, community clinic, academic hospital) and list of countries where data will be collected. Reference to where list of study sites can be obtained	Page 12
Eligibility criteria	10	Inclusion and exclusion criteria for participants. If applicable, eligibility criteria for study centres and individuals who will perform the interventions (eg, surgeons, psychotherapists)	Page 13
Interventions	11a	Interventions for each group with sufficient detail to allow replication, including how and when they will be administered	Figure 4
	11b	Criteria for discontinuing or modifying allocated interventions for a given trial participant (eg, drug dose change in response to harms, participant request, or improving/worsening disease)	Page 17 + Section 6.4.1 of the full-length protocol
	11c	Strategies to improve adherence to intervention protocols, and any procedures for monitoring adherence (eg, drug tablet return, laboratory tests)	Section 12.1 of the full-length protocol
	11d	Relevant concomitant care and interventions that are permitted or prohibited during the trial	Section 6.5.1 of the full-length protocol

For peer review only - http://bmjopen.bmj.com/site/about/guidelines.xhtml

Outcomes	12	Primary, secondary, and other outcomes, including the specific measurement variable (eg, systolic blood pressure), analysis metric (eg, change from baseline, final value, time to event), method of aggregation (eg, median, proportion), and time point for each outcome. Explanation of the clinical relevance of chosen efficacy and harm outcomes is strongly recommended	Page 16
Participant timeline	13	Time schedule of enrolment, interventions (including any run-ins and washouts), assessments, and visits for participants. A schematic diagram is highly recommended (see Figure)	Figure 4
Sample size	14	Estimated number of participants needed to achieve study objectives and how it was determined, including clinical and statistical assumptions supporting any sample size calculations	Page 14, 15 and 22
Recruitment	15	Strategies for achieving adequate participant enrolment to reach target sample size	Page 18
**Methods: Assignment of interventions (for controlled trials)**			
Allocation:			
Sequence generation	16a	Method of generating the allocation sequence (eg, computer-generated random numbers), and list of any factors for stratification. To reduce predictability of a random sequence, details of any planned restriction (eg, blocking) should be provided in a separate document that is unavailable to those who enrol participants or assign interventions	N/A
Allocation concealment mechanism	16b	Mechanism of implementing the allocation sequence (eg, central telephone; sequentially numbered, opaque, sealed envelopes), describing any steps to conceal the sequence until interventions are assigned	N/A
Implementation	16c	Who will generate the allocation sequence, who will enrol participants, and who will assign participants to interventions	N/A
Blinding (masking)	17a	Who will be blinded after assignment to interventions (eg, trial participants, care providers, outcome assessors, data analysts), and how	N/A
	17b	If blinded, circumstances under which unblinding is permissible, and procedure for revealing a participant's allocated intervention during the trial	N/A
**Methods: Data collection, management, and analysis**			

For peer review only - http://bmjopen.bmj.com/site/about/guidelines.xhtml

Data collection methods	18a	Plans for assessment and collection of outcome, baseline, and other trial data, including any related processes to promote data quality (eg, duplicate measurements, training of assessors) and a description of study instruments (eg, questionnaires, laboratory tests) along with their reliability and validity, if known. Reference to where data collection forms can be found, if not in the protocol	Section 12 of the full-length protocol
	18b	Plans to promote participant retention and complete follow-up, including list of any outcome data to be collected for participants who discontinue or deviate from intervention protocols	Page 17
Data management	19	Plans for data entry, coding, security, and storage, including any related processes to promote data quality (eg, double data entry; range checks for data values). Reference to where details of data management procedures can be found, if not in the protocol	Sections 13.1 and 13.4 of the full-length protocol
Statistical methods	20a	Statistical methods for analysing primary and secondary outcomes. Reference to where other details of the statistical analysis plan can be found, if not in the protocol	Page 20-22 + Statistical plan
	20b	Methods for any additional analyses (eg, subgroup and adjusted analyses)	Pages 20-22 + Statistical plan (appendix to the original protocol)
	20c	Definition of analysis population relating to protocol non-adherence (eg, as randomised analysis), and any statistical methods to handle missing data (eg, multiple imputation)	Pages 20-22 + Statistical plan
**Methods: Monitoring**			
Data monitoring	21a	Composition of data monitoring committee (DMC); summary of its role and reporting structure; statement of whether it is independent from the sponsor and competing interests; and reference to where further details about its charter can be found, if not in the protocol. Alternatively, an explanation of why a DMC is not needed	Page 28
	21b	Description of any interim analyses and stopping guidelines, including who will have access to these interim results and make the final decision to terminate the trial	Pages 19-22
Harms	22	Plans for collecting, assessing, reporting, and managing solicited and spontaneously reported adverse events and other unintended effects of trial interventions or trial conduct	Page 18

For peer review only - http://bmjopen.bmj.com/site/about/guidelines.xhtml

Auditing	23	Frequency and procedures for auditing trial conduct, if any, and whether the process will be independent from investigators and the sponsor	Page 28
**Ethics and dissemination**			
Research ethics approval	24	Plans for seeking research ethics committee/institutional review board (REC/IRB) approval	N/A (approved)
Protocol amendments	25	Plans for communicating important protocol modifications (eg, changes to eligibility criteria, outcomes, analyses) to relevant parties (eg, investigators, REC/IRBs, trial participants, trial registries, journals, regulators)	Page 28
Consent or assent	26a	Who will obtain informed consent or assent from potential trial participants or authorised surrogates, and how (see Item 32)	Page 29
	26b	Additional consent provisions for collection and use of participant data and biological specimens in ancillary studies, if applicable	N/A
Confidentiality	27	How personal information about potential and enrolled participants will be collected, shared, and maintained in order to protect confidentiality before, during, and after the trial	Page 29
Declaration of interests	28	Financial and other competing interests for principal investigators for the overall trial and each study site	Page 30
Access to data	29	Statement of who will have access to the final trial dataset, and disclosure of contractual agreements that limit such access for investigators	Page 30
Ancillary and post-trial care	30	Provisions, if any, for ancillary and post-trial care, and for compensation to those who suffer harm from trial participation	Page 17
Dissemination policy	31a	Plans for investigators and sponsor to communicate trial results to participants, healthcare professionals, the public, and other relevant groups (eg, via publication, reporting in results databases, or other data sharing arrangements), including any publication restrictions	Page 29
	31b	Authorship eligibility guidelines and any intended use of professional writers	Page 29
	31c	Plans, if any, for granting public access to the full protocol, participant-level dataset, and statistical code	Page 39

For peer review only - http://bmjopen.bmj.com/site/about/guidelines.xhtml

Appendices

Informed consent materials	32	Model consent form and other related documentation given to participants and authorised surrogates	Appendix to the original protocol
Biological specimens	33	Plans for collection, laboratory evaluation, and storage of biological specimens for genetic or molecular analysis in the current trial and for future use in ancillary studies, if applicable	Appendices to the original protocol

*It is strongly recommended that this checklist be read in conjunction with the SPIRIT 2013 Explanation & Elaboration for important clarification on the items. Amendments to the protocol should be tracked and dated. The SPIRIT checklist is copyrighted by the SPIRIT Group under the Creative Commons "Attribution-NonCommercial-NoDerivs 3.0 Unported" license.

For peer review only

For peer review only - http://bmjopen.bmj.com/site/about/guidelines.xhtml
